# Molecular properties and diagnostic potential of monoclonal antibodies targeting cytotoxic α-synuclein oligomers

**DOI:** 10.1038/s41531-024-00747-6

**Published:** 2024-07-29

**Authors:** Janni Nielsen, Johanne Lauritsen, Jannik N. Pedersen, Jan S. Nowak, Malthe K. Bendtsen, Giulia Kleijwegt, Kaija Lusser, Laia C. Pitarch, Julián V. Moreno, Matthias M. Schneider, Georg Krainer, Louise Goksøyr, Paul Khalifé, Sanne Simone Kaalund, Susana Aznar, Magnus Kjærgaard, Vita Sereikaité, Kristian Strømgaard, Tuomas P. J. Knowles, Morten Agertoug Nielsen, Adam F. Sander, Marina Romero-Ramos, Daniel E. Otzen

**Affiliations:** 1 Interdisciplinary Nanoscience Center (iNANO), Aarhus University, Aarhus C, Denmark; 2 DANDRITE & Department of Biomedicine, Aarhus University, Aarhus C, Denmark; 3 Yusuf Hamied Department of Chemistry, University of Cambridge, Cambridge, UK; 4 Institute of Molecular Biosciences, University of Graz, Graz, Austria; 5 Centre for Medical Parasitology at the Department of Immunology and Microbiology, University of Copenhagen, Copenhagen, Denmark; 6 Centre for Neuroscience and Stereology, Bispebjerg and Frederiksberg Hospital, Copenhagen, Denmark; 7 Department of Molecular Biology and Genetics, Aarhus University, Aarhus C, Denmark; 8 Department of Drug Design and Pharmacology, University of Copenhagen, Copenhagen, Denmark

**Keywords:** Molecular biology, Immunology

## Abstract

α-Synuclein (α-syn) accumulates as insoluble amyloid but also forms soluble α-syn oligomers (αSOs), thought to be even more cytotoxic than fibrils. To detect and block the unwanted activities of these αSOs, we have raised 30 monoclonal antibodies (mAbs) against different forms of αSOs, ranging from unmodified αSOs to species stabilized by lipid peroxidation products and polyphenols, αSOs formed by C-terminally truncated α-syn, and multivalent display of α-syn on capsid virus-like particles (cVLPs). While the mAbs generally show a preference for αSOs, they also bind fibrils, but to variable extents. Overall, we observe great diversity in the mAbs’ relative affinities for monomers and αSOs, varied requirements for the C-terminal extension of α-syn, and only a modest effect on α-syn fibrillation. Several mAbs show several orders of magnitude preference for αSOs over monomers in in-solution studies, while the commercial antibody MJF14 only bound 10-fold more strongly to αSOs than monomeric α-syn. Gratifyingly, seven mAbs almost completely block αSO permeabilization of membrane vesicles. Five selected mAbs identified α-syn-related pathologies like Lewy bodies (LBs) and Lewy Neurites, as well as Glial Cytoplasmic Inclusions in postmortem brains from people diagnosed for PD, dementia with LBs or multiple system atrophy, although to different extents. Three mAbs were particularly useful for pathological evaluation of postmortem brain human tissue, including early stages of PD. Although there was no straightforward connection between the mAbs’ biophysical and immunohistochemical properties, it is encouraging that this comprehensive collection of mAbs able to recognize different aggregated α-syn species in vitro also holds diagnostic potential.

## Introduction

The group of diseases called synucleinopathies presents an accumulation of intracellular aggregates containing the 140-residue protein α-synuclein (α-syn) in different parts of the brain and central nervous system^1,2^, as well as the peripheral and enteric nervous system^3^. Parkinson’s Disease (PD) is the most prominent example; other members include Dementia with Lewy Bodies (DLB) and Multiple System Atrophy (MSA). Although α-syn aggregation unites these diseases as a “centralizing micro-pathological change”^2^, the manifestations vary among the different synucleinopathies in terms of clinical symptoms and targeted brain cells (neurons or oligodendroglia) and brain regions. Furthermore, there are different types of intracellular aggregates, i.e. spherical Lewy bodies (LBs) in PD but less structured glial cytoplasmic inclusions (GCIs) in MSA^4^. It has been proposed that PD α-syn pathology itself can initiate and spread in different ways, a recent hypothesis suggesting two subtypes: either brain-first (where pathology initiates in the brain) or body-first (where α-syn aggregation starts in the gut and is then transmitted to other anatomically connected areas in the brain)^5,6^. This spectrum of pathophysiology likely reflects the diversity of α-syn’s “social history”. Monomeric α-syn is intrinsically disordered and only forms an organized structure when it binds to other partners, especially lipids^7^. While its “true” function is unclear, it is associated with synaptic trafficking^8,9^ and forms α-helical structures on anionic membranes^10^. However, it also has a high propensity to aggregate into β-sheet rich structures in a complex sequence of events that generates soluble α-synuclein oligomers (αSOs) and insoluble fibrils. The pathogenesis of synucleinopathies was originally suggested to be linked to α-syn fibrils and LB formation, but this was confounded by the lack of correlation between neurodegeneration and LB pathology^11^ and the fact that healthy brains could harbor LBs^12^ while cellular dysfunction seems to occur prior to any overt α-syn aggregation. Indeed, the brains from some PD patients are free of LBs^13^. Focus has subsequently switched towards αSOs which form spontaneously in vitro^14^, are found in biological fluids such as cerebrospinal fluid^15^ and blood^16^, and appear to correlate with PD progression^17^. In addition, proximity ligation assays have also highlighted the presence of pre-LB structures, possibly corresponding to αSO species^18^.

Operationally, αSOs can be defined as soluble multimeric species that are not fibrillar and could thus span everything from dimers to complexes of up to 50–60 monomers. In reality, the predominant αSO species, which accumulates in vitro under aggregation-promoting conditions, contains ~30 monomers and forms a compact core and a dynamic and disordered shell^19–21^. Interestingly, αSO formation is off-pathway to fibrillation, i.e. the αSO needs to dissociate to monomers before fibrils can be formed. Moreover, αSOs even have the capability to block fibrillation to some extent^21^. αSOs permeabilize membranes much more efficiently than both the monomer and the fibrillated state^22,23^. In the cell, this translates into disruption of membrane integrity^22,24^, leading to dysfunction in the endoplasmic reticulum^25,26^ and mitochondria^27^ as well as other cellular insults^28^. These aspects make αSOs an important therapeutic target. αSOs can be formed under several different conditions in vitro, e.g. in the presence of lipid peroxidation products such as docosahexanoic acid (DHA)^29^, 4-hydroxynonenal (HNE)^29^ and 4-oxononenal (ONE)^30,31^, but despite variations in structure and size, they all show the same ability to bind biochemical targets such as glutamatergic synapses^29^. The correspondence between these αSOs formed in vitro and their cellular counterparts remains unclear, however. αSOs are found in many different biological fluids of synucleinopathy patients^32,33^, and their levels correlate with disease progression^17,32^. They could promote disease progression through exocytosis^34^ followed by e.g. receptor-mediated uptake^35^, trans-synaptic propagation^36^, simple endocytosis^37^, or by promoting inflammation^38^. All these aspects motivate development of protocols and tools to detect and characterize αSOs as thoroughly and specifically as possible.

Detection and characterization of αSOs is largely based on supposedly αSO-specific antibodies, although there are also promising alternatives using proximity ligation assays^18^ or αSO-specific peptides^39^ which can be combined with plasmonic devices^40^. Antibodies have the advantage that they are potentially therapeutic and can be engineered for improved specificity or affinity^41^ – provided challenges in crossing the blood-brain barrier can be overcome^42^. The widespread use of antibodies underscores the need to thoroughly characterize their binding properties before making conclusions about the aggregation state of the α-syn species they detect, bearing in mind the great physical and chemical diversity of αSOs we can expect to find in vivo. Specifically, it is important to evaluate the antibodies’ relative affinities for monomers, αSOs and fibrils. A recent, very thorough study by Lashuel and coworkers revealed that none of 18 examined α-syn antibodies (of which 16 were claimed to bind only to aggregated α-syn) are entirely specific for one species. All but two (5G4 and SYN-1) also bind to monomeric α-syn (though some bind more weakly to the monomer) and none of the antibodies differentiate between αSOs and fibrils^43^. This conclusion was reached after comparing the antibodies’ ability to bind an array of αSOs and fibrillar α-syn using immunoblotting (slot and Western blots), single-molecule array ELISA assays, and Surface Plasmon Resonance analysis^43^. This lack of specificity may also reflect the way these antibodies were raised: of 44 monoclonal antibodies (mAbs) listed in a recent excellent review by El-Agnaf and coworkers^1^, 22 had been raised against recombinant full-length monomeric α-syn (all produced in mice, except three from rabbits and one from sheep) and eight against fragments of α-syn ranging from small fragments from the termini to slightly trimmed α-syn mutants. In addition, there were eight reports using chemically modified α-syn (four nitrated and three oxidized full-length α-syn as well as peptides containing phosphorylated Ser129^44^). Only a small minority had used pre-aggregated α-syn; these included two examples of αSOs stabilized by HNE, one gradient-purified fibril sample, one mixture of monomers and aggregates, one cross-linked fibril sample and one example of LB aggregates^45^. mAbs raised against “native” α-syn do not distinguish between monomers and other species (with one reported exception^46^). Specificity for certain conformations is obviously critical for identifying potentially toxic species, but this was (apart from ref. ^46^) only obtained when the antigen was aggregated α-syn (either αSO or fibril).

It is not surprising that it is difficult to raise mAbs that only bind to αSOs and not fibrils or vice versa. Aggregation of α-syn involves intermolecular contacts which are likely similar between αSOs and fibrils; both species depend particularly on the NAC region for these interactions^19^, and a recent cryoEM study of a stabilized αSO was able to thread a fibrillar backbone structure through part of the αSO^47^. Nevertheless, the major αSO species that accumulates in vitro is generally very stable and self-contained^21^, i.e. it is not simply a miniature fibril and will therefore likely possess structural features (and thus epitopes) not found in the fibril. Given the need for mAbs with as much preference for the αSOs as possible^48^, we have carried out a concerted effort to raise mAbs against a broad range of different forms of αSO, including unmodified αSOs, αSOs stabilized by lipid peroxidation products and polyphenols, αSOs formed by C-terminally truncated α-syn, and multivalent display of α-syn on capsid virus-like particles (cVLPs). Here, we describe the general biophysical and immunohistochemical properties of 30 such anti-αSO mAbs. We conclude that no mAbs show absolute specificity for the αSOs but do manifest a great diversity in their relative affinities for monomers and αSOs, varied requirements for the C-terminal extension of α-syn and only a modest effect on α-syn fibrillation. Gratifyingly, seven of the mAbs almost completely block αSO permeabilization of membrane vesicles, which may be considered a simple proxy for membrane-mediated cell toxicity. Sixteen mAbs were analyzed for binding to brain sections from rats overexpressing human α-syn in the nigro-striatal pathway, and the 5 most promising were further investigated for their ability to recognize α-syn aggregates in human brain tissue. All five mAbs identified α-syn-related pathologies like LBs, Lewy neurites (LNs), and GCIs in *postmortem* brains from people diagnosed with PD, DLB, and MSA, although to different extents.

## Results

### Immunization and selection of antibodies for purification via ELISA

To raise mAbs specific for the aggregated (and preferably oligomeric) state of α-syn, mice were separately immunized with different forms of αSOs, either using a truncated (residues 1–125) α-syn (1–125), unmodified form or stabilized using the lipid peroxidation products ONE or HNE or the polyphenol EGCG. Note that α-syn 1–125 is the shortest construct able to form αSOs on its own and with a shift in the αSO structure compared to full-length α-syn, leading to a thicker shell of disordered residues and a larger number of monomers per αSO^49^. In addition, to display α-syn we exploited a vaccine platform based on a cVLP^50^; here, a SpyCatcher domain is fused to the N-terminus of the *Acinetobacter phage* AP205 capsid protein which subsequently self-assembles to a cVLP consisting of 180 subunits, each of which surface displays a SpyCatcher domain. In parallel, the 13-residue SpyTag peptide was fused to the N-terminus of α-syn, enabling high-density and unidirectional cVLP display of multiple copies of α-syn on each cVLP, mediated by the covalent SpyTag-SpyCatcher interaction^51^. For our constructs, we used α-syn truncated at the C-terminus to give lengths of 100 and 120 residues, respectively (full-length α-syn consists of 140 residues but this construct did not couple well to the cVLPs). Table 1 provides an overview of the different αSOs and associated mice.

**Table 1:** Antigens used to immunize mice

Mouse numbering	Antigen used for immunization
Mouse 1–4	spy-VLP displaying α-syn 1-120
Mouse 5–8	spy-VLP displaying α-syn 1-100
Mouse 9–12	αSOs made from truncated α-syn (residues 1-125)
Mouse 13–16	Unmodified αSO
Mouse 17 and 20	HNE-αSO
Mouse 18–19	EGCG-αSO
Mouse 21–22	ONE-αSO

Mice were monitored for production of antibodies recognizing α-syn in either the monomeric or aggregated state using an indirect ELISA assay, in which the surfaces of the different plate wells were coated with α-syn (monomer, αSO or fibril), after which antibody samples were added followed by secondary antibodies. All classes of antigens gave rise to antibodies that bound all three α-syn states in these tests. The antibodies leading to the highest ELISA responses were selected for sub-cloning by dilution steps to obtain mAbs. mAbs were purified from 0.5 L portions of cell culture supernatant, harvested from 150-mL flasks. A representative SDS-PAGE analysis of purified mAbs is shown in Supplementary Fig. S1a, demonstrating acceptable purity for all purified mAbs. Yields ranged from 8 to 16 mg per 0.5 L. Consistent with the ability to obtain reasonable levels of purified mAbs, they were all very thermostable according to differential scanning fluorimetry. Thermal inflection points for all purified mAbs were around 80^o^C, except 08-9E4-E3 (75.8 ^o^C) and 10-9C8-C1 (86.9 ^o^C) (Table 2, column C).

**Table 2:** Parameters describing affinity of different monoclonal antibodies (mAbs) towards α-synuclein monomers and oligomers

The 16 mAbs selected for immunohistochemical analysis are shown either in green (11) or blue (5); these latter 5 were subsequently chosen for a more in-depth study. *SPR* surface plasmon resonance.

^a^The first number: mouse used for the immunization. Subsequent numbers and letters: wells from which clones obtained in ELISA screening.

^b^*NB* no binding.

^c^Decrease in size to R_h_ of monomeric α-syn upon titration of mAbs at concentrations above 2 nM for 16−9E5, 15.6 nM for 20-5H12-C10, and 250 nM for 20-9E2-B8.

^d^Association into large complexes (R_h_ > 15 nm) upon titration above 16 nM and K_D_ may be overestimated.

^e^t½ in absence of antibodies: 35.7 h. Errors ca. 10%. Carried out with 1 mg/ml α-syn monomer and beads while shaking.

^e^Calculated according to Eq. 1. Values of 0 also include negative values. Errors ca. 10%.

^f^Not applicable because mAb and αSO together led to the same release as αSO alone.

^g^Relatively low ELISA signal (20–40% of normal level) when bound in the absence of monomeric α-syn.

^h^Carried out using 0.3 mg/ml α-syn monomer, 0.3 mg/ml mAb and 0.7% (2.1 µg/ml) α-syn seeds. t½ in the absence of mAb: 19.3 h. Errors ca. 10%.

### Limitations in the use of ELISA assays for mAb-αSO interaction studies and use of competition assays

**Fig. 1:**
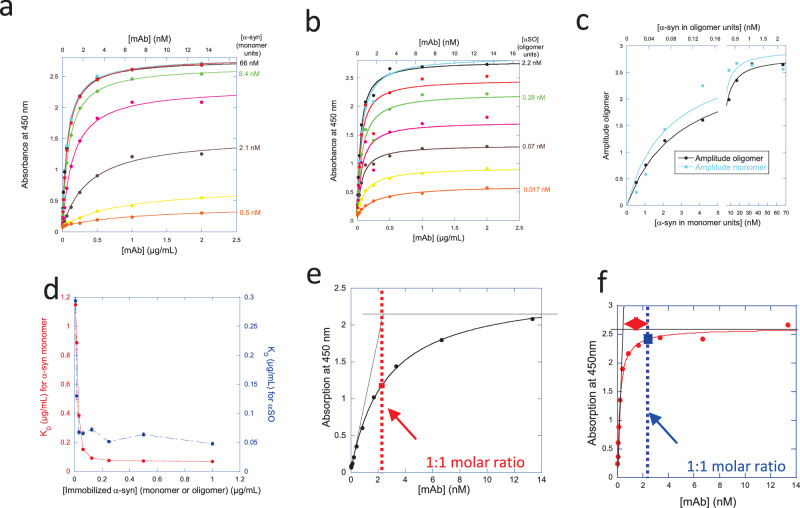
Examples of ELISA assays with monoclonal antibodies (mAbs). Indirect ELISA assays showing the extent of binding of the mAb 19-2C3-F10 to different fixed amounts of immobilized (**a**) α-syn monomers or (**b**) αSOs. **c** Summary of the data in panels **a** and **b** as a function of α-syn concentration in monomer units. **d** The apparent *K*_D_ values obtained from fits of data in panels **a** and **b** are fitted to a simple binding isotherm, showing a steep increase in apparent affinity with the concentration of immobilized antigen. Estimation of stoichiometry based on intercept between initial linear increase and the plateau value leads to (**e**) a 1:1 molar ratio for the antibody 03-9H9, but (**f**) 1 mAb per αSO for the antibody 10-9C8-C1.

For a general assessment of the purified mAbs binding preferences, we carried out additional measurements with indirect ELISA assays. Typical results are shown in Fig. 1 in which the mAb 19-2C3-F10 is tested on immobilized α-syn monomer (Fig. 1a) and αSOs (Fig. 1b). However, we decided only to use these ELISA data for initial screening for four reasons:

(1) The two ELISA curves for binding to monomer and αSO are broadly similar for most mAbs, even though subsequent FIDA results (see below) in many cases reveal a much greater affinity of the mAb for αSOs than for α-syn monomers.

(2) The signal change (amplitude) in these ELISA assays clearly does not scale linearly with the amount of immobilized material (Fig. 1c) but shows a strong hyperbolic dependence. At high signal levels, there are obvious limitations in optical read-outs, which saturate at absorption levels around 2.7–2.8. However, even at absorption levels well below 2, a plot of the signal change (amplitude) versus α-syn concentration shows a clear deviation from linearity (Fig. 1c). Since the mAb is present in excess compared to α-syn (whether monomer or αSO) at the plateau levels, we would expect saturation of all available α-syn binding sites. That should lead to a linear or proportional increase in signal with increasing amounts of α-syn, but this is not the case. In turn, this prevents us from using ELISA data to estimate the concentration of mAb:α-syn and mAB:αSO complexes, which are otherwise needed for quantitative analysis of these curves. A possible explanation for this observation lies in the fact that the α-syn species interact non-specifically upon surface immobilization. Similar observations have previously been attributed to electrostatic interaction between the negatively charged α-syn surface and accumulation of positive charge on binding partners^52^.

(3) At most concentrations of immobilized α-syn except the very highest, the mAbs are provided in excess relative to α-syn monomer/αSO at virtually all data points, so we can safely assume that the total concentration of mAbs is the same as the free concentration of mAb. This allows us to estimate apparent *K*_D_ values for mAb association to α-syn, using [mAb] as the variable (free) ligand concentration and assuming saturation of all α-syn binding sites. However, these *K*_D_ values, which should in principle remain constant, varied strongly and systematically with the amount of immobilized antigen (Fig. 1d). In most cases it declines, but in other cases, it increases.

(4) The stoichiometry can be crudely estimated as the intercept between the initial slope at low mAb concentrations and the plateau level, and this leads to a value of ~1:1 for most mAb: αSO complexes (Fig. 1e). However, in several cases the plateau is reached at extremely low mAb concentrations, suggesting that each mAb can bind to multiple αSOs (a naïve analysis of the data in Fig. 1f suggests 1 mAb per 4 αSOs), which is difficult to rationalize.

All these considerations indicate that ELISA assays do not provide a reliable way to estimate the affinity and stoichiometry of complexes between mAbs and α-syn monomer and αSO. We attribute this both to occlusion of potential binding sites on immobilized species as well as structural changes caused by the binding of flexible regions of α-syn and αSOs to the surface of the ELISA plates. Furthermore, as the exact concentrations of immobilized substrate cannot be quantified, all readouts are semi-quantitative only^53^.

**Fig. 2:**
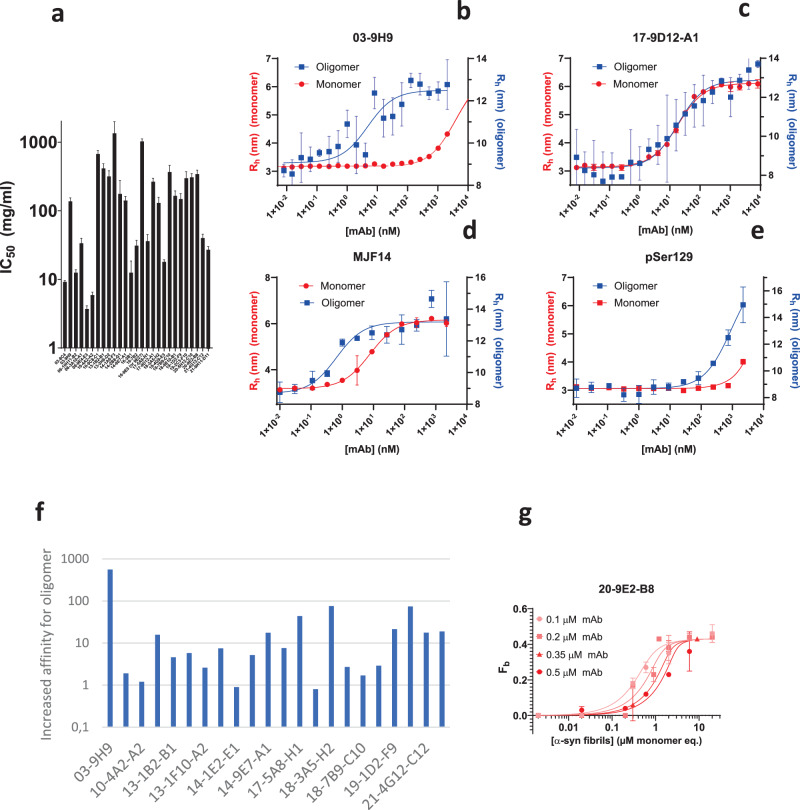
Antibody-αSO affinities measured in different ways. **a** Values of IC_50_ measured for 27 different mAbs in ELISA competition assays using immobilized αSOs with monomeric α-syn present in the mobile phase. Errors from binding fits. **b**–**e** FIDA titration plots for binding of α-syn monomer and αSO to two different mAbs produced in this study (03-9H9 and 17-9D12-A1) and two commercial antibodies (MJF14 and pSer129). Errors from fits to individual FIDA curves. Associated *K*_D_ values shown in Table 2 for in-house mAbs. For MJF14, the *K*_D_ for αSOs and monomeric α-syn are 0.42 and 3.92 nM, respectively; for pSer129 the values are 0.455 and 3.0 µM. **f** The increased affinity of mAbs for αSOs relative to α-syn based on the ratio *K*_D_^monomer^/ *K*_D_^αSO^. Data summarized in Table 2. **g** Microfluidic diffusional sizing (MDS) measurement of the binding of antibodies to α-syn fibrils, here exemplified with mAb 20-9E2-B8. Errors from fits to individual MDS curves. Data summarized in Table 3. Errors are standard errors of the mean based on triplicates.

In a complementary approach, we instead tested the purified mAbs in competition assays in which the mAb was incubated (i.e. preadsorbed as described elsewhere^54^) with 0-2 mg/ml monomeric α-syn before exposure to αSOs immobilized on an ELISA plate (raw binding data provided in Fig. S1b**)**. In this way, monomeric α-syn was prevented from binding to the blocked well surfaces and could only encounter mAbs in solution, avoiding surface-induced structural changes for this state (though not for the αSO). Relative binding affinities at 1 mg/ml monomer showed significant differences between the different mAbs’ abilities to retain αSO binding in the presence of monomeric α-syn. For a more quantitative evaluation, we fitted the data with a simple binding isotherm in which full binding is assumed to lead to complete displacement of the mAb from the ELISA plate. We designate the apparent dissociation constant IC_50_, which corresponds to the amount of monomer required to displace 50% of the bound mAb (values are provided in Table 2 column D and shown graphically in Fig. 2a). The larger the IC_50_, the more difficult it is for monomeric α-syn to displace the mAb from the αSO and by inference the greater the mAb’s preference for αSOs. IC_50_ values ranged from as high as 1.4 mg/ml for the mAb 14-1A6-F3 to values below 10 µg/ml for mAb 08-9E4-E3. The average value for all 26 tested mAbs is 248 ± 324 µg/ml, where the large standard error highlights the great variation in relative affinities for αSOs versus monomeric α-syn.

While we would have preferred to carry out similar competition assays using immobilized fibrils and αSOs in solution to gauge the relative affinities of the antibodies for αSOs versus fibrillar α-syn, the amounts of αSO needed to screen our 30 antibodies proved prohibitive. Furthermore, ELISA on α-syn fibrils has been shown to render inconclusive results^52^. We also tested the different antibodies for their ability to bind immobilized Aβ40 peptide and recombinant full-length tau protein but found no significant binding in our ELISA assays (data not shown).

### FIDA studies show general preference of mAbs for αSOs over monomeric α-syn in solution

For a direct assessment of in-solution affinities between antibodies and soluble α-syn species which avoids surface-binding artifacts, we turned to Flow-Induced Dispersion Analysis (FIDA). In this technique, the hydrodynamic radius *R*_h_ of a fluorescently labeled species is determined by its diffusion behavior in solution in a thin capillary tube^55^. Binding of the labelled species to a non-labelled binding partner will increase its *R*_h_ value in a manner reflecting the affinity between the two species, allowing us to obtain true binding affinities (*K*_D_-values) under equilibrium conditions. In-solution measurements are particularly important when working with dynamic and flexible species such as monomeric and αSO. We fluorescently labelled either α-syn monomer or αSO and then added increasing amounts of our mAb. α-syn monomer alone was consistently measured to have an *R*_h_ of 3.2 nm, which is comparable to the previously reported values ranging from 2.7 nm to 4.2 nm (Fig. S2a)^56–58^. Upon addition of antibodies, the *R*_h_ of the complex increases to values from 4.1 nm to 9.0 nm; the low values often reflected incomplete titration (Fig. S2a; compare the complete titration of mAb 17-9D12-A1 with the incomplete one of mAb18-3A5 next to it in the figure). The *R*_h_ of the αSOs was on average 8.4 nm, which is below the previously reported value of 11 nm estimated by size exclusion chromatography with dynamic light scattering (SEC-DLS)^21^, yet comparable to similar measurements by microfluidic diffusional sizing (MDS) (see below and ref. ^59^). The difference may reflect the fact that SEC-DLS was based on light scattering rather than diffusional properties.

Representative examples of titration of mAbs towards mono- and αSO measured by FIDA are shown in Fig. 2bc, where one antibody (03-9H9) shows a marked preference for the αSO while another (17-9D12-A1) binds with equal affinity to both species, although both antibodies show similar IC_50_ values in the competition assay. The highest affinity towards αSOs was around 3-5 nM, shown by 4 mAbs (03-9H9, 14-9E7-A1, 18-9E10-B1 and 19-2C3-F10). 03-9H9 showed the greatest preference for αSOs compared to monomers, with a 559-fold lower *K*_D_ value for binding to the αSO compared to monomeric α-syn. Many other antibodies showed >10-fold increased affinity for αSO over monomeric α-syn (raw FIDA data for all mAbs in Fig. S2a and summarized in Table 2 columns E-G). Interestingly, the commercial antibody MJF14, which has been reported to strongly prefer the aggregated state of α-syn, showed a higher affinity for αSO (0.3 nM, Fig. 2d), but only a 10-fold lower affinity for monomeric and fibrillar α-syn, i.e. a considerably lower preference for αSOs than several of our in-house mAbs. The antibody pSer129 raised against phosphorylated Ser129 showed weak (µM) affinity (Fig. 2e), as would be expected given the absence of phosphorylation in our recombinant α-syn. mAb 06-10H11-B2 showed only weak indications of binding to monomeric α-syn at the highest tested concentrations. However, its affinity for αSO was one of the two lowest we measured (0.8 µM, only marginally better than the 0.9 µM observed for 21-9H11-D11 which also bound monomeric α-syn weakly). Thus, the absence of significant monomer binding should not be taken as evidence for αSO specificity but only for weak binding in general. Relative affinities of mAbs for αSO versus monomeric α-syn are plotted in Fig. 2f.

Several mAbs presented unconventional behavior towards the αSOs. mAb 19-1D2-F9 led to a large complex above the detection limit. In direct contrast, three mAbs (16-9E5, 20-5H12-C10, and 20-9E2-B8) showed the presence of a ~3 nm species corresponding to monomeric α-syn upon titration of the mAbs. We speculated whether this observation could be indicative of either a degree of disintegration of the αSO upon binding or an artifact from interactions with the microfluidic fused silica capillary. To resolve whether the integrity of the αSOs is affected by binding of these three mAbs, we incubated the mAbs individually with the αSO in a 1:10 αSO:mAbs ratio for 20 mins at RT, which reasonably reflects the conditions of binding in the capillaries, and investigated the presence of the mono- and αSOs of α-syn with SDS-PAGE (Fig. S2b). Here, we observed that none of the mAbs affect the integrity of the αSO as seen by both the high intensity of the αSO band and the absence of monomeric α-syn upon incubation with the mAbs. This ruled out αSO disintegration. Another possibility is that the apparent disintegration of αSO observed by the FIDA method with these two mAbs is an artefact caused by agglutination through cross-linking of multiple αSOs via bivalent mAbs into large complexes. However, we saw no direct evidence of large aggregates reaching the detector (which would manifest as spikes). This could suggest that the agglutinated complexes adsorb to the capillary surface. We are unable to investigate this further, but if that indeed is the case, then the observed ~3 nm entity detected during the titration of the three species likely arises from the presence of monomeric constituents within the αSO sample. These monomeric entities are typically in the background of the αSO samples but may assume a dominant role following agglutination and subsequent adsorption of the αSO:mAb complexes.

Overall, we saw no correlation between ELISA-based IC_50_ values and either (i) the antibodies’ affinities to either monomer or αSO, or (ii) the ratio between the two affinities (data not shown). This suggests that the antibody affinities are very dependent on the presentation of the α-syn antigen, i.e. whether immobilized (ELISA) or in solution (FIDA).

**Fig. 3:**
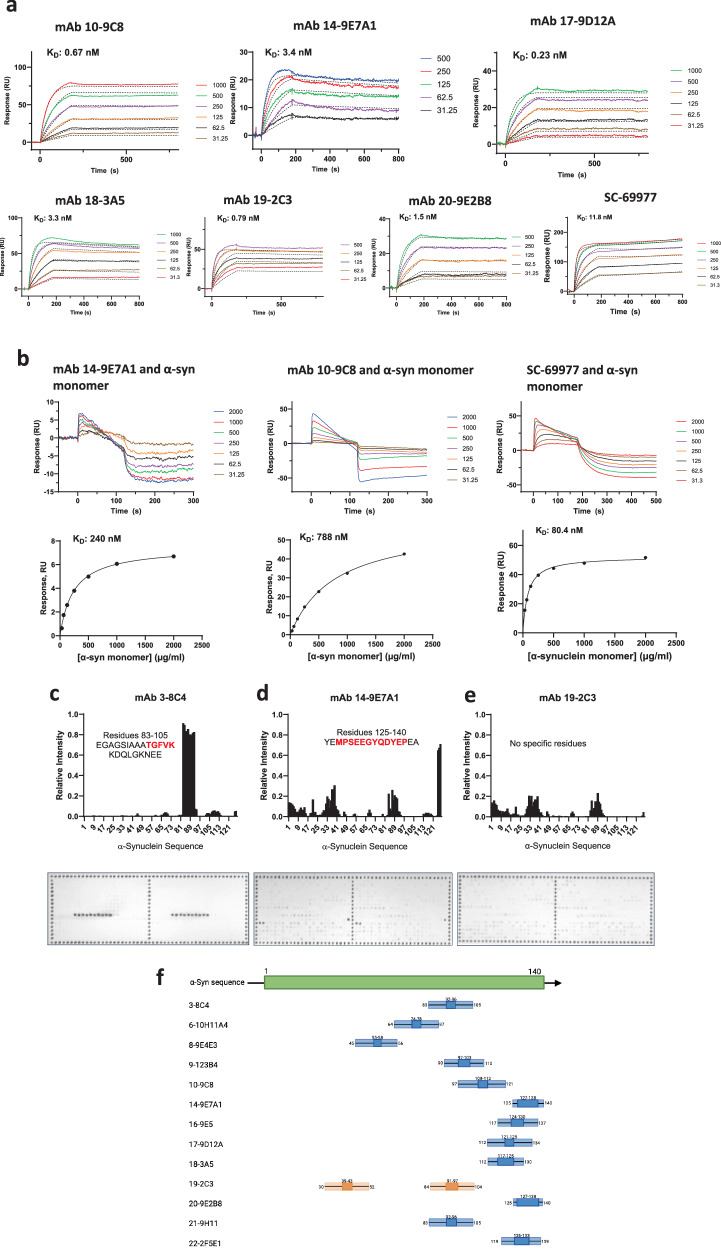
Binding of mAbs to αSOs and α-syn peptides based on different surface techniques. **a** SPR measurements of binding of the indicated concentrations of αSO to immobilized mAbs. *K*_D_ values indicated in all cases. SC-69977 is a commercial mAb (Santa Cruz) raised against monomeric α-syn. **b** Top row: SPR measurements of binding of the indicated concentrations of monomeric α-syn to immobilized mAbs and the commercial anti- α-syn antibody (Santa Cruz). Data did not conform to a simple binding model. Bottom row: steady state response levels versus [monomeric α-syn] fitted to a simple binding isotherm showed very low affinity compared to αSOs (panel **a**). **c**–**e** Microarray-based identification of linear epitopes for mAbs raised against αSOs. Representative data for 3 antibodies shown. Top panels show relative intensity versus the position of the first residue in a given 14-mer peptide in the α-syn sequence (data normalized to intensity in panel for Ab 3-8C4). The bottom panels show scanned images of the peptide arrays (each chip consists of 2 identical arrays). Note that the 127 α-syn peptide sequences only constitute part of the 384 peptides displayed on each array. **f** Graphical display of the binding regions identified for each mAb, with numbers indicating residue positions and the central dark blue rectangle showing the sequence shared by all the peptides in the full blue rectangle. Orange rectangles for mAb 19-2C3 highlight the low intensity of the binding spots for this mAb (cf. graph above), suggesting a structural rather than a linear epitope.

To complement our FIDA data for binding of αSOs and monomeric α-syn to mAbs, we initially attempted to use biolayer interferometry, but this yielded unsatisfactory results (Fig. S2c and associated text). Instead, we turned to surface plasmon resonance (SPR) on a Biacore 3000a instrument, where immobilized antibodies were exposed to αSOs or monomeric α-syn. The resulting binding curves yielded αSO *K*_D_-values in the low nM values for all mAbs (Fig. 3a, summarized in Table 2 column E). Interestingly, the measured *K*_D_-values were 5-50-fold lower than for FIDA measurements except for 14-9E7A1 where identical values were obtained by the two methods. mAb 18-3A5 had the lowest affinity of the 6 mAbs in FIDA assays, but showed a significantly better binding by SPR (76 versus 3.3 nM). The mAb 20-9E2B8, which had shown unusual behavior in FIDA, yielded a respectable *K*_D_-value of 1.5 nM (Fig. 3a), indicating that the FIDA data for this mAb was an anomaly. Monomeric α-syn showed unusual binding signals, whose kinetics could not be fitted to a simple 1:1 binding model (Fig. 3b). Closer inspection suggests that the monomeric α-syn speeds of dissociation of mAb from the chip surface, leading to a sloping baseline and negative RFU values at high protein concentration, i.e. monomeric α-syn may compete with protein G for the same binding site on the mAb (outside the CDR). Plotting steady-state responses versus α-syn concentration leads to binding isotherms with *K*_D_-values close to 1 µM; however, the maximal responses estimated from these fits are much lower than the theoretically expected values. All this indicates that the α-syn interaction is not a classical antigen-antibody interaction and strongly reinforces the observation that these mAbs preferentially interact with aggregated versions of α-syn such as αSOs. The commercial antibody SC-69977 (Santa Cruz) showed a reasonable affinity for αSO (11.8 nM, Fig. 3a, which is however significantly weaker than the 6 mAbs) but an almost comparable affinity for monomeric α-syn (80 nM based on steady-state response, Fig. 3b). This makes good sense, given that the antibody was raised against monomeric α-syn.

### Assays using truncated α-syn show high diversity in recognition of the C-terminal part of α-syn

To identify possible linear epitopes, we analyzed the extent to which the mAbs were able to recognize monomeric α-syn truncated at the C-terminus to different extents. We carried out indirect ELISA assays using immobilized monomeric α-syn of lengths 1-100 to 1-140 in steps of 10 residues. As expected, the antibodies obtained from immunization with cVLPs displaying α-syn of lengths 1-100 and 1-120 (mAbs from mice 1-8) were able to recognize α-syn constructs of the corresponding lengths; similarly, mAbs from mouse 10 (immunized with αSO 1-125) recognized shorter constructs (in some cases down to 1-100). The remaining antibodies had all been obtained from immunization with full-length α-syn and varied considerably in their requirement for the C-terminal tail with no obvious pattern (Table 2 column H). For example, immunization with unmodified full-length αSOs led to a set of mAbs recognizing the full spectrum of α-syn lengths from 1-100 to 1-140. Overall, six mAbs recognized down to α-syn length 1-100, five down to 1-110, three each to 1-120 and 1-130 while seven required full-length 1-140. This underlines once again the diversity in antibody affinities and epitopes.

To complement these studies, we turned to peptide arrays displaying 14-residue peptides spanning the α-syn sequence in steps of 1 residue (i.e. residues 1-14, 2-15, 3-16… 127-140). The different mAbs were each presented to a peptide array and their extent of binding quantified using a secondary antibody, followed by scanning of the array. Representative data for 3 mAbs in Fig. 3c-e revealed binding to different sites of the sequence and with varying itensity. mAb 3-8C4 showed a very strong affinity for amino acids 83-105 of the sequence, while mAb 14-9E7A1 showed slightly less strong but still very specific binding at 125-140 which corresponds to the very end of α-syn. In contrast, mAb 19-2C3 (as well as mAbs 18-9E10B1 and 19-1D2, data not shown) showed little or no specific binding to the peptides, suggesting rather that it recognized a structural epitope which was not displayed in monomeric α-syn (and thus not in the peptides). The binding of 10 other mAbs are shown in Fig. S2d. As summarized in Fig. 3f, most of the 12 tested mAbs which showed clear binding to the α-syn sequence, tended to bind to the C-terminal end of the sequence, with only two mAbs (6-10H11A4 and 8-9E4E3, both of which were able to bind to truncated α-syn 1-100 in Table 1H) binding towards the middle of the sequence. For those mAbs where we were able to obtain binding data both to truncated α-syn and to the peptide array, there was excellent agreement between the two complementary approaches, confirming the general conclusions in Fig. 3d.

### Microfluidic diffusional sizing shows binding to α-syn fibrils

To examine the affinity of our mAbs to fibrils, we turned to microfluidic diffusional sizing (MDS). MDS is similar in principle to FIDA but differs in that it measures diffusion (and thus *R*_h_) based on the transfer of fluorescently labelled mAbs from a central laminar flow phase to two surrounding phases^52,59^ rather than by its diffusion within one phase. As fibrils are highly heterogeneous, an exact size estimate of the bound antibody was difficult to estimate. Nevertheless, using the apparent ratio of free antibody to bound antibody, we could determine *K*_D_ values as well as the number of α-syn monomers making up an individual binding site for each antibody (binding plot for 20-2E2-B8 shown in Fig. 2g; data for all five mAbs shown in Fig. S3a and summarized in Table 3 where they are compared with corresponding values for αSOs and monomeric α-syn). Three mAbs (10-9C8-C1, 17-9D12-A1, and 19-2C3-F10) showed a higher preference for αSOs compared to fibrils, with a 2–20-fold lower *K*_D_^αSO^ value compared to *K*_D_^fibril^ (note that fibril binding values for 17-9D12-A1 have large errors due to a restraint in the fitting). mAb 18-3A5-H2 shows significantly higher affinity for fibrillated α-syn compared to αSO, while 20-9E2-B8 did not give rise to reliable FIDA data (see previous section) and is therefore difficult to compare. For comparison, MJF14 showed an almost 100-fold lower affinity for fibrils than αSO (36 vs. 0.42 nM), demonstrating the strongest preference for oligomers over fibrils of the 6 mAbs in Table 3. The second best is our mAb 10-9C8-C1, which has a ca. 14-fold αSO-fibril preference; however, it shows significantly weaker binding to monomeric α-syn than does MJF14 (97 vs. 3.92 nM).

**Table 3:** Data for binding of selected mAbs to α-syn in the fibrillated, oligomeric and monomeric state

mAb	*K*_d_^fibril^ (nM)^a^	n^b^	R^2^	*K*_d_^oligomer^ (nM)^c^	*K*_d_^monomer^ (nM)^c^
10-9C8-C1	82.6 ± 105.2	2.33 ± 1.48	0.8753	6.1	97
17-9D12-A1	202.8 ± 238.3	1 ^d^	0.8965	12	9.4
18-3A5-H2	3.82 ± 13.4	4.02 ± 0.93	0.7875	76	5767
19-2C3-F10	9.83 ± 17.1	1.845 ± 0.48	0.8473	4.2	312
20-9E2-B8	18.5 ± 15.9	5.58 ± 1.00	0.9509	(monomerizes)	3800
MJF14	36.3 ± 16.7	11 ± 2	0.9745	0.42	3.92

^a^Determined by microfluidic diffusional sizing.

^b^α-syn monomers/binding site.

^c^Determined by FIDA.

^d^The model balances the concentration of binding sites available to the antibody with *K*_d_^fibril^. Since the two values could compensate for each other (leading to a misleadingly high *K*_d_^fibril^ and a very low *n* and vice versa), *n* is restrained to be at least 1 monomer per binding site. The fit with 17-9D12-A1 was limited to this value.

### ThT fibrillation data show only modest effects on aggregation kinetics

**Fig. 4:**
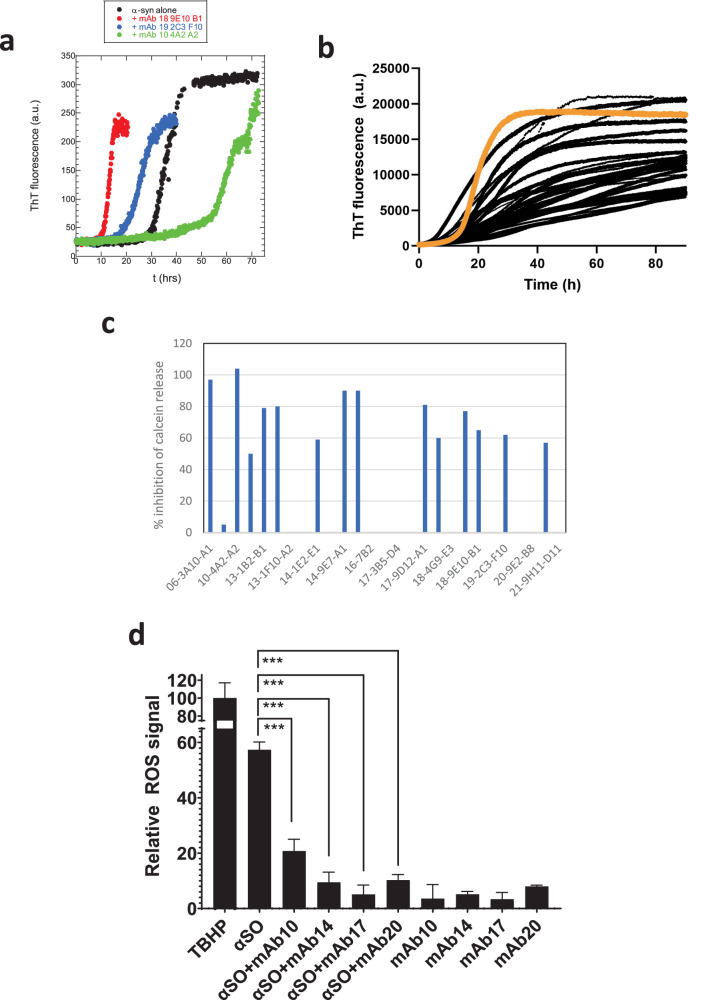
Effect of mAbs on fibrillation of monomeric α-syn. **a** Thioflavin T fluorescence time profiles of α-syn aggregation alone and in the presence of three different mAbs, two of whom accelerate fibrillation while one decelerates it. **b** Seeding experiments in which monomeric α-syn is fibrillated in the presence of 1% fibrillated α-syn, either without (orange curve) or with (black curves) different mAbs. Data for individual mAbs summarized in Table 2. **c** Ability of different mAbs to inhibit αSO permeabilization of calcein-filled DOPG vesicles. mAbs with 0% inhibition values did not decrease calcein release levels to a significant extent or led to calcein release on their own. **d** Generation of ROS by 40 µg/ml αSO in SHSY5Y cells with and without preincubation of αSO with 150 µg/ml of different mAbs. Errors are standard errors of the mean from triplicate measurements. ***: *p* < 0.001.

To evaluate the impact of our mAbs on the fibrillation of α-syn, we incubated 0.25 mg/mL monomeric α-syn with twice the mass concentration of mAb (0.5 mg/mL) and monitored the accumulation of fibrils using the fluorescent amyloid-binding probe ThT. Fibrillation was evaluated as the time required to reach t_½_ (mid-way time to maximum fibrillation). On its own, α-syn fibrillates with a t_½_ of 36 ± 5 h under these conditions. Of the 28 mAbs we tested (summarized in Table 2 columns I & J), 11 had no significant effect on α-syn fibrillation (t_½_ values 30–40 h within error), while 12 accelerated fibrillation to some extent (t_½_ values down to 13 ± 1 h) and five (antibodies 10-4A2-A2, 13-1F10-A2, 14-2B8-G1, 14-9E7-A1, 18-4G9-E3) slowed down fibrillation (t_½_ values up to 61 ± 12 h). Some typical runs are shown in Fig. 4a. When t½ values are normalized to that observed in the absence of mAb (36 h), the average t½ value is around 0.9 ± 0.36. A slightly greater effect is seen in seeding experiments (Table 2 column K**)** where the bottle neck of nucleation is largely bypassed by the addition of a small amount of preformed fibrils; a deceleration in fibrillation could be expected from mAbs binding either to the growing ends of fibrils or monomers. Here the relative half-life of fibrillation is on average more than doubled (average t_½_^relative^ 2.8 ± 1.1) and the ThT fluorescence level is also somewhat reduced (Fig. 4b), with an average relative level of 0.62 ± 0.19 (normalized to ThT levels in the absence of mAb). However, the modest spread in effects suggests that the antibodies do not have strong affinity for species that are critical for fibrillation, e.g. monomers, nuclei, or growing ends of fibrils.

We spun down the contents of each fibrillation well and ran the supernatant on SDS-PAGE to determine the amount of α-syn left in solution and thus not incorporated into insoluble fibrils (Fig. S3b). In all cases, most of the α-syn was incorporated into fibrils, but several mAbs, in particular 18-4-G9, 18-7-B9, 19-1D2 and 20-9E2, retained a substantial amount of α-syn in solution (according to densitometric analysis with ImageJ, this corresponded to 0.15–0.2 mg/ml, i.e. 30-40% of the initial amount of α-syn) and also showed the lowest levels of ThT fluorescence. In other words, the two sets of observations are internally consistent: the antibodies that most reduce the level of fibrillation (according to ThT fluorescence) also lead to the highest amount of soluble α-syn. Nevertheless, the low overall effects are consistent with the low FIDA affinities for the monomer and the fact that the αSO is an off-pathway species relative to the major fibrillation pathway of α-syn.

We also took samples from a solution of 1 mg/ml α-syn monomers under fibrillating conditions at regular intervals over a 48-h period and used 5-20 ng samples in dot blot assays to test for changes in the overall level of binding of a number of different mAbs to the α-syn population as aggregation progresses. However, we were not able to establish any definite pattern in the variation of binding with time, which we attribute to the very high levels of monomeric α-syn in the solution (data not shown).

### Calcein release studies

As a simple in vitro evaluation of these mAbs, we tested their ability to block a critical function of the αSO, namely its ability to permeabilize vesicles. We used a simple release assay, in which anionic vesicles consisting of DOPG lipids were filled with the fluorophore calcein at self-quenching concentrations (ca. 50 mM). Release of calcein to the calcein-free exterior leads to a ~10-fold increase in fluorescence. We have previously shown this simple assay to correlate well with more direct cell toxicity assays while yielding robust and readily available results^49^. We used an αSO concentration of 5 µg/ml (0.35 µM in monomer α-syn units) which led to ~50% of maximal calcein release (normalized to complete lysis with the detergent Triton X-100, Fig. S4), and preincubated the αSO with 100 µg/ml (0.67 µM) mAb (corresponding to 60 mAb per αSO or 2 mAb per α-syn monomer subunit) prior to exposure to vesicles. Again, there was a very large spread in the extent to which mAbs blocked αSO activity, with seven mAbs showing ≥80% blocking of release, seven showing 40-80% blocking and the remainder having no discernible effect (Fig. 4c, summarized in Table 2 column L).

To complement these assays with a more direct cellular assay, we measured the formation of reactive oxygen species in neuroblastoma SHSY5Y cells, using the formation of the highly fluorescent compound dichlorofluorescein. On its own. αSO generated a significant amount of ROS signal (~60% compared to the positive control tert-butyl hydroperoxide, Fig. 4d), but this decreased to a very significant extent when αSO was preincubated with a selection of different mAbs. This confirmed the protective properties of our anti- αSO antibodies.

At the biophysical level, we conclude that the 30 mAbs show a broad range of relative and absolute affinities for the different α-syn species (monomer, αSO, and fibril), a modest effect on actual fibrillation (indicating a low binding to monomeric α-syn, fibrillation nuclei or the growing ends of fibrils) and in several cases a very promising effect on calcein release. The modest fibrillation effects that are seen can mechanistically be explained by preferential binding to certain species along the aggregation pathway. For example, acceleration of α-syn aggregation (Fig. 4a) could stem from acceleration of the initial nucleation of α-syn, e.g. by stabilization of the fibrillation nucleus by that particular mAb, while shortening of the lag phase and subsequent (slight) inhibition of the elongation phase (Fig. 4b) could indicate the same stabilization of the nucleus but inhibition of secondary processes such as fragmentation or lateral nucleation. The relatively strong binding to fibrils for some of the antibodies (Table 3) did not significantly impact the fibrillation process, indicating that the antibodies do not interfere strongly with fibril growth, possibly due to lateral association to fibrils. While none of the mAbs had an absolute preference for αSOs compared to the other α-syn species consistent with the observations by Lashuel and coworkers^43^, many showed a > 20-fold increased preference for αSOs over monomeric α-syn. The commercial antibody MJF14 only showed a 10-fold preference for αSOs though a higher overall binding affinity. This contrasts with previous reports on MJF14 binding specificity, which are however based on ELISA^60^ or SPR^43^ (in the latter case using immobilized antibodies) and may therefore not be directly comparable. The SPR studies also showed multi-exponential binding and release steps in some cases^43^, implying the existence of multiple populations of both monomeric and αSO (or perhaps of the antibodies). Interestingly, MJF14’s SPR-derived *K*_D_ values were 0.76 nM and 2.8 pM; the first value of 0.76 nM corresponds quite well to the value of 0.3 nM we determine by FIDA (which however does not detect the high-affinity binding). This suggests that the 2.8 pM value may be a result of the immobilization of the antibody. Similarly, the other high-affinity mAb reported in^43^ showed multiple affinities of 249 nM and 2.14 pM; it is possible that the latter value also derives from immobilization. Taken at face value, these results emphasize how sensitive recognition of α-syn species is to the way in which they are presented to antibodies (and vice versa) and suggest that antibody affinities should be measured in solution to avoid such artifacts.

### mAbs recognize distinct human α-syn pathologies in the brain of the α-syn viral vector PD rat model

We now turn to an evaluation of how a subset of these mAbs perform in recognizing α-syn pathology in brain tissue, bearing in mind that this involves fixated tissue; the process of fixation can also be expected to affect the conformations of the different α-syn species. For these experiments, we discontinued six antibodies that showed very weak binding to the αSO (03-8C4, 06-10H11-B2, 16-3B1, 16-7B2, 21-9H11-D11) or were difficult to produce (17-3B5-D4). Of the remaining 23, we chose 16 spanning as diverse a range of binding profiles as possible.

**Fig. 5:**
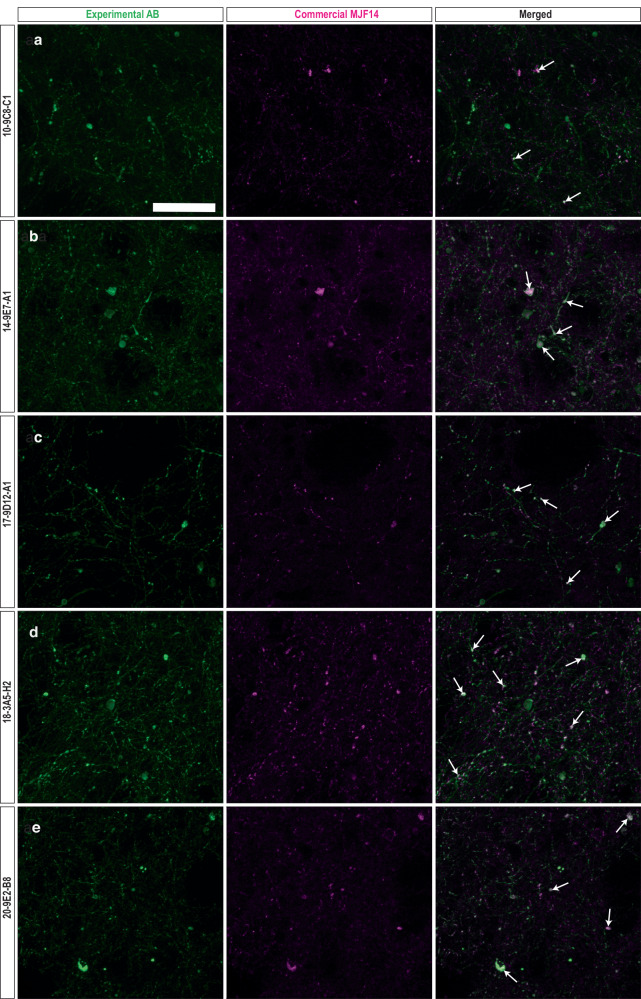
Co-staining of striatal sections from rats injected with rAAV-human α-syn with the mAbs and an anti-aggregated α-syn commercial antibody. Photos of representative immunofluorescence staining with the experimental mAbs (left) and the commercial anti-aggregated α-syn **(**MJF14, middle) and the merged image (right). Co-staining of MJF14 with mAbs (**a**) 10-9C8-C1, (**b**) 14-9E7-A1, (**c**) 17-9D12-A1, (**d**)18-3A5-H2, (**e**) 20-9E2-B8. Images represent areas with values close to the average obtained from triplicate staining that are shown in Table 4. Green: mAbs. Magenta: MJF14. White: Merged/overlap. Scalebar = 50 µm, applied to all photos. White arrows: examples of overlap.

**Fig. 6:**
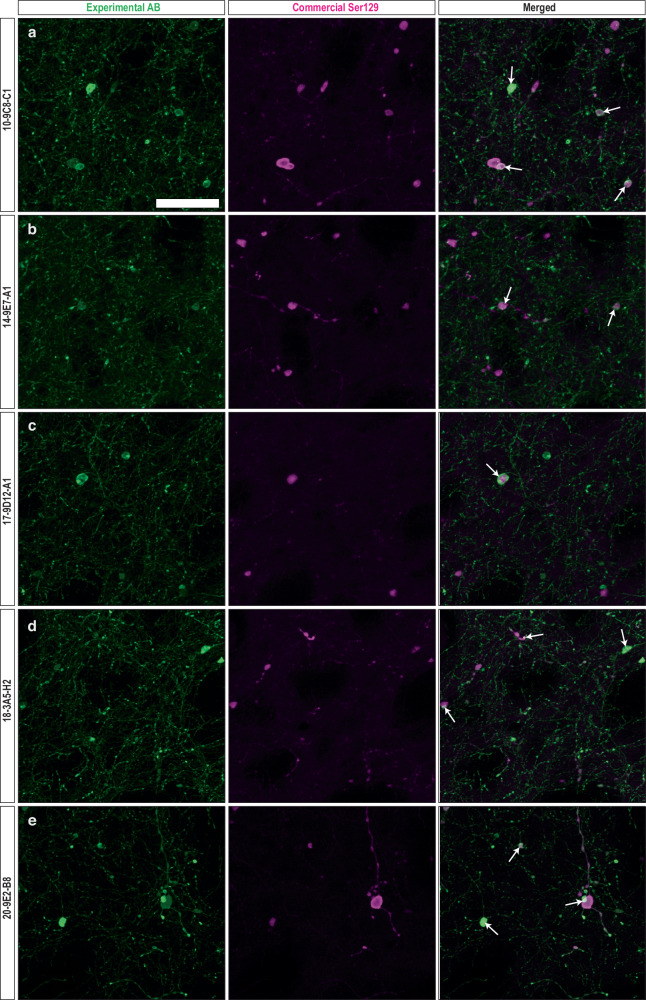
Co-staining of striatal sections from rats injected with rAAV-human α-syn with the mAbs and an anti-phosphorylated Ser129 α-syn commercial antibody. Photos of representative immunofluorescence staining with the experimental mAbs (left) and the commercial anti-Phosphorylated Ser129 α-syn (Ser129, middle) and the merged image (right). Co-staining of Ser129 with (**a**) 10-9C8-C1, (**b**) 14-9E7-A1, (**c**) 17-9D12-A1, (**d**)18-3A5-H2, (**e**) 20-9E2-B8. Images represent areas with values close to the average obtained from triplicate staining that are shown in Table 4. Green: mAbs. Magenta: Ser129. White: Merged/overlap. Scalebar = 50 µm, applied to all photos. White arrows: examples of overlap.

To investigate and compare the ability of our mAbs to recognize human α-syn pathology, brain sections from a rat PD model were co-stained with 16 different mAbs together with two well-characterized commercially available antibodies recognizing: phosphorylated Ser129 α-syn (pSer129) or (MJF14) aggregated α-syn (both fibrils and αSOs prepared either unmodified or in the presence of dopamine or HNE)^43^. As shown previously in Fig. 2d–e, MJF14 recognizes both monomeric and αSO with only a 10-fold preference for the αSO, while pSer129 does not recognize unphosphorylated α-syn. As the animal model of PD-like human α-syn pathology, we used rats injected with rAAV h-α-syn into the ventral midbrain; a model that shows overt human α-syn expression and aggregates in the nigrostriatal pathway^61^. Immunofluorescence was performed on straitum (STR) sections using mAb concentrations based on indirect ELISA assays (i.e. the minimal mAb concentration giving an ELISA read-out of ca. 1.5 OD_450nm_ at 1.0 µg/ml αSO) (Fig. 1a). We performed a systematic analysis of the immunostaining observed in the dorsal-lateral area of the STR, as this area shows a significant number of axons containing pathological accumulations of human α-syn in this PD model^61^. To evaluate and compare staining for each mAb with the two commercial antibodies, we quantified; (1) the area covered by each antibody to see how much α-syn pathology they could reveal and (2) the area co-stained and Pearson co-localization coefficient and (3) the percentage of co-stained area to evaluate the overlap of the staining observed between the mAbs and the commercial ones (Table 4). Positive staining in the axonal fibers and round inclusions, i.e. α-syn pathology, was found in the ipsilateral STR with all mAbs. However, the area of staining and co-localization with the commercial antibodies varied. Most mAbs showed varying levels of co-localization with MJF14, suggesting that they were able to recognize aggregated α-syn (Table 4, Fig. 5). Lower levels of co-localization were found with pSer129, thus, not all mAbs recognized phosphorylated Ser129 α-syn (Table 4, Fig. 6).

**Table 4:**
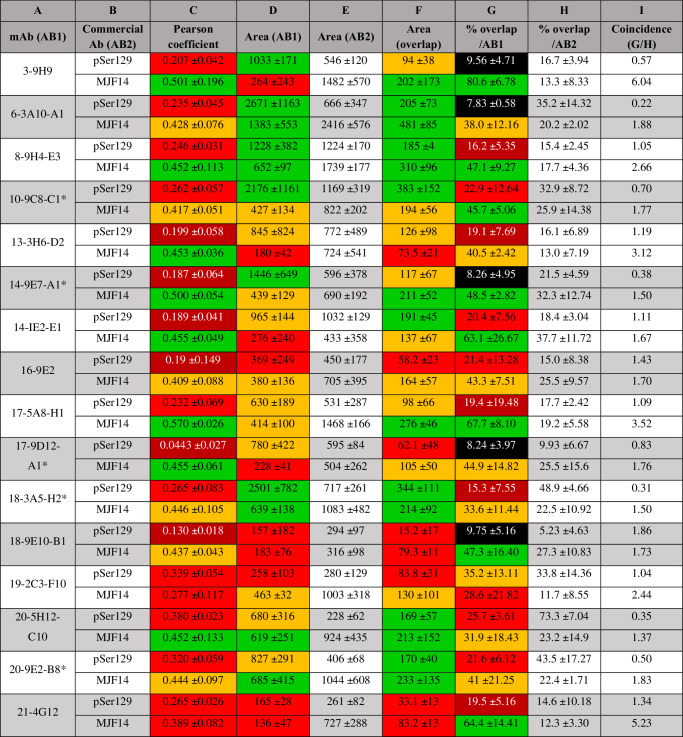
Quantification of the binding of experimentally raised antibodies (mAbs) to rat striatal sections compared to commercial anti-α-syn antibodies

Data provided quantifies co-localization, area covered and %overlap.

^a^ * denotes mAbs selected for *postmortem* human studies. Values are given as mean ± SD. Values in columns C-D and F-G are color-coded according to the following 3-5 ranges described in Materials and Methods using the following color-bar (where green is the highest and black the lowest).

#### Area covered by staining with the mAbs (Table 4, columns D and E)

The top four mAbs showing the biggest area stained in (i) the pSer129/mAbs co-stainings were 6-3A10-A1, 10-9C8-C1, 14-9E7-A1, and 18-3A5-H2, and in (ii) the MJF14/mAbs co-stainings were 6-3A10-A1, 8-9H4-E3, 18-3A5-H2, and 20-9E2-B8. We observed that the area covered by staining for most of the mAbs was higher when analyzing pSer129/mAbs stained sections (> 600) vs. that observed in sections co-stained with MJF14/mAbs (< 600) (Table 4, column D, top and bottom number respectively). This could be due to a possible epitope competition between the MJF14 antibody and our mAbs. Moreover, areas stained by the MJF14 (and thus showing aggregated α-syn) (Table 4, column E, bottom number), were higher than those covered by most of our mAbs (Table 4, column D, bottom number), which could be due to the higher affinity of the MJF14 compared to our mAbs. Most of our mAbs stained a bigger area than the one stained by the pSer129 (Table 4, column D vs. E, top numbers) suggesting that our mAbs also recognize non-phosphorylated α-syn, or that not all αSOs in this rat model was phosphorylated. mAbs were scored from low to high according to the areas covered by our mAbs staining within each co-staining pSer129/mAb or MJF14/mAb (see methods). According to this, mAbs 6-3A10-A1, 8-9H4-E3, and 18-3A5-H2 showed high areas of staining in both co-stainings, while mAbs 18-9E10-B1 and 21-4G12-C12 showed low areas for both. Notably, mAb 3 9H9 showed a high area with pSer129/mAb (1032.9) but low with MJF14/mAb, (263.5), while the rest scored combinations between medium to high or low to medium in both co-stainings. In conclusion, our mAbs detect some forms of pathologically accumulated α-syn although to different degrees.

#### Area co-stained by the mAbs and the commercial antibodies (Table 4, columns C and F)

We next scored the mAbs according to the areas overlapped within each co-staining (see methods) (Table 4, column F). The areas of overlap in the MJF14/mAb co-staining were higher (>200 in 8 of the 16 mAbs) than those in the pSer129/mAb staining (>200 only in 3 of the 16 mAbs), suggesting a higher similarity between the epitopes or α-syn strain recognized by our mAbs and the MJF14 vs. pSer129. mAbs 6-3A10-A1, 8-9H4-E3, 18-3A5-H2, 20-5H12-C10, and 20-9E2-B8 showed a high area of overlap in both co-stainings, while mAbs 18-9E10-B1 and 21-4G12-C12 showed low areas for both. The rest mAbs showed medium-to-high or low-to-medium in both co-stainings. The higher areas of overlap in the MJF14/mAbs co-staining were confirmed by their higher Pearson co-localization coefficient (>0.4 in 14 of 16 mAbs) vs. that seen of the pSer129/mAbs co-staining (<0.4 in all mAbs) (Table 4, column C). Notably, only mAb 19-2C3-F10 showed a higher Pearson coefficient for pSer129 (0.339) than for MJF14 (0.277). The mAbs were also scored according to the Pearson co-localization (see methods), and no mAbs scored high (Pearson coef. >0.450) for both, however, mAbs 19-2C3-F10 and 21-4G12-C12 showed low scores (0.200-0.400) for both. The mAbs 13-3H6-D2, 14-9E7-A1, 14-1E2-E1, and 17-9D12-A1, showed high scores in Pearson coefficient for the MJF14/mAbs (>0.450) co-staining, but very low in the pSer129/mAbs (<0.200), thus high co-localization with the MJF14 and low with the pSer129.

#### Percentage of the area covered (Table 4, columns G and H)

As the Pearson algorithm does not consider the area covered by all staining, but only the staining within the intersection, we also analyzed the percentage of the area covered by our mAbs also covered by the commercial antibodies (Table 4, column G). This analysis again showed a higher percentage of area co-stained with the MJF14 (12 of the 16 mAbs >40%) vs. the pSer129 (all mAbs <40%), confirming that the α-syn forms recognized by mAbs share similarities or co-localize with those recognized by the MJF14. mAbs 17-5A8-H1, 14-1E2-E1, and 21-4G12-C12 showed approximately 60% overlap with MJF14, indicating high similarity, with mAb 3-9H9 showing the highest (80.6%). However, when the percentage of overlap was calculated over the MFJ14 area (Table 4, column H bottom), mAb 3-9H9 only stained 13% thus, MJF14 recognized α-syn forms not seen by mAb 3-9H9. Interestingly mAbs 19-2C3-F10 and 20-5H12-C10, which had the lowest percentage of the area co-stained by MFJ14 (28% and 31% respectively), conversely showed the highest percentage of the area co-stained by pSer129 (35% and 25% respectively) (Table 4, column G). Of these two, when the percentage of overlap was calculated over the pSer129 area (Table 4, column H top), 20-5H12-C10 stained 73%. Thus, the epitopes recognized by 20-5H12-C10 and the pSer129 are very similar or often colocalized. Corroborating the Pearson coefficient values, mAb 19-2C3-F10 colocalized better with the pSer129 as it showed always higher percentage of area also stained with pSer129 than for MJF14 irrespective of how it was calculated (Table 4, column G and H). The mAbs 10-9C8-C1, 14-1E2-E1, 16-9E5, and 20-9E2-B8 also showed over 20% of the area also co-stained with pSer129 (Table 4, column G). However, mAbs 6-3A10-A1 and 18-3A5-H2, which were also in the group with the lowest percentage (<40) of area co-stained with MJF14 also showed low-very low percentage of area co-stained with pSer129 (7.8% and 15% respectively), suggesting a unique epitope recognition.

#### Coincidence number (Table 4, column I)

Finally, we calculated the coincidence number by dividing the percentage of areas co-stained by in-house and commercial antibodies (i.e. dividing values in column G by column H). A high coincidence number indicated that the commercial antibodies detect numerous α-syn species that our mAbs do not detect, while a low number indicated the opposite. mAbs 6-3A10-A1, 10-9C8-C1, 14-9E7-A1, 17-9D12-A1, 18-3A5-H2, 20-5H12-C10 and 20-9E2-B8 have the lowest coincidence number for both commercial Abs (<1 vs. pSer129 and 1-2 vs MJF14), suggesting they identify unique α-syn forms not recognize by the commercial antibodies. Furthermore, although confocal images showed that most of our mAbs stained axonal swellings, which were also positive for pSer129. mAbs 10-9C8-C1, 14-9E7-A1, and 20-9E2-B8, stained axonal swellings, which only partially co-localized with pSer129 (Table 4, column C and G, Fig. 6a, b and e), further supporting a unique recognition pattern of these three mAbs in these pathological formations.

Based on these observations in the immunofluorescence experiments and the initial ELISA curves, we chose the following five mAbs for further analysis: 10-9C8-C1, 14-9E7-A1, 17-9D12-A1, 18-3A5-H2, and 20-9E2-B8, all of whom showed low coincidence number for both commercial Abs (Table 4, column I). mAb 18-3A5-H2 showed a high area of staining but a low percentage of area co-stained with both commercial antibodies and a low-medium Pearson coefficient in co-stainings with pSer129 and MJF14. mAbs 10-9C8-C1 and 20-9E2-B8 showed mid-to high/very high percentage of area co-stained with both commercial antibodies, low-mid Pearson coefficient in pSer129 and MJF14 co-stainings, and medium-high area of staining, but with 10-9C8-C1 having higher area in the pSer129 staining vs MJF14, whereas this was the opposite for 20-9E2-B8 (Table 4, column G, C and D, respectively). Finally, mAbs 14-9E7-A1 and 17-9D12-A1 had a high percentage of area co-stained with MJF14 but low with pSer129, accordingly they showed a high Pearson coefficient with MJF14 and low with pSer129, but medium-high area of staining for 14-9E7-A1, while it was low-medium for 17-9D12-A1 (Table 4, Fig. 6).

#### The mAbs recognize rat α-syn in areas of high expression or if pathologically accumulated

As an additional confirmation of these 5 mAbs’ ability to recognize α-syn species in complex mixtures, we carried out indirect ELISA assays on lysates of brains of transgenic human A53T α-syn (line M83) mice. Lysates were diluted up to 160-fold and used to coat 96-well plates, after which they were blocked and exposed to each of the 5 selected mAbs, using the MJF14 mAb as positive control. All 5 mAbs were able to recognize signal in the brain lysate, although to a slightly lower extent than the MJF14 mAb (Fig. S3c, left panel). This is consistent with MJF14’s ability to recognize immobilized versions of both monomeric α-syn and αSOs to a slightly greater extent than these antibodies (Fig. S3c, middle and right panels).

While staining rat brain sections, we noticed that some of our mAbs showed staining in areas with high expression of endogenous rat α-syn, such as the hippocampus (Fig. S5, bottom row), suggesting that they could recognize monomeric rat α-syn to some extent. The competitive ELISA assay showed a relatively low degree of binding to monomeric human α-syn (Table 2), with an average IC_50_ value of 310 µg/ml. To evaluate how much of the staining observed with the five selected mAbs was due to monomeric α-syn, we performed pre-incubation of the five selected mAbs, and the MJF14 antibody with monomeric human α-syn prior to immunohistochemical staining of the brain sections from the rAAV-α-syn injected rats. As expected, when no pre-incubation was used, all five mAbs stained fibers and inclusions in the ipsilateral STR of the rats (Fig. S5, top row) that was affected by pre-incubation for all mAbs except for mAb 10-9C8-C1 (Fig. S5a-ai, g-gi and m-mi). Staining was decreased after pre-incubation with mAbs 14-9E7-A1 (Fig. S5b-bi, h-hi and n-ni) and 20-9E2-B8 (Fig. S5e-ei. k-ki and q-qi). In contrast, staining became sharper and more intense for mAbs 17-9D12-A1 (Fig. S5c-ci, i-ii and o-oi) and 18-3A5-H2 (Fig. S5d-di, j-ji and p-pi). Non-pre-incubated mAbs 18-3A5-H2 and 20-9E2-B8 also showed fibers stained in ipsilateral cortex (CTX) that became sharper upon pre-incubation (Fig. S5 j-ji and k-ki). However, the (apparent background) cellular staining in CTX stained by mAb 17-9D12-A1 decreased after pre-incubation (Fig. S5i). The mAbs 10-9C8-C1, 14-9E7-A1, and 20-9E2-B8 showed positive punctate staining in the hippocampus (Fig. S5m-mi, n-ni, and q-qi), which resembled the endogenous one, that was not avoid by pre-incubation although it was slightly decreased with the mAb 20-9E2-B8 (Fig. S5qi). With or without pre-incubation, the MJF14 showed fiber staining in the STR (Fig. S5f-fi) but not in the CTX (Fig. S5l-li). However, non-pre-incubated MJF14 stained punctate structures in the hippocampus, which was significantly decreased after pre-incubation (Fig. S5r-ri). Therefore, some of our mAbs could recognize non-monomeric rat α-syn present in hippocampus (Fig. S5).

Based on these observations, we sought to investigate whether mAbs 14-9E7-A1 and 20-9E2-B8 (mAbs showing staining in the hippocampus) as well as 18-3A5-H2 (which showed no staining in the hippocampus) could recognize pathologically aggregated rat α-syn. To do so, we used brain sections from rats injected with murine α-syn PFF into the STR, which leads to pathological aggregation of the endogenous α-syn in different brain areas, including the prefrontal cortex (PFCTX)^62^. All three mAbs 14-9E7-A1, 18-3A5-H2 and 20-9E2-B8 stained structures in the PFCTX of PFF injected rats in a similar fashion as MJF14. However, the quantity and intensity varied: mAb 20-9E2-B8 showed positive staining resembling that seen with MJF14, while mAbs 14-9E7-A1 and 18-3A5-H2 show lower staining levels than MJF14 **(**Fig. S6**)**. Overall, this suggests that the mAbs recognize aggregated rat α-syn as well as human aggregated α-syn.

### The mAbs recognize different pathological structures in different synucleinopathies

**Fig. 7:**
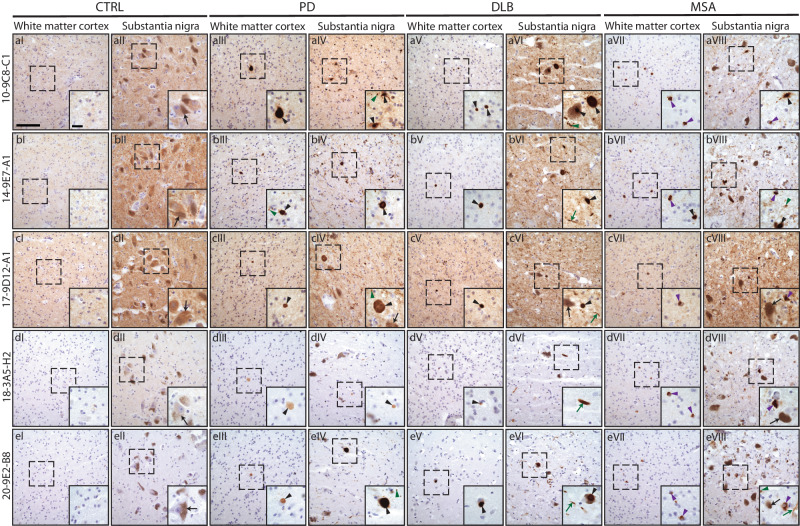
Pilot study on post-mortem brain tissue from PD, DLB and MSA patients. Rows show representative photos from tissue stained with each respective mAbs used (**a**: 10-9C8-C1, **b**: 14-9E7-A1, **c**: 17-9D12-A1, **d**: 18-3A5-H2, **e**: 20-9E2-B8). Columns indicate the patient type and the respective anatomical area stained (I-II: CTRL, III-IV: PD patient, V-VI, DLB patient, VII-VIII: MSA patient; for each pair, the first section is from CTX and the second from SN). Images are taken using a 20x objective (Scalebar: 50 µm). Dashed squares indicate the area taken with the 63x objective (Scalebar: 10 µm). Black arrows: pigmented neurons. Black arrowhead: Lewy Bodies and extracellular inclusions. Green arrow: Lewy neurites. Green arrowhead: neuropils. Grey arrowhead: Glial cytoplasmic inclusions.

The five selected mAbs were used to immunostain *post-mortem* human brain sections from substantia nigra (SN) and subcortical white matter in the frontal cortex (WM CTX) from patients with PD, DLB, MSA, and controls (CTRL) (Supplementary Table S1–5). The number of α-syn pathological structures in each of the two areas were quantified in a region of interest and scored on a 4-point scale given according to the following: 0 structures: -, 1–25: +, 25–50: ++, >50: +++. The structures included were: LBs or LNs, extracellular inclusion bodies, neuropil “grains” and GCIs (Table 5, and Fig. 7 and Fig. S7). As a positive control, we used a commercial antibody (GeneTex), which recognizes human α-syn (both monomeric and aggregated) on the putamen of an MSA patient. All mAbs detected pathology within all patient brains tested (Tables S1–5 and Fig. 7), and also incidental LB in the SN of one of the four controls, which however had no overt loss of pigmented neurons (Tables S1–5, row three). The intensity and quantity of pathological staining varied among the mAbs and the different synucleinopathies (Table 5). In the PD brains, the pathology scores were generally lower in the WM CTX than the SN (Table 5 and Fig. 7). This could be due to the pathological disease stage of these patients, as the cortex is involved later according to Braak stages^63^.

**Table 5:** Overview of quantified structures in *postmortem* brains from synucleinopathies using the different mAbs

Group	Antibody	Area	Pigmented neurons	Extracellular inclusions	LBs	LNs	Neuropil	GCI
CTRL	10-9C8-C1	SN	+++	−/+	−/+	−/+	−/+	−
		WM CTX		−	−	−	−/+	−
	14-9E7-A1	SN	+++	−/+	−/+	−/+	−/+	–
		WM CTX		−	−	−	−/+	−
	17-9D12-A1	SN	+++	−/+	−/+	−/+	−/+	−
		WM CTX		−	−	−	−/+	−
	18-3A5-H2	SN	+++	−/+	−/+	−/+	−/+	−
		WM CTX		−	−	−	−	−
	20-9E2-B8	SN	+++	−/+	−/+	−/+	−/+	–
		WM CTX		−	−	−	−	−
PD	10-9C8-C1	SN	+	+	+/++	+/++	+/++	−/+
		WM CTX		−/+	−/+	−/+	−/+	−/+
	14-9E7-A1	SN	+	+	+/++	++	++	−/+
		WM CTX		−/+	−/+	−/+	−/+	−/+
	17-9D12-A1	SN	+	−/+	+	+	+/++	−/+
		WM CTX		−/+	−/+	−/+	−/+	−/+
	18-3A5-H2	SN	+	−/+	+	−/+	+	−
		WM CTX		–	−/+	−	−/+	−
	20-9E2-B8	SN	+	+	+/++	+/++	+/++	−/+
		WM CTX		−/+	−/+	−/+	−/+	−
DLB	10-9C8-C1	SN	+	++/+++	++/+++	++/+++	+++	−/+
		WM CTX		−/+	+	−/+	+/++	−/+
	14-9E7-A1	SN	+	++/+++	++/+++	++/+++	+++	−/+
		WM CTX		−/+	−/+	−/+	++	−/+
	17-9D12-A1	SN	+	++	+/++	+/++	+/++	−/+
		WM CTX		−/+	−/+	−/+	+	−/+
	18-3A5-H2	SN	+	+/++	+/++	+	+/++	−/+
		WM CTX		−/+	−/+	−/+	−/+	−/+
	20-9E2-B8	SN	+	+++	++/+++	+++	+++	−/+
		WM CTX		−/+	−/+	+/++	++	−/+
MSA	10-9C8-C1	SN	+	+	−/+	+	+	++/+++
		WM CTX		−/+	−	−/+	+	++
	14-9E7-A1	SN	+	+	−/+	+	+/++	++/+++
		WM CTX		−/+	−/+	−/+	+	++
	17-9D12-A1	SN	+	−/+	−/+	−/+	+	++/+++
		WM CTX		−	−	−/+	+	+/++
	18-3A5-H2	SN	+	−/+	−/+	−/+	−/+	++
		WM CTX		−/+	−	−	−/+	+
	20-9E2-B8	SN	+	+	−/+	+/++	+	++/+++
		WM CTX		−/+	−/+	−/+	−/+	++

*CTRL* control, *DLB* Dementia with Lewy bodies, *PD* Parkinson’s Disease, *MSA* Multiple system atrophy, *SN* Substantia nigra, *WM CTX* white matter cortex.

In the PD patients, the highest quantity of pathological staining (i.e. LBs, LNs, neutropil “grains”, and extracellular inclusions) were found with mAbs 10-9C8-C1 (Table 5 and S3, and Fig. 7aI), 14-9E7-A1 (Table 5 and S4, and Fig. 7b), and 20-9E2-B8 (Table 5 and S7 and Fig. 7e). A lower number of pathological structures were found with mAb 17-9D12-A1 (Table 5 and S5, and Fig. 7c), while mAb 18-3A5-H2 (Table 5 and S6, and Fig. 7d) had very limited staining. No or little staining was found in the WM CTX of two of the PD patients, and only the patient with extensive pathology in the midbrain (PD191) also showed pathology in WM CTX (Tables S3-7 and Fig. 7, aIII-eIII). A similar pattern was observed in the DLB patients, however, while mAbs 18-3A5-H2 (Table 5 and S6 and Fig. 7dV, dVI) and 17-9D12-A1 (Table 5 and S5, and Fig. 7cV, cVI) showed similar ability to recognize pathology, the mAb 20-9E2-B8 found more LNs in the WM CTX compared to the others (Table 5 and S7, and Fig. 7eV-eVI), and some neuropil “grains” were also found in the WM CTX with mAbs 10-9C8-C1 (Table 5 and S3), 14-9E7-A1 (Table 5 and S4), and 20-9E2-B8 (Table 5 and S7). However, this group only includes two patients due to limited tissue availability. As expected, the MSA patients showed mainly GCIs, with the highest quantity found by mAbs 10-9C8-C1 (Table 5 and S3 and Fig. 7aVII, aVIII), 14-9E7-A1 (Table 5 and S4, and Fig. 7bVII, bVIII), and 20-9E2-B8 (Table 5 and S3 and Fig. 7eVII, eVIII), and to a lesser extent by mAbs 17-9D12-A1 (Table 5 and S3 and Fig. 7cVII, cVIII) and 18-3A5-H2 (Table 5 and S3 and Fig. 7dVII, dVIII). Here, as opposed to the other synucleinopathies, a high quantity of pathology, mainly GCIs, was also observed in the WM CTX (Table 5, Fig. 7aVII–eVII).

**Fig. 8:**
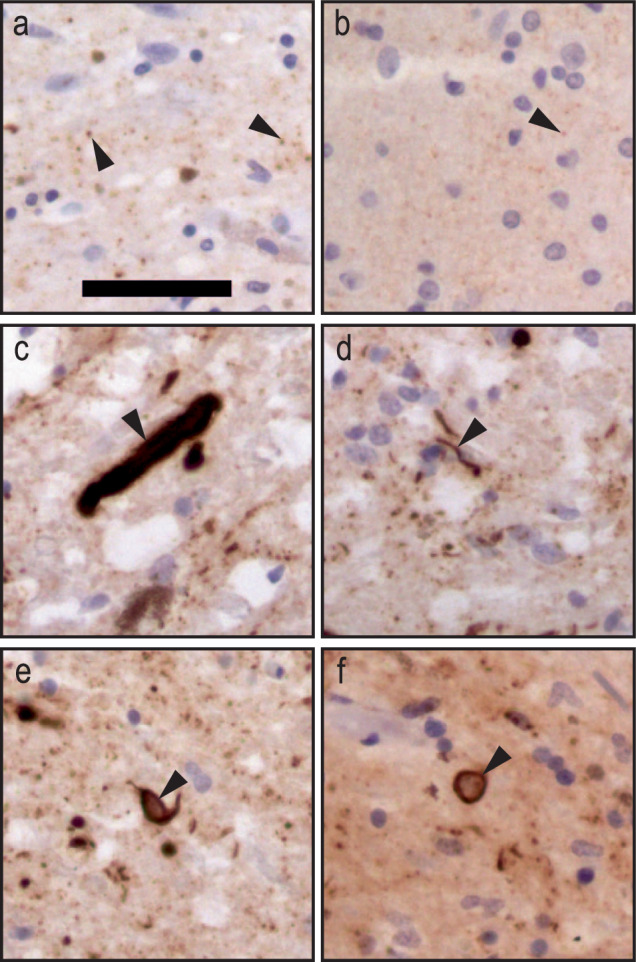
Morphologies observed in human post-mortem brain tissue but not quantified. **a**, **b** Small punctate staining, observed primarily with mAbs 10 9C8, 14-9E7-A1, and 17-9D12-A1. **c**, **d** Thick, coarse plump LN, and thin, thread-like LN, respectively. **e, f** Circular inclusions. Scalebar = 50 µm.

The mAbs 10 9C8, 14-9E7-A1, and 17-9D12-A1 showed small punctate staining (Figs. 7 and 8, arrowheads in a-b), which disappeared when omitting the primary Abs (Fig. S8a–h) and which seemed different to what was found with the commercial α-syn antibody (Fig. S8i). This was primarily observed in the SN and was present in all groups including the control brains. It is unclear whether this is monomeric human α-syn or background staining, hence this was not quantified. Two types of LN morphology were observed: thin thread-like, and thicker coarse-plump morphology (Fig. 8c, d). However, we did not distinguish between these two types of morphologies, which were both quantified as LNs. In the DLB and severe cases of PD, we observed a characteristic ring-shaped inclusion surrounding a nucleus (Fig. 8e-f). Similar morphology has been reported by others and described as a ring-shaped LB-like staining^64^, or as a coiled body-like oligodendroglia^65,66^. This could indicate that the mAbs may detect a pre-LB stage or another type of (early) pathology, consistent with their affinity for the αSO, but further investigations are needed to identify the identity of these structures. Ideally this should be combined with antibodies directed against chemical modifications such as N- and C-terminal truncations, phosphorylation and nitration which have been shown to be excellent tools to map out the distribution of various covalent forms of α-syn^54^.

## Discussion

We have generated >25 different mAbs raised against αSOs. Despite systematically testing mAbs from a range of different immunogens, we do not have unequivocal evidence that the mAbs are exclusively specific for αSOs (they all bind to fibrils in ELISA assays and recognize both monomers and fibrils in solution measurements). This is consistent with results from the commendably systematic work of Lashuel and coworkers^43^, where different antibodies are not confined to one species. However, both we and the Lashuel group see very different preferences for not just monomers, fibrils, and αSOs but also different types of αSOs (whether made alone or using chemicals such as HNE and dopamine). Furthermore, SPR experiments showed very high affinity (0.2-3 nM) of six different immobilized mAbs for αSOs and unconventional binding to monomers, highlighting (together with the inability to obtain robust data by biolayer interferometry) how sensitive these measurements are to the way in which αSOs and monomeric α-syn are presented to the antibodies. We believe that this combination of both general binding and a range of affinities reflects a large overlap of structural features between αSOs and fibrils, as well as a likely diversity of structures even within an apparently homogeneous αSO, given a spectrum of flexibilities in different regions of both αSOs and fibrils^19^. Such diversity is also seen even when the antigen is highly constrained from the beginning. This was seen in a study where mice were immunized with circularly constrained peptides designed to mimic a conformational state intermediate between monomeric and fibrillar α-syn: the resulting mAbs bound both PFFs and αSOs according to both SPR and ELISA^67^. Most of the mAbs recognize linear epitopes found in the α-syn sequence, suggesting they discriminate between monomers, oligomers, and fibrils based on avidity rather than absence of a particular epitope. Of these epitopes, most are found in the C-terminal region which is clearly most immunogenic (both due to its high exposure and high concentration of charged residues), in accord with previous studies^48,68^. However, 6-7 mAbs recognize α-syn truncated at residue 100; for those we could analyze by peptide array studies, binding sites were found localized in the NAC region (cf. Figure 3d). Furthermore, at least one mAb (19-2C3) showed high affinity for the αSO by FIDA (4.2 nM) but weak binding to the monomeric sequence displayed on peptide arrays, indicating recognition of a structural epitope only found in aggregated α-syn. Interestingly, this mAb consistently showed good staining for pS129 in tissue analysis, marking it out as a particularly promising mAb for diagnostic purposes. Two other mAbs (18-9E10B1 and 19-1D2) showed robust binding to αSO but no specific binding to the peptide array, suggesting potential for aggregate specificity.

Structural diversity is also likely to be seen in vivo where there are even greater options for variation due to covalent alterations such as truncations and post-translational modifications^54^. In general, a better understanding of synucleinopathies requires deployment of a diverse set of antibodies that have been characterized thoroughly by complementary techniques, providing as detailed a view of disease pathology as possible. Both for therapy and diagnostics, it can be convenient to have mAbs that show broad specificity for a range of α-syn aggregates, provided they do not show background binding to monomeric α-syn. Specific affinity could also be boosted further for those amongst our mAbs that (in contrast to the commercial antibody MJF14) show several orders of magnitude stronger binding to αSOs than monomeric α-syn. These mAbs could be starting material for antibody engineering in scFv-libraries where Complementary Determining Regions from the best mAbs are grafted into a scaffold and used in e.g. a yeast display system under continuous evolution to improve binding to a given target such as the αSO, identifying the best hits through Fluorescence-Assisted Cell Sorting (FACS)^69^. Such antibodies might be used to identify particular sites of aggregation within the brain when combined with e.g. PET ligands, as already demonstrated for Aβ aggregates^70^, as well as enhancing signals to improve the sensitivity of detection of oligomers in cerebrospinal fluid using oligomer-binding peptides such as PSMα3^40^. In addition, they could aid the detection of pathological α-syn in peripheral biosamples and tissues, such as skin^71^, olfactory mucose^72^, and saliva^73^ alone or in combination with seeding techniques. For therapeutic purposes, they could help target clearance through the Fcγ receptor^74^, which is expressed in microglia and other myeloid cells, such as monocytes/macrophages. Indeed, passive immunization approaches have been shown to be neuroprotective in animal PD models, and several anti-α-syn antibodies are being currently tested in the clinic^2,75^. We also speculate that our antibodies might help to measure longitudinal changes on the level of oligomeric forms in patients, which could potentially assist to evaluate disease progression or efficacy of α-syn targeting therapies. The field currently lacks longitudinal analysis of oligomeric α-syn in patients, and our antibodies are good candidates to be used for those.

Our histological analysis confirmed the ability of the mAbs to recognize pathological α-syn in brain tissue. Moreover, their signal did not completely overlap with commercial antibodies, but could recognize unique structures not seen by the MJF14 nor the pSer129 commercial antibodies, as shown by the low coincided index we obtained. It is reassuring to note that all five mAbs selected for immunohistochemical analysis on human patient material were able to find α-syn-related pathologies like LBs and LNs in PD and DLB and GCIs in MSA patients, although to different extents, with mAbs 10 9C8-C1, 14-9E7-A1, and 20-9E2-B8 showing the highest number of pathological structures detected, and of these, 20-9E2-B8 showed the lowest background binding. These three antibodies could recognize more α-syn in axonal swellings in the α-syn-rAAV PD model that was not recognized by the Ser129 antibody, which highlights their ability to detect pathological α-syn. Interestingly, of these three mAbs, 10 9C8-C1 and 14-9E7-A1 also recognized some punctate staining in the high α-syn expressing area of the rat hippocampus that was not eliminated when preincubated with monomeric (human) α-syn. Moreover, 10 9C8-C1 and 14-9E7-A1 also showed small punctate staining in the human SN, also an area with high α-syn expression, which was not seen with the commercial α-syn antibody. It remains to be determined whether the punctate structures stained by these two mAbs relates to some form of αSO naturally occurring in areas with high α-syn expression (i.e. hippocampus in rat SN and in humans). Collectively, our histological data suggest that these three mAbs are useful for pathological evaluation of *postmortem* brain human tissue. The ability of these mAbs to reveal α-syn pathology in other tissues, such as skin or gut, or to detect αSOs in biofluids, needs to be evaluated. If confirmed, they could be used for diagnostic purposes. In addition, their putative therapeutic potential will require future studies in appropriate cell lines and animal models.

It is difficult to make a straightforward correlation between the biophysical properties of the mAbs developed in this study and their immunohistochemical properties. It should be noted that the histological examinations were all done in PFA or formalin-fixed tissue, which might modify the epitope of otherwise biologically relevant forms of α-syn such as αSOs and fibrils. If the α-syn pathological deposits detected in tissue are mainly fibrillar, then the best indicator for strong immunohistochemical binding could reasonably be expected to be in vitro-measured fibril affinity (assuming the in vitro fibrils correspond to the in vivo deposits). If in contrast αSOs are found to a larger extent in the deposits, then αSO affinity would be a good indicator (again assuming the in vitro αSO corresponds to its in vivo counterpart, which remains unproven). However, the five mAbs selected for in-depth immunohistochemical analysis (based on their ability to complement MJF14 and pSer129) were not significantly different from the remaining 11 mAbs in terms of ability to bind to the monomer in ELISA competition assays, blocking of calcein release or effects on ThT-mediated α-syn fibrillation. The 5 selected mAbs do however consistently show a high affinity for αSOs, particularly the “top three” (mAbs 10 9C8, 14-9E7-A1, and 20-9E2-B8, which detected the highest number of pathological structures); 20-9E2 gave rise to a peculiar behavior in FIDA which prevented proper determination of *K*_D_^αSO^, but this could simply reflect a strong tendency to agglutinate with the αSO in solution. It is also worth noting that this latter mAb also showed the lowest immunohistochemical background signal, consistent with very weak binding to α-syn monomers (*K*_D_^monomer^ is 3.8 µM, the highest of the 16 mAbs selected). All of these considerations emphasize that there is no simple relationship between antibodies’ biophysical and immunohistochemical properties. While this is perhaps disappointing from a narrow perspective, it is conversely encouraging that a large collection of mAbs shown in vitro to be able to recognize different aggregated α-syn species also show immunohistochemical usefulness.

Access to mAbs recognizing different α-syn pathological forms holds significant potential in expanding our understanding of affected brain regions in pathological conditions. This has implications for neuropathological assessment and diagnostic confirmation. α-syn aggregation is recognized as the pathological hallmark for synucleinopathies, however, αSOs may be better indicators of early-stage pathology, and GCIs and LBs of late-stage pathology^76,77^. This distinction is important for the development of α-syn positron emission tomography tracers to monitor disease progression, particularly in its initial phases^78,79^. Additionally, efforts to create assays for detecting αSO pathogenic protein forms in the cerebrospinal fluid are underway^39,80^, making access to mAbs recognizing α-syn toxic αSOs highly valuable. In these samples, the α-syn aggregates are not fixated and will present unmodified epitopes to the mAbs, equivalent to the in-solution encounters we probe by FIDA. Knowledge about the relative affinities of our mAbs for different aggregate states in solution as provided by FIDA studies is valuable input for optimization of such assays.

## Methods

### Materials

Unless otherwise stated, all chemicals were of the highest grade possible and obtained from Sigma-Aldrich (St. Louis, MO).

### Recombinant expression and purification of α-syn

Human α-syn was expressed recombinantly in *Escherichia coli* and purified using a combination of osmotic shock, acid precipitation, ion exchange and dialysis into milliQ water. Our work builds on our previous protocols^21^ but is here described in more detail in an updated version. Two LB-agar plates with premade LB-medium were prepared per 1 L auto-induction medium. *E. coli* BL21/DE3 transformed with the α-syn expression vector pET11d was plated out on the agar plates and incubated overnight at 37 °C. The autoinduction medium^81^ was prepared with 1 M MgSO_4_ ∙ 7H_2_O (Fisher Scientific, >99%), 20x NPS solution (per liter: 66 g ammonium sulfate (≥99%),136 g anhydrous potassium dihydrogen phosphate (Fisher Scientific, ≥99%) and 142 g sodium phosphate dibasic dihydrate (≥99,5%)), and 50×5052 solution (per liter 250 g glycerol (Sigma-Aldrich, ≥99%), 25 g glucose (Fisher Scientific, ≥99,8%), and 100 g lactose (≥99%)). Ampicillin (100 μg/mL, ≥98%) was added to the media right before usage. The colonies were transferred from the plates to the medium by scraping them off. The bacteria were incubated for approximately 6 h until they had reached an OD_600_ of 1.8–2.0 (in practice it takes ca. 2 h to reach an OD_600_ of 0.6–0.8 and an additional 4 h to reach OD 1.8) at 37 °C at 120 rpm shaking. No induction was required due to autoinduction. The cells were then harvested by centrifugation for 20 min at 4000 rpm at 4 °C in a Sorvall Lynx 6000 centrifuge (Thermo Scientific, Waltham, MA). The supernatant was discarded, and the pellets were collected in falcon tubes with 3 mL of 1x phosphate buffered saline (PBS). For cell lysis, pellets were resuspended in osmotic shock buffer (30 mM Tris-HCl, 40 g/L sucrose (Fisher Scientific) and 2 mM EDTA, final pH 7.4) and centrifuged for 30 min at 7000 *g* at 4 °C. The supernatant was then carefully discarded (the cell pellet is not very dense), and the pellet was dissolved in ice-cold water (100 mL per 1 L medium). When dissolved, a solution of saturated MgCl_2_ (Alfa Aesar, 99.99%) was added to stabilize the protein (40 µL per 100 mL suspension). The protein was centrifuged again for 30 min at 9000 *g* at 4 °C, and the pellet was discarded. The supernatant was then acidified to pH of 3.5 with HCl (using a 6 M HCl solution for initial adjustment followed by 1 M for final adjustment) and immediately centrifuged for 20 min at 9000 *g* at 4 °C. The pellet was again discarded, and the pH of the supernatant was adjusted to 7.5 with NaOH (6 M for initial adjustment and 1 M for final adjustment). The protein was filtered through a 0.22 µm filter and purified on the ÄKTA pure system with a Hitrap DEAE FF Q HP Q-sepharose column (GE Healthcare) pre-equilibrated with buffer A (20 mM Tris-HCl pH 7.5). Sample was loaded and then washed with 10% buffer B (buffer A + 1 M NaCl) and eluted with a gradient of 10-50% buffer A. α-syn elutes at around 25–30% buffer B. The protein fractions were analyzed on an SDS-PAGE gel and pure fractions were pooled and dialyzed overnight against milliQ water with 3 exchanges. Subsequently the protein was lyophilized (Labogene Scanvac Coolsafe) for two days and stored at –80 °C.

### Preparation of αSOs and α-syn fibrils

To make unmodified αSOs, we used an approach optimized from a previous protocol^21^. Lyophilized monomeric α-syn was dissolved to a final concentration of 9–10 mg/mL in degassed 1x PBS (per liter: 8 g NaCl, 0.2 g KCl, 1.44 g anhydrous Na_2_HPO_4_ and 0.24 g anhydrous KH_2_PO_4_, all adjusted to pH 7.4 with NaOH), passed through a 0.22 μm filter and aliquoted as 1 mL samples in Eppendorf tubes with 1 mL in each tube. The samples were incubated for 3–5 h in a shaking incubator at 37 °C with 900 rpm agitation in a dry bath shaker (Thermo Scientific, Waltham, MA), centrifuged at 12,000 *g* for 5 min to remove insoluble protein aggregates and the supernatant transferred to a Superose 6 prep grade XK 26/100 gel filtration column (GE Healthcare, Chicago, IL) pre-equilibrated with 1x PBS on an ÄKTA pure system. The αSO fraction eluting as a single peak around 13-16 mL was concentrated using a 100 kDa cut-off Amicon Ultra-0.5 conical ultrafiltration unit (Merck, Darmstadt, Germany) at 4 °C and stored at −20 °C until needed. αSOs with (-)-epigallocatechin-3-gallate (EGCG) were prepared using 560 µM α-syn and 6.5 mM EGCG using the same steps as for unmodified α-syn. HNE-αSOs and ONE-αSOs were prepared as described^31,82^. Briefly, 200 µM α-syn was mixed with either 0.8 mM ONE or 3.2 mM HNE, incubated at 37 ^o^C while shaking at 550 rpm for 24 h, centrifuged to remove insoluble material and purified by gel filtration as above. HNE-αSOs eluted slightly earlier than the other αSOs due to their larger size.

### Preparation of α-syn-linked cVLPs

The major coat protein of the *Achinetobacter phage*, AP205 was genetically fused at the N-terminus to the split-protein SpyCatcher (Genbank Nucleotide entry OK422508.1). The fusion protein was subsequently expressed in *E. coli*, as previously described^50^. Self-assembled capsid virus-like particles (cVLPs, containing 180 coat proteins per cVLP and thus 180 binding sites for α-syn) were purified by ultracentrifugation on an Optiprep density gradient and dialyzed into PBS pH 7.4 using a 1000 kDa cutoff dialysis tube (SpectrumLabs). Total protein concentration was determined using the bicinchoninic acid (BCA) assay (Thermo Scientific) according to the manufacturer’s protocol. To formulate cVLP α-syn vaccines, SpyCatcher-displaying cVLPs were mixed with sufficient molar excess (at least 1:2) of α-syn antigens (SpyTag-α-syn(1-100) and SpyTag-α-syn(1-120) where α-syn is genetically fused to the C-terminus of the SpyTag peptide) to ensure maximum coupling efficiency (unreacted antigen was monitored on SDS-PAGE). Antigens containing full-length α-syn (1-140) did not lead to successful coupling to cVLP. The vaccine formulations were then incubated overnight at 4 °C, whereafter contaminant LPS was removed by a phase extraction method using Triton-X114, as described^83^. Excess antigen and residual Triton-X114 were removed by dialysis into PBS, pH 7.4 using a 1000 kDa cutoff (Spectrum Laboratories, Rancho Dominguez, CA). Purified vaccines were quality checked by a spin stability test (2 min at 16000 g), comparing an equal volume of pre- and post-spin samples on SDS-PAGE to assess potential precipitation, indicating vaccine aggregation.

### Immunization of mice and expression

All animal experiments were carried out by qualified staff according to the ethical rules of the animal committee at the Department of Biomedicine, Aarhus University (license 2017-15-0201-01319 from the Danish Supervisory Authority on Animal Testing). Balb/c female mice were immunized in groups of 7–8 once per week for 3 weeks with 25 µg α-syn antigens in 10 µL PBS pH 7.4 (Table 1). Immediately prior to immunization, Incomplete Freund’s Adjuvant was added 1:1 (v/v) and vortexed thoroughly. Mice were anaesthetized with isoflurane (5% for induction, 2–3% for maintenance), 1.2 L/min of O_2_ and 0.6 L/min of atmospheric air in chamber and injected in the thigh muscle. 1 week after final immunization, blood was drawn from the mouth region, and serum was prepared and tested for antibodies against α-syn monomer, αSO and fibrils using indirect ELISA (details below). Mice with a strong response (OD_450_ > 1.5 at 450 nm) were boosted 1 week later to exploit the secondary immune response as follows: 50 µg antigen (cVLPs or αSOs) were injected intraperitoneally 3 and 2 days before the fusion. The mice were then sacrificed, and the spleen was removed and homogenized. Cells were washed in RPMI (Lonza, Basel, Switzerland) medium and mixed 2:1 (cell:cell) with NS-1 cells which were prepared 1 week earlier. 3 ml 50% PEG6000 was carefully added to each spleen preparation, after which the cells were washed and distributed into ten 96-well plates in suitable dilution series. The cells were grown in HAT (hypoxantine, aminopterin and thymidine) medium to select for fused cells. 10–14 days after the fusion, the hybridoma cells were screened by indirect ELISA, using 100 µl of medium from each well mixed with 100 µl 1x PBS. Wells with strong response (OD_450_ > 1.5) were selected for sub-cloning. Cell count was estimated in suspended cell solutions using a counting chamber. Cells were diluted to 50, 5 and 0.5 cells/100 µl in HAT medium and 100 µl of each solution was transferred to 96-well plates containing macrophages harvested from balb/c mouse (one mouse per plate). The cells were screened after 10–12 days using indirect ELISA. Positive clones were removed to a 24-well plate and tested with competition ELISA, a modification of the indirect ELISA assay in which the plate was coated with 2 µg/mL αSO, and the supernatant was preincubated with different concentrations of α-syn monomer (2 mg/ml down to 0.002 mg/ml in 2-fold dilution steps) for 30-60 min at room temperature before transfer to the plate.

### Purification of monoclonal antibodies (mAbs)

Clones with strong response to the αSO in competition ELISA (i.e. binding to the αSO despite the presence of monomeric α-syn) were grown up in 150 mL-flasks (Sarstedt) in 100 mL medium. Culture medium was harvested every 2–3 days and combined to a total of ca. 1 L (over a period of 3–4 weeks). The solution was mixed 1:1 with binding buffer (1 M Gly pH 8.5 and 150 mM NaCl). pH was checked to be 8.5 using litmus paper and filtered using 0.45 µm filter. The solution was injected onto a 5 mL Gamma Bind Plus column (Cytiva Europe, Brønshøj, Denmark) at 3 mL/min and washed with binding buffer until baseline at 280 nm is reestablished. Monoclonal antibody (mAb) was eluted using elution buffer (0.1 M Gly adjusted to pH 2.7 with HCl) in fractions of 3 mL which upon elution were immediately neutralized with 300 µL 1 M Tris-Cl pH 8.0 (already present in the fraction collection tubes). All peaks were analyzed by SDS-PAGE. The mAb fractions were pooled and dialyzed against 1xPBS and stored at –80 ^o^C. mAbs were named according to the mouse from which they were obtained (#1–20, cf. Table 1) followed by the plate number and the well(s) from which they were grown and cloned to purity. For example, mAb 14-2B8-G1 is produced in mouse 14 (immunized with unmodified αSO) from a clone which was first obtained from plate 2’s well B8 in the first plating and from well G1 in a subsequent sub-plating from this well. A few mAbs did not need subplating and therefore only have one extension from the mouse number.

### Indirect ELISA assays

A 96-well ELISA plate (High Bind; F, Sarstedt, Nümbrecht, Germany) was coated with 2-fold dilution series of *α*-syn monomer or αSO (added as 60 µL with a starting concentration of 2.0 µg/ml and 7 additional dilution steps down to 0.0156 µg/ml) and incubated overnight at 4 °C. The plate was then emptied by inversion and gentle tapping on a table, after which it was blocked with 75 μL 15% heat-inactivated qualified FBS (Gibco) in PBS (Lonza, BioWhittaker) for 30 min at 37 °C in a VWR INCU-Line incubator and then washed in PBS with 0.05% Tween20 three times on a Intelispeed Washer IW-8 (BioSan, Riga, Latvia). The plate was incubated with 50 μL of a 2-fold dilution series (2.0-0.002 μg/mL) mAb for 1 h at 37 °C and washed three times with PBS. 50 μL secondary Jackson GAM-HRP (Horse Radish Peroxidase) antibody (Trichem, 115-035-146) (1:20000) was added for 1 h at 37 °C. The plate was again washed, and 50 μL TMB ONE (Kementec, Taastrup, Denmark) was added for 30 min at 37 °C with minimum light exposure. The reaction was stopped by adding 100 μL 0.5 M sulfuric acid. Absorption was finally measured at 450 and 620 nm on a HiPo Microplate Photometer MPP-96 (BioSan, Riga, Latvia). To test for binding to Aβ40 and tau, we used 2 µg/mL Aβ40 (Chinese Peptide, 95% purity) and 2 µg/ml tau produced recombinantly as described^84^ and incubated with a dilution series of 5 different mAbs (10-9C8-C1, 14-9E7-A1, 20-9E2-B8, 17 9D12 A1 and 18 3A5H2) from 10 µg/ml and down in 10 steps of 2-fold dilution, followed by the steps described above. As positive controls we used anti-tau antibodies 46 32274, 5 58860 and 13 21796 (Santa Cruz, CA).

For competition assays, the ELISA plate wells were all coated with 2.0 µg/ml αSO and blocked as described above. 2.0 µg/ml mAb was pre-incubated with 0.004-2 mg/ml *α*-syn monomer for 1 h at room temperature before adding this solution to the plate wells. Bound mAb was detected as described above.

### FIDA of mAb affinities for αSOs and α-syn

α-syn monomer and αSOs were labelled with amine-reactive Alexa 488 succinimidyl ester (ThermoFisher, Waltham, MA) at a molar protein:Alexa488 ratio of 1:4 in 0.1 M sodium bicarbonate. After incubation for 2 h at room temperature in the dark, the sample was desalted into PBS on a PD-10 desalting column (Cytiva, Brønshøj, Denmark). All FIDA experiments were carried out on a Fida1 apparatus (Fida Biosystems, Søborg, Denmark) using either 50 nM αSOs or 100 nM α-syn monomers together with a 2-fold dilution series of mAbs from 2 µM down to 0.01 nM in a series of in-capillary mixing experiments utilizing a standard five-step method^85^ in triplicates at 25 °C, and using MilliQ water to clean and 0.05% pluronic acid in PBS to equilibrate the standard 75 µm capillaries. The resulting Taylorgrams were analyzed using the FIDA bio software, where the monomer datasets were analyzed using single-species fitting, whereas αSO datasets were fitted with two-species fits with the fraction setting set to 75% for all data points. All resulting isotherms were fitted with a 1:1 binding model^86^:

1 $${R}_{{app}}=\frac{1+\frac{1}{{K}_{D}}\left[A\right]}{\left({\left({R}_{I}\right)}^{-1}-{\left({R}_{{IA}}\right)}^{-1}\right)+\left(1+\frac{1}{{K}_{D}}\left[A\right]\right){\left({R}_{{IA}}\right)}^{-1}}$$

Here *K*_D_ is the dissociation constant, [A] is the analyte concentration, and *R*_I_ and *R*_IA_ are the hydrodynamic radii (*R*_h_) of the indicator (monomeric α-syn or αSO) alone and in complex with the mAb, respectively.

### Integrity of αSOs in the presence of mAbs

The integrity of the αSOs in presence of mAbs 16-9E5, 20-5H12-C10, and 20-9E2-B8 was tested by incubation of individual mAbs at 5 µM with 0.5 µM of αSO for 20 min at RT. Presence of αSOs and potentially monomeric α-syn was then investigated by SDS-PAGE using a 12.5% acrylamide gel cast in-house, run at 150 V for 75 mins at RT, and stained with Coomassie Brilliant Blue dye. Pure mAbs and monomeric α-syn at known concentrations were run as controls. The gel was scanned using GelDoc Go System and analysed with Image Lab (both Biorad, Hercules, CA).

### Biolayer interferometry assay

BLI binding experiments were performed on an Octet RED96 (ForteBio, Menlo Park, CA, USA) and analyzed using either the instrument’s software or Prism (GraphPad Prism 5.0) software. 96-well plates (black, flat bottom, Greiner) were used. In general, binding analysis was performed at 25 °C and an orbital shake speed of 1000 rpm was used. Screened mAbs (2.5 μg/mL) in buffer (PBS pH 7.4 supplemented with 0.1 mg/mL BSA and 0.02% Tween-20) were captured onto Protein G sensors (Octet Protein G (ProG) Biosensors, Sartorius, Goettingen Germany). Baseline was recorded to establish initial BLI signals prior to each binding event (including association, dissociation, and regeneration steps). First, the antibody-coated sensor was dipped into the αSO solution at 600 nM for 200 s (association step) and then into buffer for 100 s (dissociation step). Finally, three cycles of regeneration were per-formed, consisting of first dipping the sensor into a glycine solution (10 mM at pH 1.4) and then into buffer for 5 s each. The process was repeated for each mAb tested. Data shown (Fig. S4) corresponds to the BLI signal at the end of the association phase (*t* = 195 s). For binding kinetic assays, selected mAbs at a concentration of 2.5 μg/mL in buffer were loaded on Protein G sensors until 0.8 nm response was reached. Baseline was recorded prior to binding measurements. The binding kinetics were recorded as follows: eight mAb-loaded sensors were dipped in 1/2 serial dilutions of αSO for 400 s during the association step. Then, the sensors were moved to only buffer for 1200 s (dissociation step). Regeneration was achieved by doing three cycles of consecutive steps, as stated above. Binding sensorgrams were aligned to dissociation, following subtraction of the reference well/sample and globally fit to a 1:1 binding model.

### Surface Plasmon Resonance (SPR) experiments

SPR experiments were carried out on a Biacore 3000 instrument (Cytiva). For oriented capture of monoclonal antibodies, two adjacent flow cells of an HC200M chip (XanTec bioanalytics) were coated with protein G (08062, Merck) using standard amine chemistry (EDC/NHS activation). Residual reactive groups were blocked by a 7 min injection of 1 M ethanolamine pH 8.5. Antibodies were captured in flow cell 2 only (the active flow cell), leaving flow cell 1 as an in-line reference. The antibody capture levels were approximately 250-300 RU and 3500-4000 RU for analysis of binding to α-syn oligomer and monomer, respectively. Next, a two-fold titration series of α-syn (typically 2 µM down to 31.5 nM, monomeric concentration units), was injected over the surfaces for 180 s followed by a 600 s dissociation phase (120 s association and 300 s dissociation in the case of the monomer). The running buffer was 10 mm NaH_2_PO_4_, pH 7.5, 150 mM NaCl and 0.05% Tween 20 and the flow rate was 30 µl/min. Binding analysis was performed at 25 °C, and data were collected at a rate of 1 Hz. Using the BIAevaluation 4.1.1 software (Cytiva), recorded signals from the active flow cell were double referenced, i.e. the signal from the in-line reference flow cell was subtracted as was the signal from a blank run (0 nM analyte). Affinities were estimated using global fitting in the built-in “association then dissociation” model of GraphPad Prism 10.1.0.

### Preparation of peptide arrays

μSPOT peptide arrays^87^ were synthesized using a MultiPep 2 parallel peptide synthesizer (CEM) on acid labile, amino functionalized, cellulose membrane discs (CEM) containing 9-fluorenylmethy- loxycarbonyl-β-alanine (Fmoc-β-Ala) linkers (minimum loading 1.0 μmol/cm). Synthesis was initiated by Fmoc deprotection using 20% piperidine in *N*-methylpyrrolidone (NMP) (1 × 2 and 1 × 4 μL, 2 and 5 min, respectively) followed by washing with dimethylformamide (DMF, 7 × 25 μL per disc) and ethanol (EtOH, 6 × 25 μL per disc). All couplings were achieved using 1.2 μL of coupling solution consisting of preactivated amino acids (AAs, 0.5 M) with ethyl 2-cyano-2-(hydroxyimino)acetate oxyma (1.5 M) and N,N′-diisopropylcarbodiimide (DIC, 1.1 M) in NMP (2:1:1, AA:oxyma:DIC). The couplings were carried out 5 times (5, 15, 15, 30, and 30 min, respectively), and subsequently, the membrane was capped twice with capping mixture (5% acidic anhydride in NMP), followed by washes with DMF (7 × 25 μL per disc). After chain elongation, final Fmoc deprotection was performed with 20% piperidine in NMP (2 × 4 μL, 5 min each), followed by 7× washes with DMF, subsequent N-terminal acetylation with capping mixture (2 × 4 μL, 5 min each) and final washes with DMF (7 × 25 μL per disc) and EtOH (7 × 25 μL per disc). Dried cellulose membrane discs were transferred to 96 deep-well plates (max. volume 1 mL per well) and were treated with the side-chain deprotection solution consisting of 80% TFA, 12% DCM, 5% H_2_O, and 3% TIPS (150 μL per well) for 1.5 h at room temperature (rt). Afterward, the deprotection solution was removed, and the discs were solubilized overnight at rt using a solvation mixture containing 88.5% TFA, 4% trifluoromethanesulfonic acid (TFMSA), 5% H_2_O, and 2.5% TIPS (250 μL per well). The resulting peptide-cellulose conjugates were precipitated with ice-cold ether (700 μL per well) and spun down at 1000 rpm for 90 min, followed by an additional wash of the formed pellet with ice-cold ether. The resulting pellets were redissolved in DMSO (250 μL per well) to give final stocks, which were transferred to a 384-well plate and printed (in duplicates) on white coated CelluSpots blank slides (76 × 26 mm, Intavis AG) using a SlideSpotter robot (Intavis AG).

#### Binding of monoclonal antibodies to peptide arrays

To analyze the binding of mAbs to peptide arrays presenting 14-mer peptides sequentially covering the sequence of α-syn, we proceeded as follows. Arrays were blocked overnight in 1x PBS with 1% BSA and 0.05% Tween 20 at 4 °C, washed three times in PBS with 0.05% Tween 20, incubated for 1 h at 50 rpm on a rolling table (Thermo Scientific) at room temperature with 3 ml of 1 µg/ml mAb in 1x PBS, 0.05% Tween 20 and washed three times in 1x PBS with 0.05% Tween 20. The arrays were then incubated for 1 h at 50 rpm on a rolling board at room temperature with 3 ml of a solution of secondary antibody (Jackson GAM-HRP, diluted 1:20000 in PBS). After washing the arrays three times in PBS with 0.05% Tween 20, they were stained with 2–3 ml of TMB-D blotting (Kementec) at room temperature. After drying, the arrays were then scanned in the Bio-rad GelDoc Go Imaging System. The scanned images were analyzed in the MODified™ Histone Peptide Array program to obtain intensity values for individual spots. Duplicates for each array were averaged and data were normalized to the intensity of the highest measured intensity (mAb 3-8C4). Finally, averaged background values (typically relative units of 0.1) were subtracted.

### Differential scanning fluorimetry

Approximately 10 µl of 0.2 mg/mL of mAbs in PBS were transferred to NanoTemper TY-C001 capillaries and analyzed on a Tycho NT.6 (NanoTemper, Watertown, MA) at a scan rate of 30 ^o^C/hr. Data are provided as the ratio between emission at 330 and 350 nm after excitation at 280 nm. Inflection points were determined as the peak position in first-derivative plots of the ratio 330/350 versus temperature, according to the manufacturer’s instructions.

### Affinity of mAbs for fibrils

Fibrils were prepared by shaking ~2 mg/mL α-syn at 37 °C and 200 rpm in an Innova 4400 Incubator Shaker (New Brunswick Scientific Inc., Edison, NJ) for 72 h. The fibrils were centrifuged at 21,000 *g* (Centrifuge 5425 R, Eppendorf, DE). The supernatant was removed, and the fibrils were washed with PBS for 3 times. The fibrils were sonicated at 10% power, 30% cycle time for 1 min with a Sonoplus Bandoline sonicator. Fibril concentration was measured as monomer equivalents after dissolution in 4 M GdmCl in a Corning 3679 96-well plate (Corning Inc., Corning, NY) at 275 nm on a FLUOstar OPTIMA plate reader (BMG Labtech, Ortenberg, Germany) using an extinction coefficient of 5600 M^−1^ s^−1^ (path length 0.63 cm). Fresh monomer was then incubated with 10% (w/w) seeds under the same conditions. Fibrils were harvested according to the protocol described above,sonicated for 1 min, and their concentration was determined as described above. Affinity was measured using microfluidic diffusional sizing (MDS) in microfluidic devices as described^52,59^. 2 nM to 20 µM monomer equivalents of fibrils were mixed with 100, 200, or 500 nM Alexa 647-labelled mAb and subsequently incubated for 15 min. The sample was loaded onto the microfluidic chip along with PBS as co-flow buffer, and diffusion into the surrounding buffer medium was monitored at four positions along the diffusion channel. For imaging, a custom-built inverted epifluorescence microscope equipped with a Prime 95B CCD camera (Photometrics, Tuscon, AZ) brightfield LED light sources (Thorlabs, Newton, NJ) and a Cy5-4040C-000 Semrock Filter set (Laser 2000, Huntingdon, UK) was used. The apparent *R*_h_ of the mAbs and fibril bound fractions were determined by image analysis in a two-species model according to the diffusion-advection equations for transport under flow^88^. The fractions were fitted to a quadratic equation:

2 $${F}_{b}=\left(\frac{{{mAb}}_{0}+\frac{{\alpha -{syn}}_{0}}{n}+{K}_{d}}{2}-\sqrt{{\left(\frac{{mA}{b}_{0}+\frac{{\alpha -{syn}}_{0}}{n}+{K}_{d}}{2}\right)}^{2}-{{mAb}}_{0}* \frac{{\alpha -{syn}}_{0}}{n}}\right)* \frac{{plateau}}{{{mAb}}_{0}}$$

where *F*_*b*_ is the fraction bound, *mAb*_*0*_ is the initial mAb concentration, α-syn_0_ is the fibril concentration in monomer equivalents, *n* is the number of mAbs bound per α-syn monomer and *K*_d_ is the dissociation constant. Plateau, *K*_d_, and *n* were assumed to be the same across the different mAb concentrations, and *n* constrained to be > 1.

### Thioflavin T fibrillation assays

Fibrillation of α-syn was carried out as described previously^89^. 0.25 mg/mL SEC-purified α-syn was incubated with 0.5 mg/mL mAb under constant shaking and using two beads, with a final volume of 150 μl at 37 °C in a 96-well Nunc plate (Thermo Fisher Scientific, Roskilde, Denmark) sealed with clear tape (Hampton Research, Aliso Viejo, CA). 40 µM Thioflavin T (ThT) was used to follow fibrillation with excitation and emission wavelengths of 448 and 485 nm, respectively. For seeding experiments, 0.5 mg/ml monomeric α-syn was incubated with 0.1 mg/ml mAb and 1% (50 µg/ml) sonicated fibrils (but no beads) while shaking.

### Calcein release studies

Calcein release was done essentially as described^90^. Calcein-filled vesicles were prepared by adding 1,2-dioleoyl-sn-glycero-3-phospho-(1’-rac-glycerol) (DOPG) powder to a solution of 46 mM calcein (salt form; adjusted to neutral pH using NaOH) in PBS to a final lipid concentration of 10 mg/mL. The suspension was subjected to 10 freeze-thaw cycles (liquid nitrogen and 45 ^o^C water bath), followed by 21 rounds of extrusion through a 100 nm filter (Avanti Polar Lipids, Alabaster, AL). Vesicles were separated from free calcein with a PD-10 column and fractions selected by measuring fluorescence (excitation at 485 nm and emission at 520 nm) before and after adding Triton X-100 to 0.16% or ca. 11 x its critical micelle concentration (2.5 µL of a 10% Triton X-100 solution was diluted into 150 µl, leading to 100% release of trapped calcein). Nano-tracking analysis showed the vesicles to be highly uniform with a size of 121.1 ± 0.5 nm. The selected vesicle solutions were used to calibrate αSO-driven calcein release using a 2-fold dilution series spanning from 100 µg/ml down to 0.05 µg/ml αSO. Typically, 3-6 µg/ml αSO led to 50% release of calcein. For inhibition studies, 6 µg/ml αSO was mixed with 100 µg/ml mAb and incubated for 10 min before adding 2.5 µl vesicle solution (ca. 5 mg/ml, i.e. final vesicle concentration ~0.08 mg/ml) and measuring fluorescence *F* after 30 min incubation. The percentage inhibition was calculated as follows (where subscripts refer to the material added to the vesicles):

3 $$\% {\mathrm{inhibition}}=100* \left(1-\frac{{F}_{{\rm{mAb}}+{\rm{\alpha }}{\rm{SO}}}-{F}_{{\rm{mAb}}}}{{F}_{{\rm{\alpha }}{\rm{SO}}}-{F}_{{\rm{buffer}}}}\right)$$

Effectively the fraction in Eq. 1 calculates how much of the release caused by the co-incubated αSO and mAb is caused by αSO alone (the numerator), normalized to the total amount of calcein released by free αSO (the denominator). All experiments were done in both technical and biological duplicates. We excluded mAbs for which addition of mAb alone to the vesicles led to a calcein release > ~ 40% of the release caused by free αSO.

### ROS toxicity cellular assays

The cellular toxicity assay measured reactive oxygen species using the cell-permeable reagent 2’, 7’-dichlorofluorescein diacetate (DCFDA) (Abcam, Cambridge, UK) which is converted to the highly fluorescent dichlorofluorescein upon oxidation. 20 mM DCFDA in DMSO was diluted to 20 µM in complete media with 10% FBS (Fetal Bovine Serum) without phenol red: to make a 20 μM final concentration, add 10 μL of 20 mM DCFDA solution to10 mL media and used straight away.

The oxidant tert-butyl hydroperoxide (THBP) (Abcam) was used as a positive control. 55 mM TBHP stock solution in water was diluted in complete media with 10% FBS without phenol red and also used straight away. To carry out the assay, SHSY5Y cells were seeded in a dark, clear-bottomed 96-well microplate to 50,000 cells per well and left to adhere overnight. Media was removed, after which the cells were washed twice using 100 μL/well media with 10% FBS without phenol red. After the removal of media, cells were stained by adding 100 μL/well of 20 µM DCFDA (prepared as above) followed by incubation for 45 min at 37 °C in the dark. DCFDA was removed after which cells were washed with 100 μL/well of complete media with 10% FBS without phenol red. Media was removed, after which 100 µl of 40 µg/ml oligomer alone or with 150 µg/ml antibody was added and incubated at 37 °C. Every hour fluorescence was recorded on a Varioskan fluorescence plate reader (Molecular Devices, San Jose, CA) at Ex/Em = 494/535 nm in end point mode. Blank readings were subtracted from all measurements.

### Animal models of PD

Adult female Sprague Dawley rats (225–250 g at the time of surgery) were housed 2-3 per cage and kept on a 12-hrs light/dark cycle with *ad libitum* access to food and water. All experiments were conducted in accordance with the ethical commitments for the use of laboratory animals at Aarhus University and approved by the Danish Supervisory Authority on Animal Testing. Two different approaches to achieve α-syn pathology in the rat brain were used. (1) Unilateral injections of recombinant adeno-associated viruses (rAAV5, 9.5*10^12 genome copies/ml; Michael J. Fox Foundation), encoding human α-syn under control of chicken-β-actin promoter into the ventral midbrain of the rats as performed before^61^. (2) injections of murine α-syn pre-formed fibrils (PFF m-α-syn) in rats, as described in ref. ^62^. The AAV-h-α-syn-injected animals were sacrificed 4-8 weeks post-surgery, and the α-syn PFF injected animals 22 weeks post-injection. Rats received an overdose of pentobarbital i.p. (50 mg/kg) and, upon cardiac arrest, were transcardially perfused through the ascending aorta with 0.9% saline, followed by 4% paraformaldehyde (PFA). Brains were removed and post-fixed for 2–4 h in 4% PFA and transferred to 0.25% sucrose (Fisher Scientific) solution for cryoprotection. Slicing of the brains was performed on a freezing microtome, and serial coronal sections of 40 µm thickness were collected in an antifreeze solution and stored at −20 °C.

### Preparation of brain lysates for ELISA experiments

Human A53T transgenic (line M83) mice (C57Bl/C3H background, expressing human α-syn under the control of the mouse prion protein gene promoter)^91^ were housed with *ad libitum* access to food and water, in a climate-controlled facility under a 12 h/12 h night/daylight cycle. In this study, five-months (homozygous ^+/+^, male, *n* = 1) and six-months (heterozygous ^+/−^, females, *n* = 3) old mice were used. Mice were terminated by cervical dislocation, the brain was extracted, and each hemisphere was cut into 3 coronal pieces (cut at approx. 1.70 mm and -2.92mm A-P from Bregma) and snap frozen on dry ice. The middle of the right hemisphere was homogenized according to Reimer et al.^92^ with modifications. Briefly, the brain was dounce-homogenized 1:10 in PBS containing protease- and phosphatase-inhibitor (Thermo Fisher Scientific) 20 bumps with rotor set at 700 rpm. The homogenates were sonicated (4×10 strokes using a Branson Ultrasonics Sonifier S-250 A, duty cycle 80, and output control 6). Indirect ELISA assays using homogenates diluted to different extents in PBS were carried out as described above, using as primary antibodies either the MJF14 mAb or 5 specified antibodies.

### Immunohistochemistry on rat brain tissue and microscopy

Immunohistochemistry was performed on free-floating sections. For the AAV-h-α-syn rat model, striatal (STR) and nigral (SN) sections were used, and prefrontal cortex sections from the PFF m-α-syn rat model. The concentration of the mAbs raised in this study was selected based on the optimal ELISA concentration (i.e. the minimal Ab concentration needed to elicit a read-out of ~1.5 OD_450_ in the indirect ELISA assay at an αSO concentration of 1 µg/ml) (Table S6). Between incubation steps, sections were rinsed in potassium-phosphate buffer (KPBS). Briefly, the sections were quenched for 10 min in a solution of 3% hydrogen peroxide and 10% methanol in KPBS, followed by blocking using the appropriate 5% serum in 0.25% Triton-X-100 in KPBS. The selected primary mAb was added in 2.5% serum in 0.25% Triton-X-100 in KPBS, and sections were left for overnight incubation at RT. Thereafter, sections were pre-incubated for 10 min in 1% serum in 0.25% Triton-X-100 in KPBS and incubated for 2 h with a biotinylated anti-mouse secondary antibody (1:200, Vector Laboratories) in 1% serum in 0.25% Triton-X-100 in KPBS. This was followed by a 1 hr incubation with avidin-biotin-peroxidase complex (Vectorstain, ABC kit, Vector Laboratories; PK-6100) in KPBS. Visualization was performed using 3,3-diaminobenzidine (DAB) and 0.1% hydrogen peroxide, both in KPBS, and sections were mounted on gelatine-coated glass slides and cover-slipped.

For immunofluorescence, three adjacent striatal sections from three different animals of the AAV-h-α-syn rat model, all showing prominent pathology, were used for each antibody staining. In addition to the selected mAbs, the sections were co-stained with one of two commercial antibodies; anti-aggregated α-syn MJFR-14-6-4-2 (MJF14, 1:10000, ab209538, Abcam, binds both fibrils and αSOs) or anti-phosphorylated α-syn at serine 129 (pSer129, 1:1000, ab23706S, Cell Signaling Technologies). Briefly, the sections were blocked in the appropriate 5% serum in 0.25% Triton-X-100 in KPBS, followed by overnight incubation with the selected primary antibodies in 2.5% serum in 0.25% Triton-X-100 in KPBS. The following day, sections were washed with KPBS, pre-blocked for 10 min in 1% serum in 0.25% Triton-X-100 in KPBS and incubated for 2 h in the dark with a species-specific fluorochrome-conjugated antibody (for in-house mAbs we used Alexa Fluor 488-conjugated goat anti-mouse, 1:400, A11029 Thermo Scientific, Waltham, MA; for commercial Abs we used Alexa Fluor 647-conjugated goat anti-rabbit IgG, 1:400, A21245, Invitrogen, Carlsbad, CA) and DAPI (1:2000, Sigma-Aldrich A/S, St. Louis, MO) in 1% serum in 0.25% Triton-X-100 in KPBS. Sections were mounted on gelatin-coated slides with Dako fluorescence mounting medium (S3023, Agilent Technologies, Santa Clara, CA).

Confocal images were obtained using a LSM 780/Axio Observer.Z1m using a 20x/0.8 M27 objective. Image analysis was performed using ImageJ (Fiji) on images obtained of the dorsal-ventral striatum due to the high levels of pathological accumulation of the h-α-syn in this region^61^. Extraction of single z-frame and maximum intensity projections were performed, and Pearson’s coefficient for co-localization was calculated using the JaCoP plugin in ImageJ. The thresholds used were: 9 for pSer129, 10 for MJF14, and between 7 and 10 for the mAbs (the same threshold was used for each mAb for all images to minimize subjectivity). The area covered and the overlap of the mAbs with the commercial antibodies and vice versa were calculated using the ImageJ software. Single z-frame images were filtered for duplicates and converted to an 8-bit greyscale. The Otsu automatic threshold was applied, with adjustment of the lower threshold due to background. A selection was created and added to the Region of Interest (ROI) manager for each image, and the overlap was identified by creating a shared ROI between the images. The area, area fraction (% area), and integrated density were calculated, and the percentage of overlap for each image was calculated as:

4 $$\% {\rm{overlap}}=100* {area\; of\; overlap\; region}/{area\; ofantibody\; ROI}$$

These values are provided in Table 4, where the overlap area is in column F and the areas of the antibodies (mAbs and commercial antibodies) are provided in columns D and E, respectively.

The following scores were applied according to each measurement within the co-stained sections (Table 4). For areas covered by our mAbs staining within each co-staining (Table 1, column D) high (pSer129/mAb >1000; MJF14/mAb >500, indicated by green color), medium (pSer129/mAb 500-1000; MJF14/mAb 300–500, indicated by yellow color), or low (pSer129/mAb <500; MJF14/mAb <300, indicated by red color). Areas overlapped within each co-staining (Table 4, column F): high (pSer129/mAb >150; MJF14/mAb >200, indicated by green color), medium (pSer129/mAb 90-150; MJF14/mAb 100-200, indicated by yellow color), or low (pSer129/mAb <90; MJF14/mAb <100, indicated by red color). Pearson co-localization for both co-stainings (Table 4, column C): high >0.450 (green color), medium 0.400–0.450 (yellow color), low 0.200–0.400 (red color) and very low <0.200 (dark red color). Percentage of overlap of the commercial antibody with our mAbs (Table 4, column G): very high >45% (green color), high 30–45% (yellow color), medium 20–30% (red color), low 10-20% (dark red color), and very low 0–10% (black color). These are color-coded in Table 4 for ease of reference.

#### Pre-adsorption of selected mAbs with monomeric α-syn

Immunohistochemistry was performed as described above on free-floating striatal and hippocampal sections from the AAV-h-α-syn rat model. Five selected mAbs, 10-9C8-C1, 14-9E7-A1, 17-9D12-A1, 18-3A5-H2, and 20-9E2-B8, were used as primary antibodies, and a commercial antibody was used for comparison, namely anti-aggregated α-syn MJFR14-6-4-2 (MJF14, 1:10000, ab209538, Abcam). The secondary antibodies used were biotinylated anti-rabbit and biotinylated anti-mouse (both 1:200, from Vector Laboratories, Newark, CA). Prior to adding the primary antibodies, they were incubated 1:1 with human monomeric α-syn (1 mg/mL) for 1 h at RT. The antibody-α-syn mix was then added to 30 kDa Amicon-Ultra 0.5 mL 30 K cut-off spin filters (MilliporeSigma, Burlington, MA), and KPBS added to a final volume of 500 µl, centrifuged 1 min at 10.000 rpm at RT, leaving 100 µl fluid containing the antibody in the filter. Protein concentration was measured using a NanoDrop (Thermo Fisher Scientific, Waltham, MA) to ensure the right concentration of the respective antibody was added to the sections. The antibody in the filter was then resuspended and added to a mixture of 2.5% serum in 0.25% triton in KBPS and added to the sections for over-night incubation at RT, and the protocol continued as described above.

### Immunohistochemistry on *post-mortem* human brain tissue and microscopy

Formalin-fixed paraffin-embedded (FFPE) post-mortem brain samples from patients with PD (*n* = 4), DLB (*n* = 2), MSA (*n* = 5), and controls (*n* = 4) were used in the study. The brains were donated to the Brain Bank at Bispebjerg-Frederiksberg Hospital (Copenhagen University Hospital, Denmark; approved by the Danish Data Protection Agency, BFH-2017-001, I-suite 05190, and the Regional Ethical Committee (Capital Region Denmark) j.nr. H-16037525), and all donated brains had previously been examined to verify the diagnosis (Table S7). From each brain, 10 µm FFPE sections from the SN and frontal cortex were mounted on SuperFrost slides and stained by immunohistochemistry. Five selected mAbs (10-9C8-C1 (1:300), 14-9E7-A1 (1:900), 17-9D12-A1 (1:1600), 18-3A5-H2 (1:300), and 20-9E2-B8 (1:250)) were used as primary antibodies, and an anti-human-α-syn antibody 4B12 (1:2000, GTX21904, GeneTex), which recognizes human α-syn, was used as a positive control. Negative controls omitted the primary antibody. Briefly, paraffin was removed using xylene, decreasing ethanol dilutions and water, followed by incubation for 10 min in 3% hydrogen peroxide (Brintoverilte 3%, 221378, Apoteket Hovedstaden, Copenhagen) and washed for 5 min in water. Paraffin removal and antigen retrieval was performed with TEG buffer pH 9 (Tris 10 mmol/l, EGTA 0.5 mmol/l, Apoteket Hovedstaden, v.nr. 862338, Copenhagen) followed by 2×5 min wash with PBS. Slides were incubated for 1 h at room temperature with the appropriate primary antibody in PBS (200 µl/slide) in a humidity chamber. Slides were washed in PBS followed by 30 min incubation with the secondary antibody (Anti-mouse, EnVision+ System – HRP labeled polymer, #K4001, Dako, Glostrup, Denmark). Sections were washed with PBS, and development was performed with DAB and hydrogen peroxide (BrightDAB solution, lot numbers VWRKBS04-500 or MFCD00007725, Immunologic, Arnhem, The Netherlands). Slides were counterstained with hematoxylin (Mayers, Dako Glostrup, Denmark) before coverslipping with Pertex (00801, HistoLab, Gothenburg, Sweden).

Sections were analyzed for positive staining and pathology using a 20x, 40x, and 63x (oil) objective. Images were obtained using a bright-field Leica DM600B (Prior Scientific) microscope with the VIS software (Visiopharm, Hørsholm, Denmark). The anatomical regions analyzed were the substantia nigra (SN) and the white matter of the frontal cortex (WM CTX). The ROI was drawn in the VIS software using a 1.25x lens, and a 20x and 40x objective was used to access and evaluate positive staining, by giving a qualitative score based on the density of staining of the different morphologies; − (0, absent), + (1-25, mild), ++ (25-50, moderate) and +++ (>50, frequent). The morphologies analyzed were healthy pigmented neurons, neuropil “grains”, LB and LN, extracellular inclusion bodies, and GCIs. For pigmented neurons in the SN, +++ indicates no notable degeneration while + indicates severe degeneration.

### Supplementary information

Supplementary Material

## Data Availability

The datasets obtained and analysed during the current study are available from the corresponding authors (D.E.O. for biophysical data, M.R.R. for IHC data) on request.
