# Supplementary material for: Molecular properties and diagnostic potential of monoclonal antibodies targeting cytotoxic α-synuclein oligomers
Source: NPJ Parkinsons Dis. 2024 Jul 29;10:139. doi: 10.1038/s41531-024-00747-6 (PMC11286781; doi:10.1038/s41531-024-00747-6)
Supplement: Supplementary file 1 — Supplementary Material [file 41531_2024_747_MOESM1_ESM.pdf]

Monoclonal antibodies targeting cytotoxic α-synuclein oligomers: molecular properties and diagnostic potential

Janni Nielsen, Johanne Lauritsen, Jannik N. Pedersen, Jan S. Nowak, Malthe K. Bendtsen, Giulia Kleijwegt, Kaija Lusser, Laia C. Pitarch, Julián V. Moreno, Matthias M. Schneider, Georg Krainer, Louise Goksøyr, Paul Khalifé, Sanne Simone Kaalund, Susana Aznar, Magnus Kjærgaard, Vita Sereikaité, Kristian Strømgaard, Tuomas P.J. Knowles, Morten Agertoug Nielsen, Adam F. Sander, Marina Romero-Ramos, Daniel E. Otzen

Supplementary Information

Supplementary Tables S1-S7

Supplementary Figures S1-S8

Supplementary Tables

Table S1. Quantification of human pathology with mAb 10-9C8-C1

Disease	ID	Area	Pigmented neurons	Extracellular inclusions	LBs	LNs	Neuropil "grains"	GCI
CTRL	117	SN	+++	-	-	-	-	-
		WM CTX		-	-	-	-	-
CTRL	384	SN	+++	-	-	-	-	-
		WM CTX		-	-	-	-	-
CTRL	127	SN	++/+++	++	+	++/+++	++/+++	-
		WM CTX		-	-	-	-	-
CTRL	287	SN	+++	-	-	-	-	-
		WM CTX		-	-	-	-	-
PD	684	SN	+	-	-	+	+	-
		WM CTX		-	-	-	-	-
PD	337	SN	- / +	-	- / +	-	- / +	-
		WM CTX		-	-	-	-	- / +
PD	248	SN	++	- / +	+/++	+	+/++	-
		WM CTX		-	-	-	- / +	-
PD	191	SN	+	+++	+++	+++	+++	- / +
		WM CTX		+/++	+	+	+/++	-
DLB	256	SN	+/++	+++	+++	+++	+++	- / +
		WM CTX		+	+	+	++	- / +
DLB	543	SN	+/++	++/+++	++	++	+++	- / +
		WM CTX		-	+	-	- / +	-
MSA	477	SN	+	-	-	-	-	++
		WM CTX		-	-	-	-	++/+++
MSA	578	SN	+	+	-	+	+	+++
		WM CTX		- / +	-	-	+	++
MSA	167	SN	++	+	- / +	-	-	+++
		WM CTX		+	-	+	+	+++
MSA	623	SN	+	++	+/++	+++	++/+++	+++
		WM CTX		+	- / +	+	+/++	++
MSA	312	SN	- / +	+	+	+	+	+++
		WM CTX		-	-	-	+	++

Table S2. Quantification of human pathology with mAb 14-9E7-A1

Disease	ID	Area	Pigmented neurons	Extracellular inclusions	LBs	LNs	Neuropil "grains"	GCI
CTRL	117	SN	+++	-	-	-	-	-
		WM CTX		-	-	-	-	-
CTRL	384	SN	+++	+	-	- / +	+	-
		WM CTX		-	-	-	+/++	-
CTRL	127	SN	++/+++	++	++	++/+++	++/+++	-
		WM CTX		-	-	-	-	-
CTRL	287	SN	+++	-	-	-	-	-
		WM CTX		-	-	-	-	-
PD	684	SN	+	- / +	+	+/++	++	-
		WM CTX		-	-	-	-	-
PD	337	SN	NA	NA	NA	NA	NA	NA
		WM CTX		-	-	-	-	- / +
PD	248	SN	++	+	+/++	++	++	-
		WM CTX		-	-	+	- / +	-
PD	191	SN	+	+++	+++	+++	+++	- / +
		WM CTX		+	+	+	++/+++	- / +
DLB	256	SN	+/++	+++	+++	+++	+++	- / +
		WM CTX		+/++	+	+/++	+++	- / +
DLB	543	SN	+	++	++/+++	++	+++	- / +
		WM CTX		-	-	-	- / +	-
MSA	477	SN	+	-	-	-	-	+
		WM CTX		-	-	-	-	++/+++
MSA	578	SN	+	+	-	+	+	+++
		WM CTX		+	-	-	+	++
MSA	167	SN	++	+	- / +	-	+	+++
		WM CTX		+	-	+	+	+++
MSA	623	SN	+	++/+++	++	+++	++/+++	+++
		WM CTX		+	+	++	++/+++	++
MSA	312	SN	- / +	+	+	+	++	+++
		WM CTX		-	-	-	+	++

Table S3. Quantification of human pathology with mAb 17-9D12-A1

Disease	ID	Area	Pigmented neurons	Extracellular inclusions	LBs	LNs	Neuropil "grains"	GCI
CTRL	117	SN	+++	-	-	-	-	-
		WM CTX		-	-	-	-	-
CTRL	384	SN	+++	-	-	-	-	-
		WM CTX		-	-	-	+	-
CTRL	127	SN	++/+++	+	+	+/++	++/+++	-
		WM CTX		-	-	-	-	-
CTRL	287	SN	+++	-	-	-	-	-
		WM CTX		-	-	-	-	-
PD	684	SN	+	-	-	-	+	-
		WM CTX		-	-	-	-	-
PD	337	SN	- / +	-	-	-	+	-
		WM CTX		-	-	-	-	- / +
PD	248	SN	++	-	+	+	+	-
		WM CTX		-	-	-	-	-
PD	191	SN	+	++/+++	++/+++	++	++/+++	- / +
		WM CTX		+	+	+	++	-
DLB	256	SN	+/++	++/+++	++/+++	++	++	- / +
		WM CTX		+	+	+	++	- / +
DLB	543	SN	+	+	+	+	+/++	-
		WM CTX		-	-	-	-	-
MSA	477	SN	+	-	-	-	-	-
		WM CTX		-	-	-	-	+
MSA	578	SN	+	-	-	+	+	+++
		WM CTX		-	-	-	+	+/++
MSA	167	SN	++	+	- / +	- / +	+	++/+++
		WM CTX		-	-	-	+	++/+++
MSA	623	SN	+	+	+/++	++	++/+++	++/+++
		WM CTX		-	-	+	+	+/++
MSA	312	SN	- / +	+	- / +	-	+/++	+++
		WM CTX		-	-	-	+	- / +

Table S4. Quantification of human pathology with mAb 18-3A5-H2

Disease	ID	Area	Pigmented neurons	Extracellular inclusions	LBs	LNs	Neuropil “grains”	GCI
CTRL	117	SN	+++	-	-	-	-	-
		WM CTX		-	-	-	-	-
CTRL	384	SN	+++	-	-	-	-	-
		WM CTX		-	-	-	-	-
CTRL	127	SN	++/+++	+	+	++/+++	+/++	-
		WM CTX		-	-	-	-	-
CTRL	287	SN	+++	-	-	-	-	-
		WM CTX		-	-	-	-	-
PD	684	SN	+	-	+	+	+	-
		WM CTX		-	- / +	-	-	-
PD	337	SN	- / +	-	-	-	-	-
		WM CTX		-	-	-	-	-
PD	248	SN	++	+	+	+	+	-
		WM CTX		-	-	-	-	-
PD	191	SN	+	+	+/++	- / +	+	-
		WM CTX		-	-	-	+	-
DLB	256	SN	+/++	+/++	+/++	+	+/++	-
		WM CTX		- / +	- / +	- / +	+	- / +
DLB	543	SN	+	+	+/++	+	+/++	- / +
		WM CTX		-	-	-	-	-
MSA	477	SN	+	-	-	-	-	+/++
		WM CTX		-	-	-	-	+
MSA	578	SN	+	-	-	+	-	++
		WM CTX		-	-	-	-	+
MSA	167	SN	+/++	- / +	-	-	-	++
		WM CTX		-	-	-	+	++/+++
MSA	623	SN	+	+	+/++	+++	+/++	++/+++
		WM CTX		+	- / +	-	+	+/++
MSA	312	SN	- / +	-	-	-	- / +	++
		WM CTX		-	-	-	-	-

Table S5. Quantification of human pathology with mAb 20-9E2-B8

Disease	ID	Area	Pigmented neurons	Extracellular inclusions	LBs	LNs	Neuropil “grains”	GCI
CTRL	117	SN	+++	-	-	-	-	-
		WM CTX		-	-	-	-	-
CTRL	384	SN	+++	+	+	+	+	-
		WM CTX		-	-	-	-	-
CTRL	127	SN	++/+++	+/++	+	++/+++	++/+++	-
		WM CTX		-	-	-	-	-
CTRL	287	SN	+++	-	-	-	-	-
		WM CTX		-	-	-	-	-
PD	684	SN	+	- / +	+	+	+/++	-
		WM CTX		-	-	- / +	+	-
PD	337	SN	- / +	-	-	-	- / +	-
		WM CTX		-	-	-	-	-
PD	248	SN	++/+++	+	+	++	++	-
		WM CTX		-	-	-	-	-
PD	191	SN	+	+++	+++	+++	+++	- / +
		WM CTX		+	+	+	+/++	-
DLB	256	SN	+/++	+++	+++	+++	+++	- / +
		WM CTX		+	+	+/++	++	- / +
DLB	543	SN	+	+++	++/+++	+++	+++	- / +
		WM CTX		-	-	+/++	++	-
MSA	477	SN	NA	NA	NA	NA	NA	NA
		WM CTX		-	-	-	-	+/++
MSA	578	SN	+	- / +	-	+/++	+	+++
		WM CTX		+	-	-	-	++
MSA	167	SN	++	+	- / +	+	+	+++
		WM CTX		+	-	+	+	+++
MSA	623	SN	+	++	+	+++	++	+++
		WM CTX		+	+	++	++	++
MSA	312	SN	- / +	+	+	- / +	- / +	+++
		WM CTX		-	-	-	-	+

Table S6. Concentrations of antibodies used for staining of rat striatal sections.

Ab ID	Stock concentration (mg/ml)	ELISA concentration (µg/ml)	ELISA x 20 (mg/ml)	Volume (µl/ml) $\left(\frac{ELISA\ x\ 20}{Stock\ conc.}\ x\ 1000\right)$
**3-9H9**	0.42	1	0.02	47.62
**6-3A10-A1**	0.28	1	0.02	71.43
**8-9H4-E3**	0.67	2	0.04	59.70
**10-9C8-C1**	0.61	1	0.02	32.79
**13-3H6-D2**	0.55	1	0.02	36.36
**14-9E7-A1-**	0.9	0.5	0.01	11.11
**14-IE2-E1**	0.52	1.5	0.03	57.69
**16-9E2**	0.5	1	0.02	40.00
**17-5A8-H1-**	1.12	1.5	0.03	26.79
**17-9D12-A1**	1.59	0.5	0.01	6.29
**18-3A5-H2-**	2.05	1	0.02	9.76
**18-9E10-B1**	1.16	2	0.04	34.48
**19-2C3-F10**	0.9	0.5	0.01	11.11
**20-5H12-C10-**	0.69	2	0.04	57.97
**20-9E2-B8-**	0.77	1.5	0.03	38.96
**21-4G12-**	1.03	2	0.04	38.83

Table S7. Demographics of human patients

Clinical diagnosis	N	Gender (M/F)	Age of death	Age of disease onset	PMI (hrs)
**CTRL**	4	2/1?	82.3 ± 14.7	-	54 ± 36
**PD**	4	3/1	83.5 ± 11.5	74.8 ± 12.3	62 ± 28
**DLB**	2	2/0	70.5 ± 6.5	62.5 ± 11.5	86.5 ± 30.5
**MSA**	5	3/2	67.0 ± 11.0	60.0 ± 12.0	48.5 ± 23.5

PMI: post-mortem interval. CTRL: control. PD: Parkinsons Disease. MSA: Multiple system atrophy. DLB: Dementia with Lewy bodies.

Figure S1a

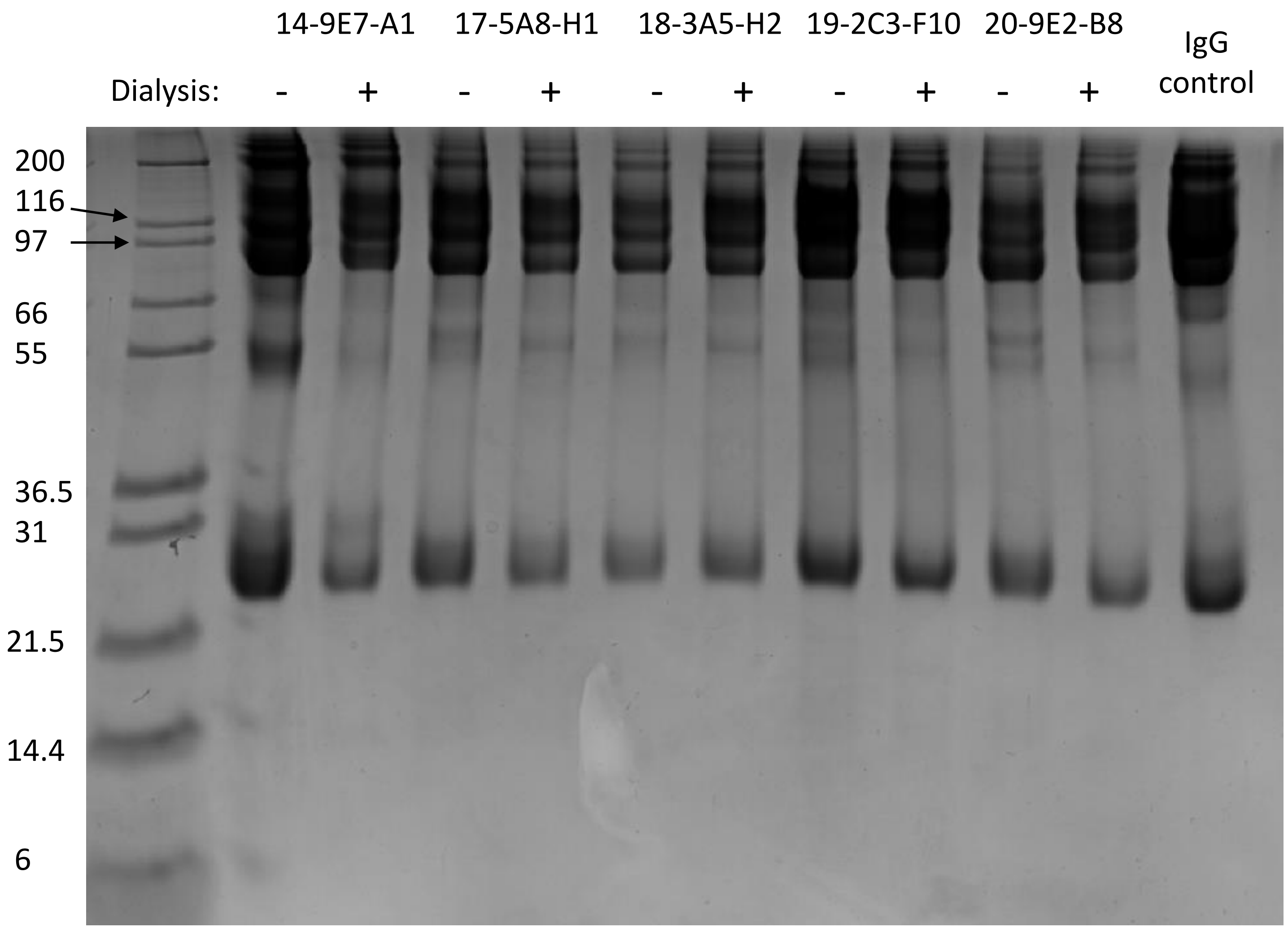

Figure S1a. SDS-PAGE of purified monoclonal antibodies before and after the final dialysis step against 1xPBS. A control sample of polyclonal IgG is shown furthest to the right.

Figure S1b

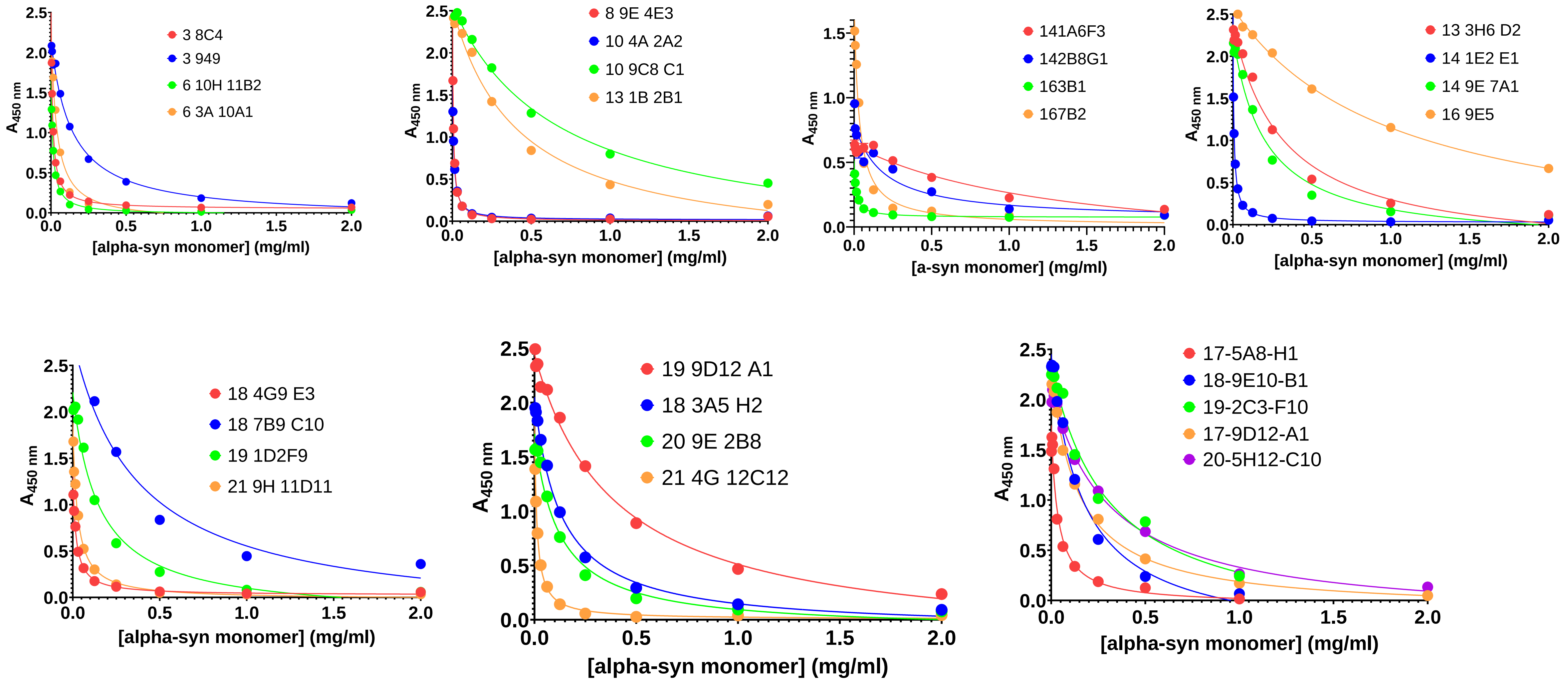

Figure S1b. Competition ELISA assays in which immobilized αSO is exposed to a fixed amount of mAb in the presence of 0-2 mg/ml monomeric α-syn in the soluble phase. The steeper the decline in mAb binding, the higher the relative affinity of the mAb for monomeric rather than oligomeric α-syn. Data summarized in **Table 2**.

Figure S2a

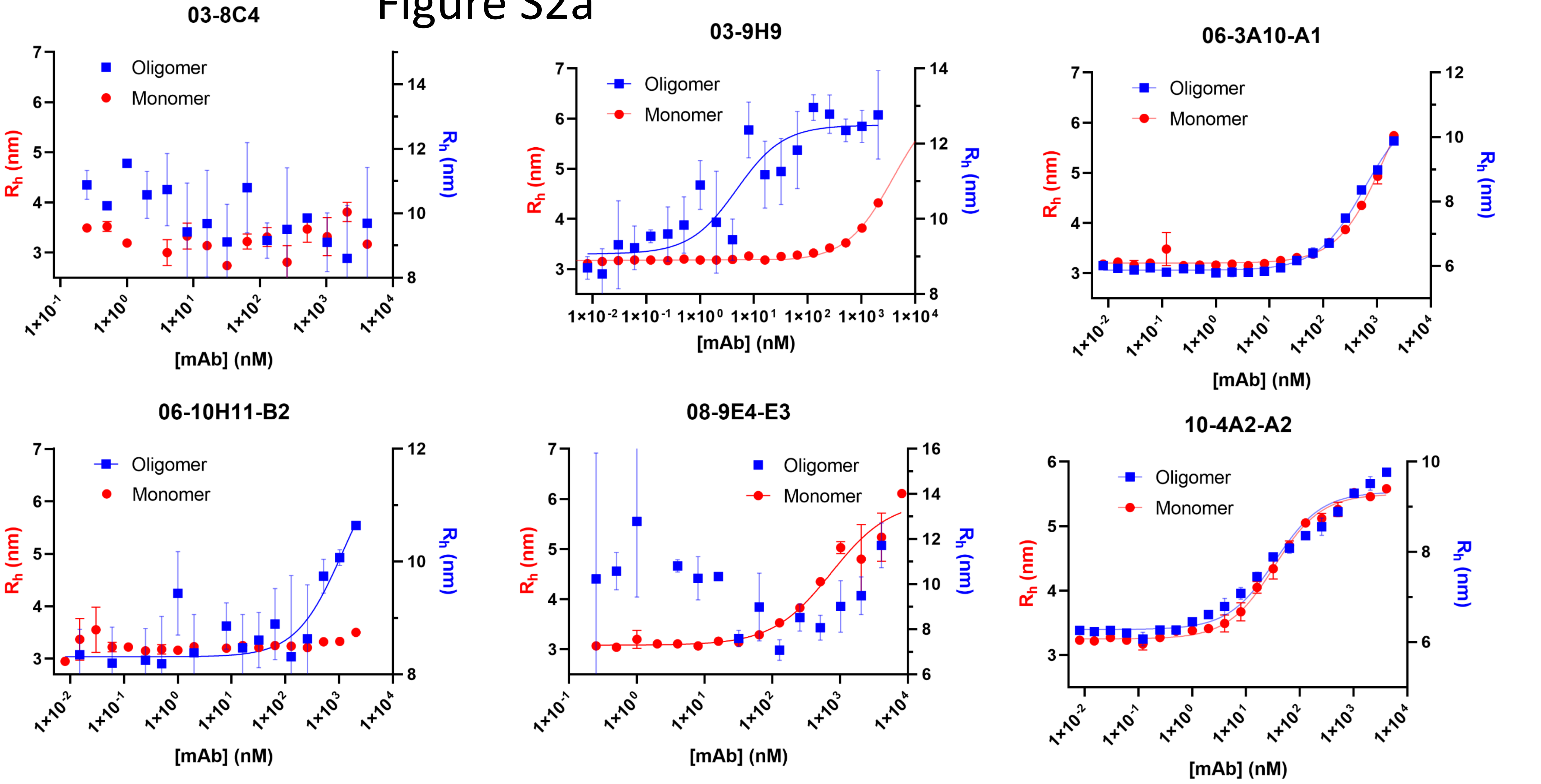

Figure S2a. FIDA plots for the affinity of mAbs for oligomeric and monomeric α-syn. Data summarized in Table 2, columns E-G. Errors from fits to individual FIDA curves.

Figure S2a continued

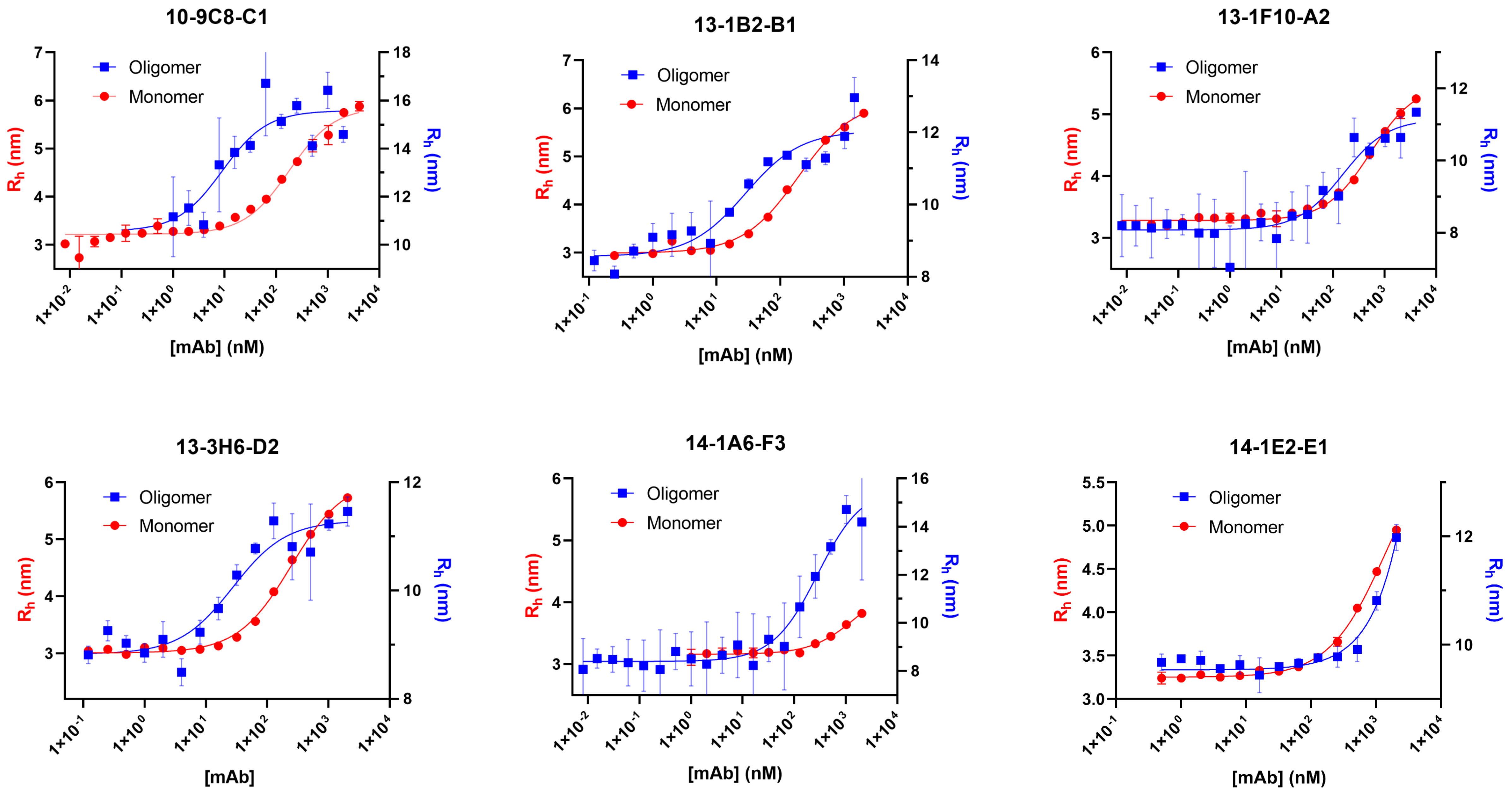

Figure S2a continued

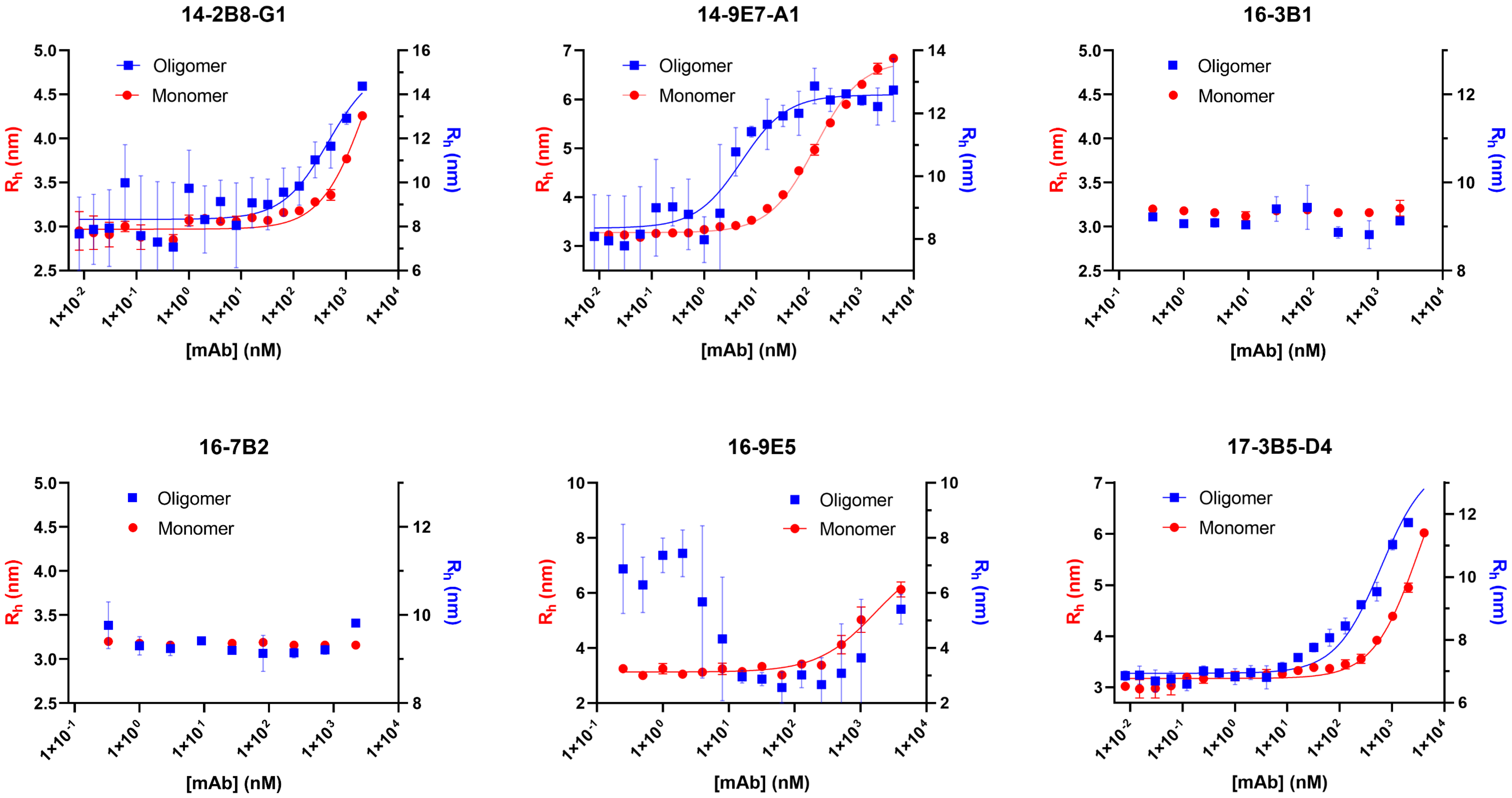

Figure S2a continued

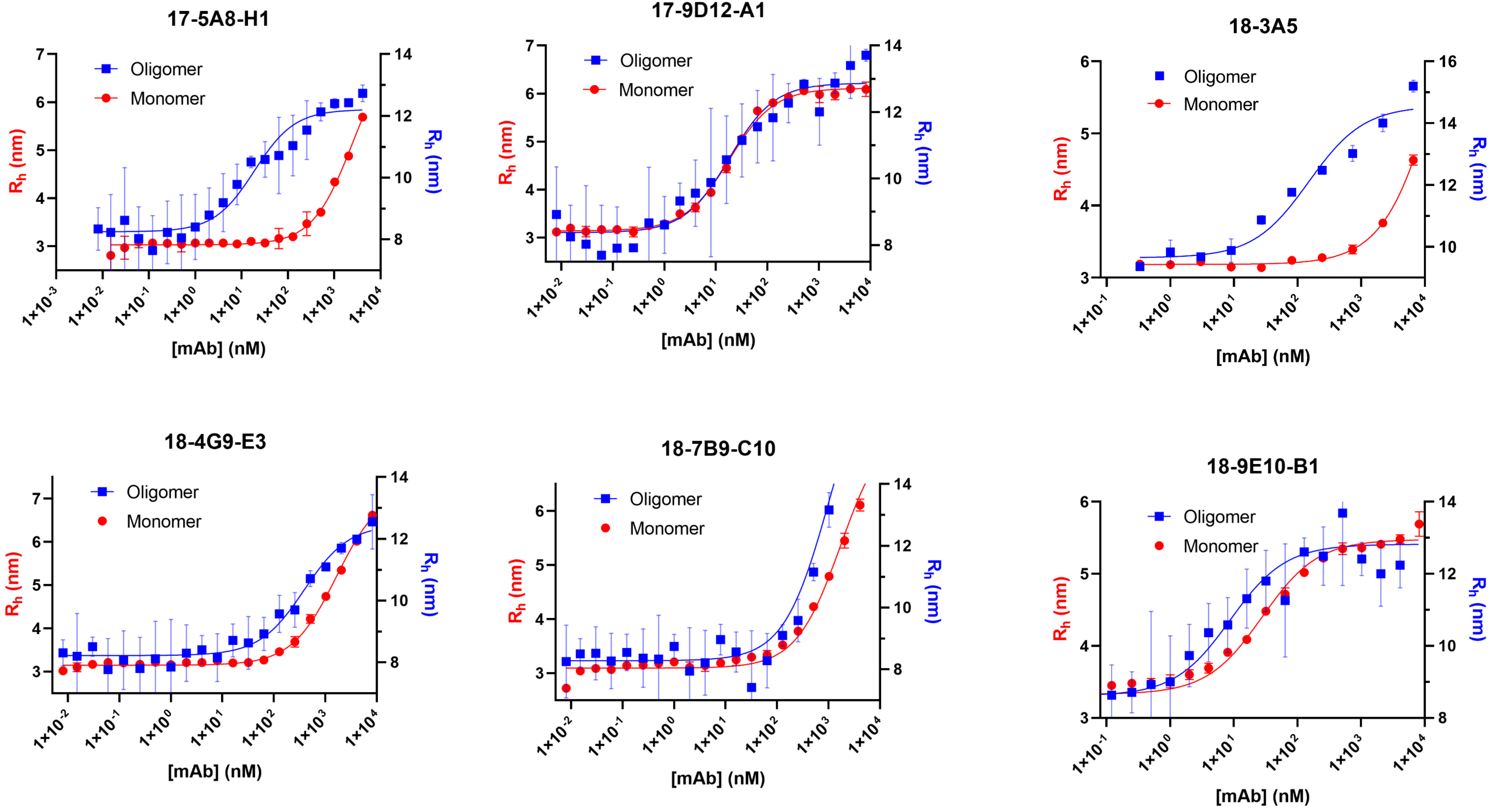

Figure S2a continued

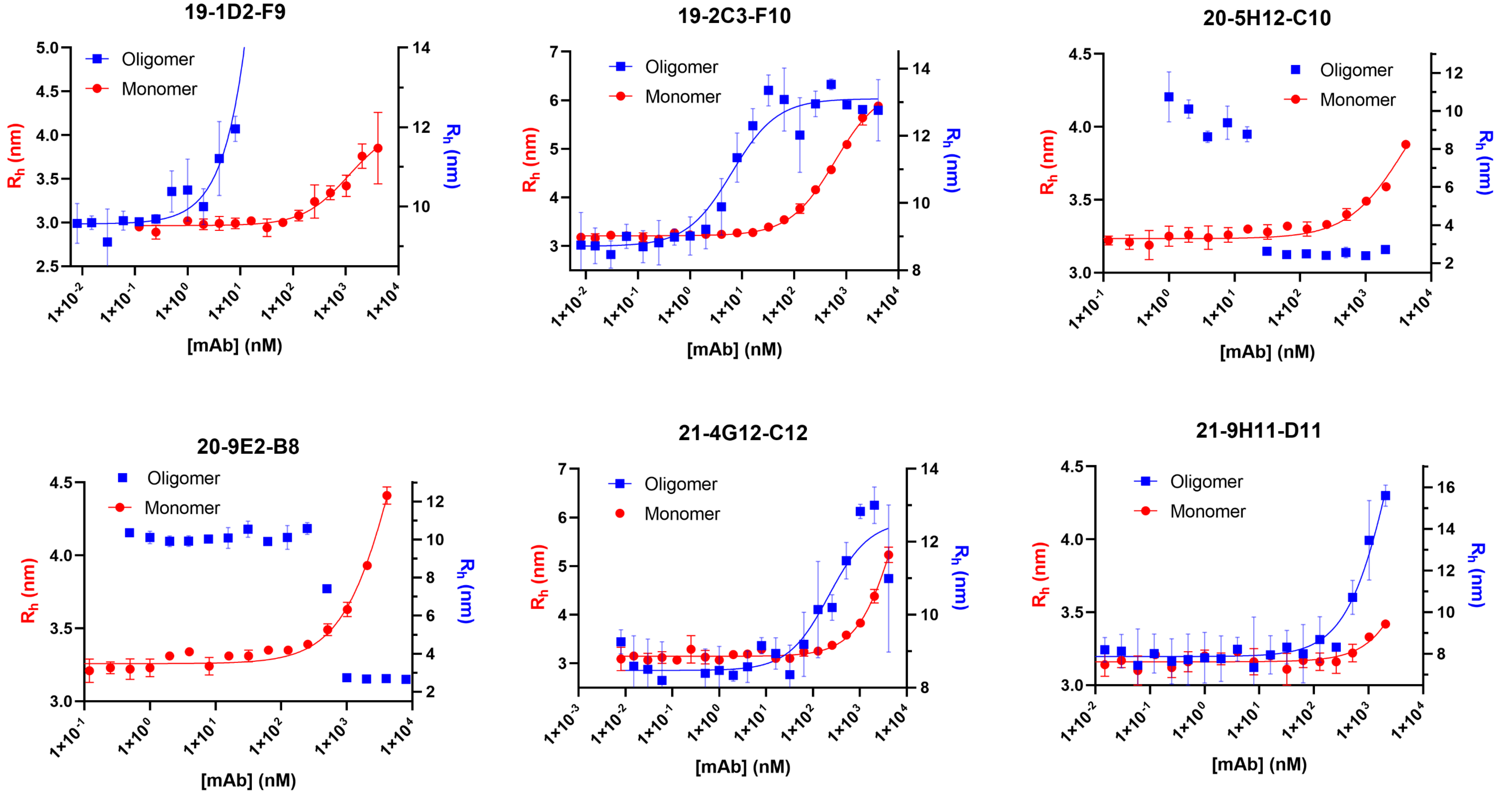

Figure S2b

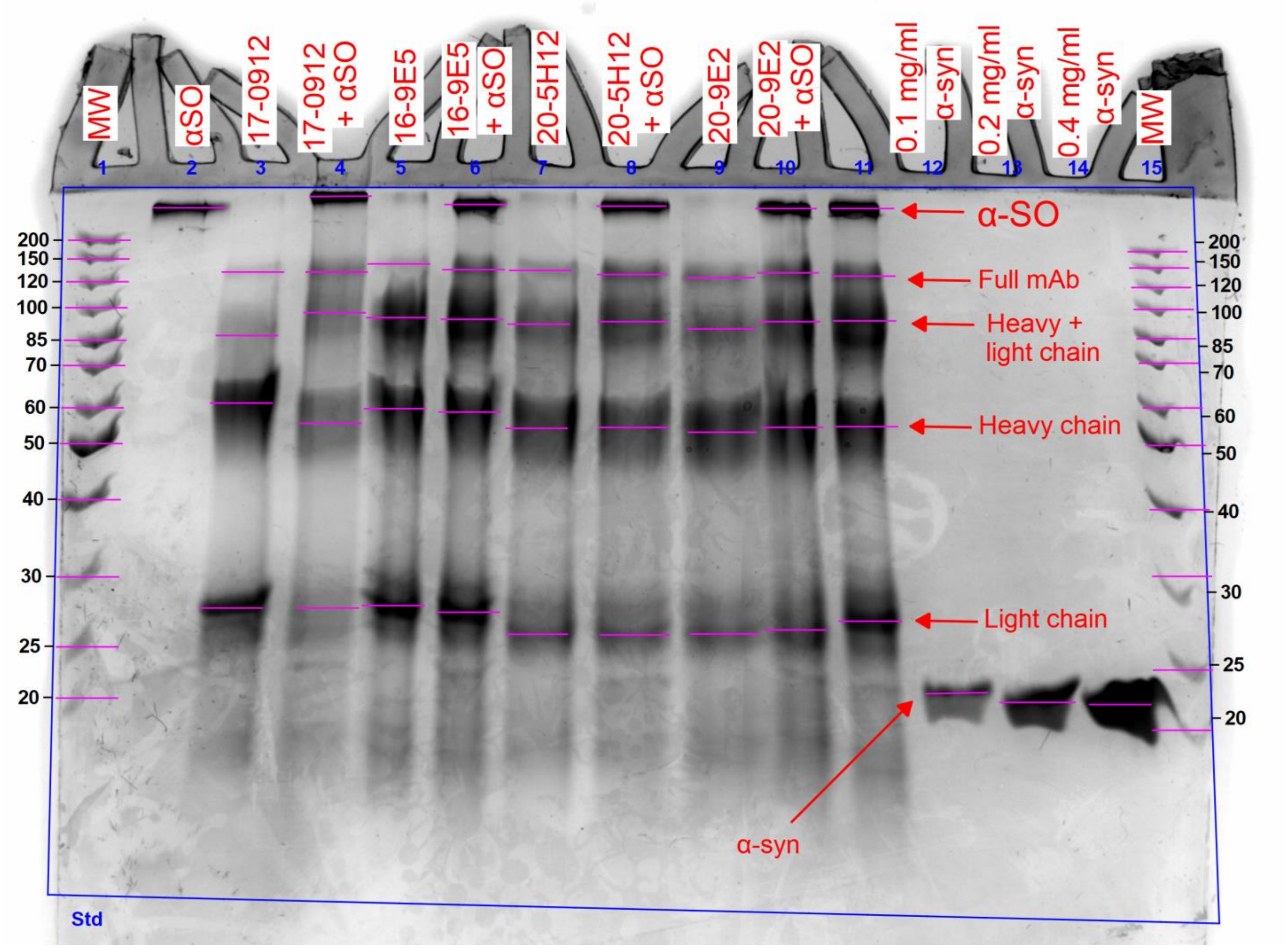

Figure S2b. SDS-PAGE of mAbs 17-9D12-A1 (control), 16-9E5, 20-5H12-C10, and 20-9E2-B8 incubated with and without αSO in lanes 3-10. Pure αSO is shown in lane 2 and monomeric α-syn in lanes 12-14. Lines drawn are meant to guide the eye.

Figure S2c

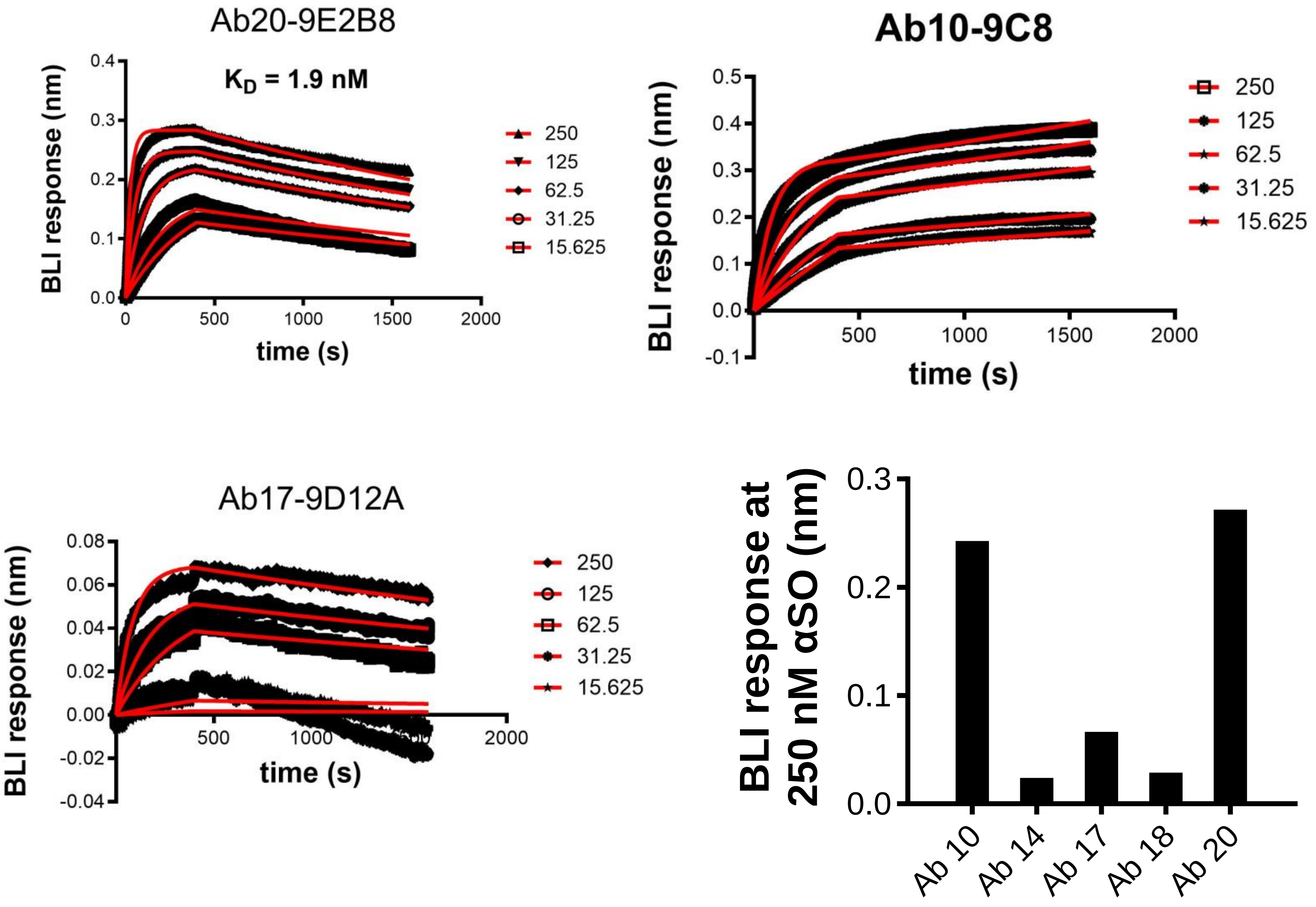

Fig. S2c. The binding affinity of several shortlisted mAbs was also evaluated by biolayer interferometry (BLI). However, the binding signal observed in BLI for some of these mAbs in the presence of the αSN oligomer was weak or not detectable, and titration at different oligomer concentrations yielded binding curves difficult to analyze. Therefore, it was not possible to determine a K_D for the mAbs tested except mAb 20-9E2B8, which showed a K_D of ~1.9 nM, in very good agreement with an SPR-based value of 1.5 nM (Table 2). These BLI results, together with previous unsuccessful attempts to characterize other molecular binders towards αSOs by BLI (data not shown), suggest that using the reported experimental setup, this technique is not suitable for assessing the binding of αSOs.

Figure S2d

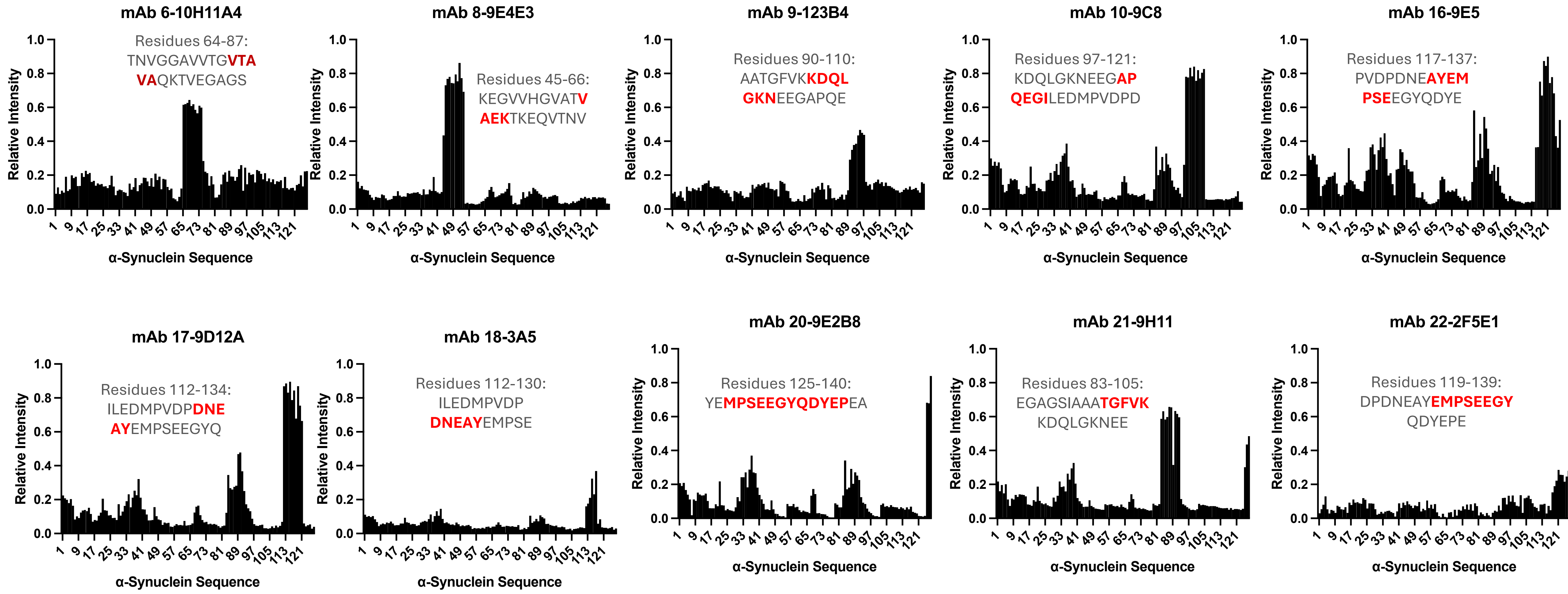

Figure S2d. Microarray-based identification of linear epitopes for mAbs raised against αSOs (extension of data shown in Fig. 3cde). Graphs show relative intensity versus the position of the first residue in a given 14-mer peptide in the α-syn sequence (data normalized to intensity for Ab 3-8C4 in Fig. 3c). Inserts indicate major sequence recognized by the mAb (residues in bold red indicate those residues present in all the peptides in the peak region). Note that mAbs 18-9E10B1 and 19-1D2 failed to show any significant binding to the peptide arrays (data not shown).

Figure S3a

Figure S3a

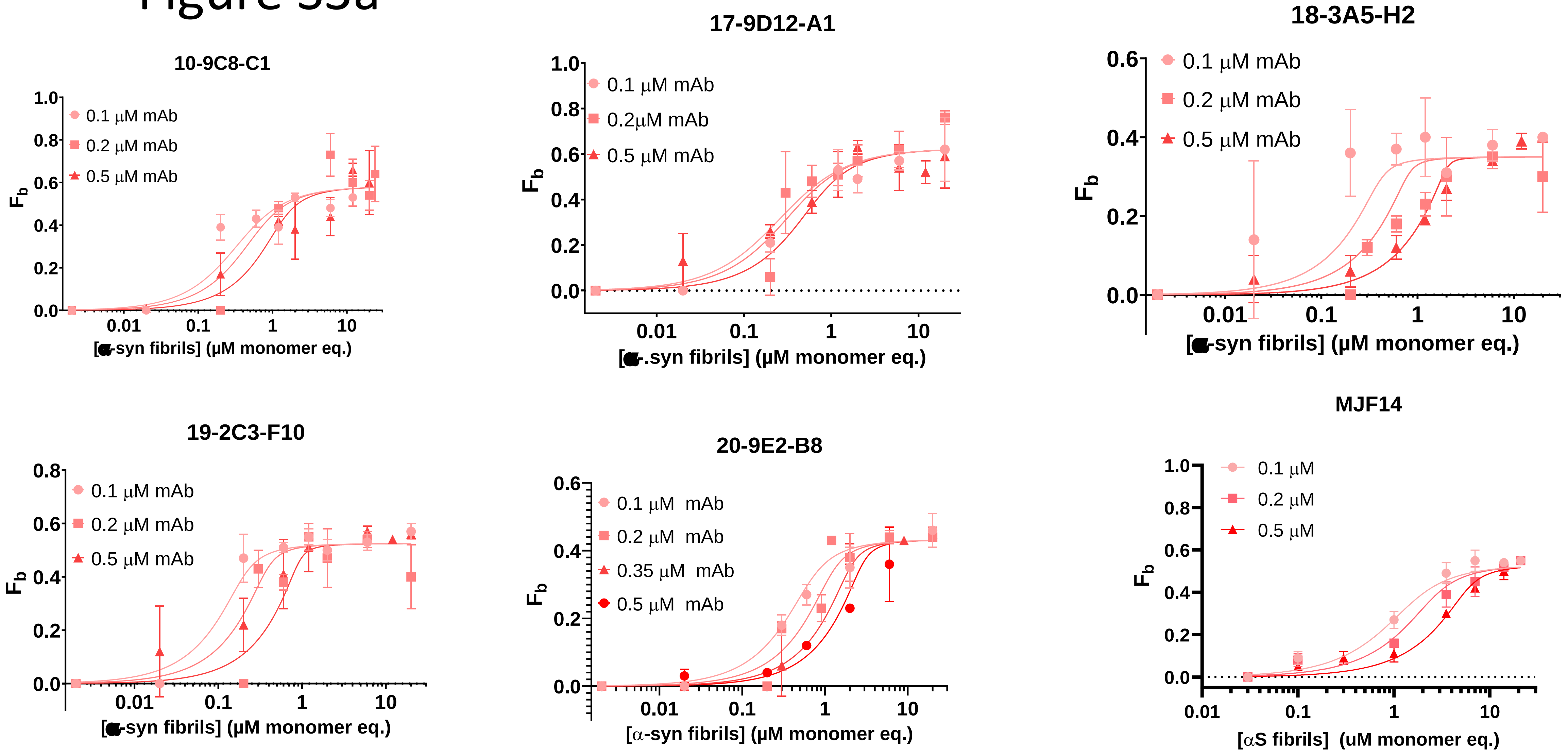

Figure S3a. Fibril-antibody binding curves obtained by microfluidic diffusional sizing (MDS). Errors from fits to individual MDS curves. Data analysed using equation 2.

Figure S3b

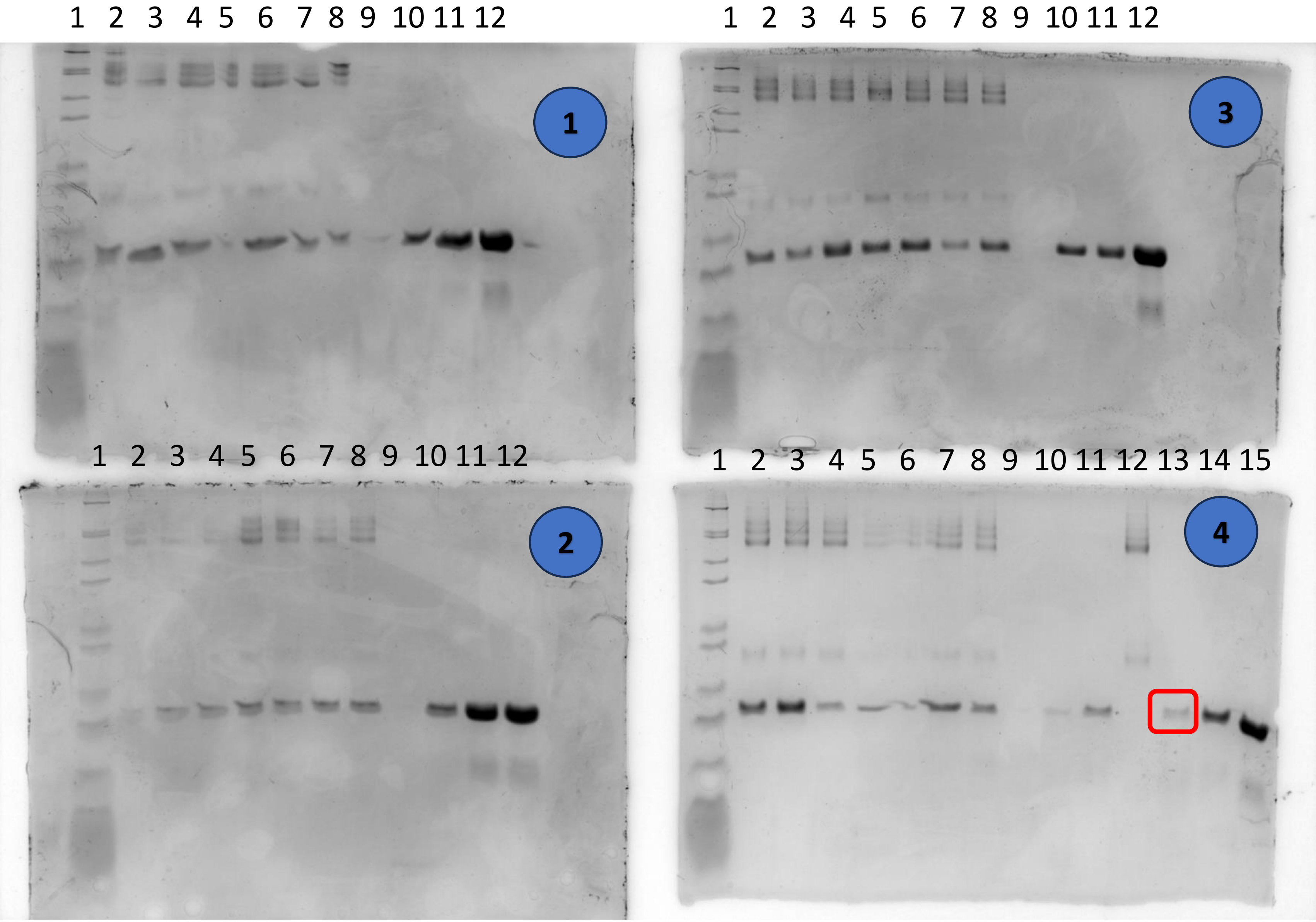

Lane	Gel1	Gel2	Gel3	Gel4
1	Mwt	Mwt	Mwt	Mwt
2	38C4	131B2	167B2	183A5
3	39H9	133H6	169E5	209E2
4	610H	141A6	184G9	214G1
5	63A1	142B8	187B9	A5A8
6	89E4	141E2	191D2	189E1
7	104A	1419E1	219H11	205H12
8	109C	163B1	179D12	192C3
9	-	-	-	-
10	0.1g/L	0.1g/L	0.1g/L	Mono
11	0.3g/L	0.3g/L	0.3g/L	M+S
12	0.5g/L	0.5 g/L	0.5 g/L	mAb
13	-	-	-	M + beads
14	-	-	-	0.3g/L
15	-	-	-	0.5g/L

Fig. S3b. SDS-PAGE analysis of the soluble fractions left from seeding experiments (Fig. 3b). Note the internal standards (0.1-0.3 mg/ml monomeric α-syn) and the very small amount of soluble α-syn left after seeded fibrillation in the absence of mAbs. 4 of the mAbs lead to retention of 30-40% of monomeric α-syn (see text).

Figure S3c

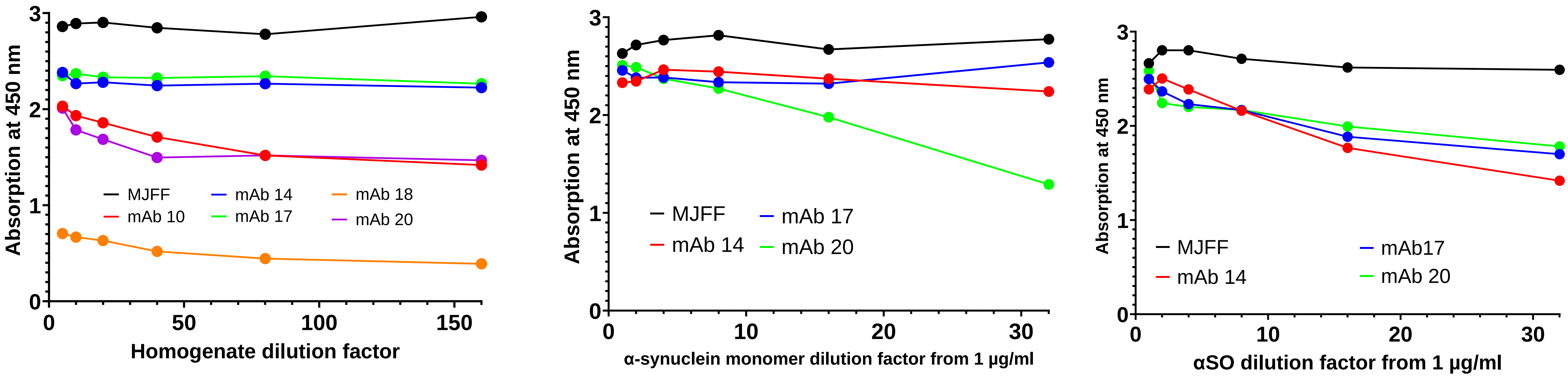

Fig. S3c. Indirect ELISA assays using immobilized brain lysates from a male PD rat. Left panel: Comparison of the binding of the MJF14 mAb with the 5 mAbs selected for further analysis (10-9C8-C1, 14-9E7-A1, 17-9D12-A1, 18-3A5-H2, and 20-9E2-B8). Antibodies indicated by their first two digits only. Middle and right panels: Binding of the indicated antibodies to immobilized monomeric α-syn (middle) and αSO (right).

Figure S4

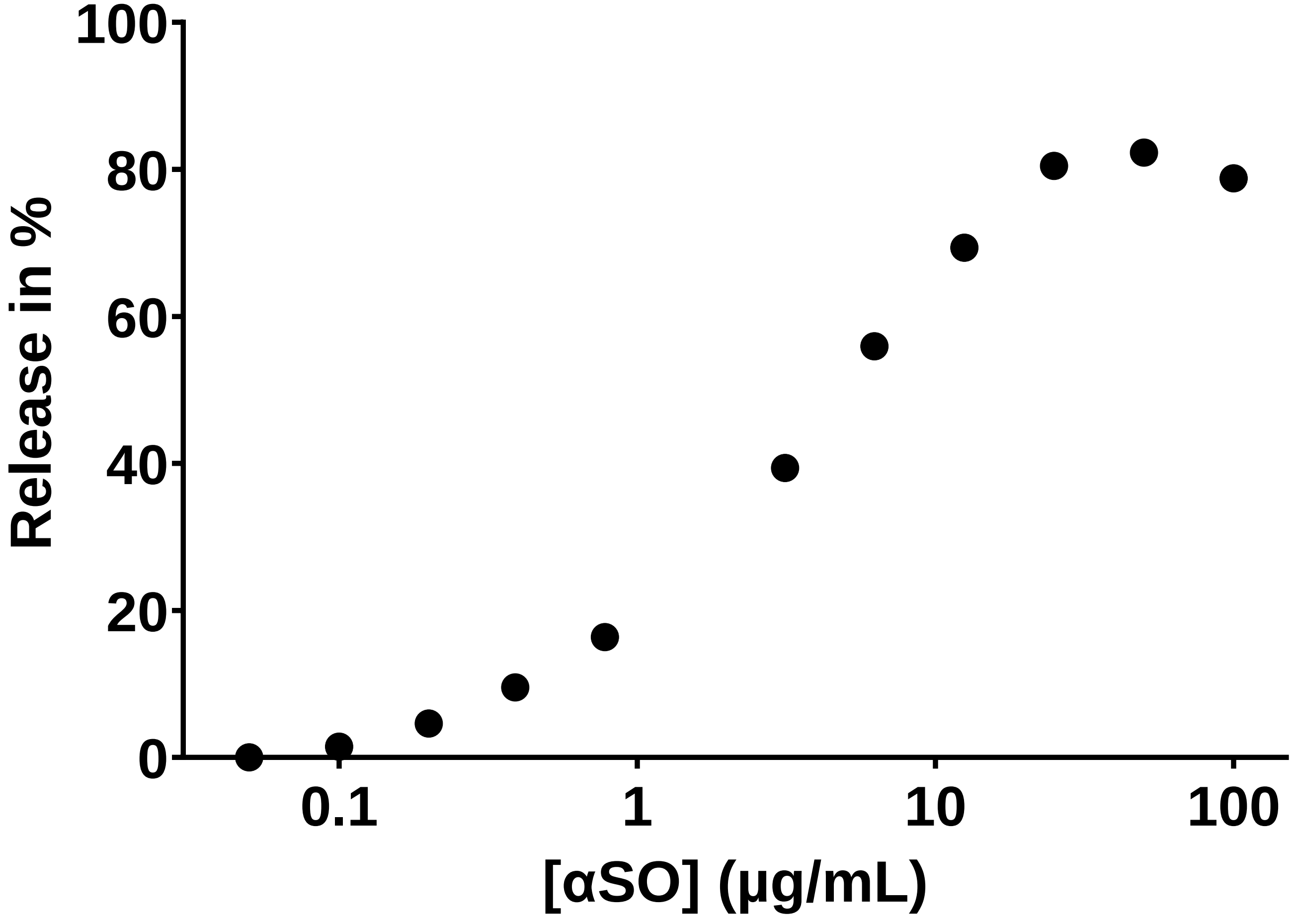

Figure S4. Calibration curve for release of calcein from DOPG vesicles by 0.05-100 µg/ml αSO.

Figure S5

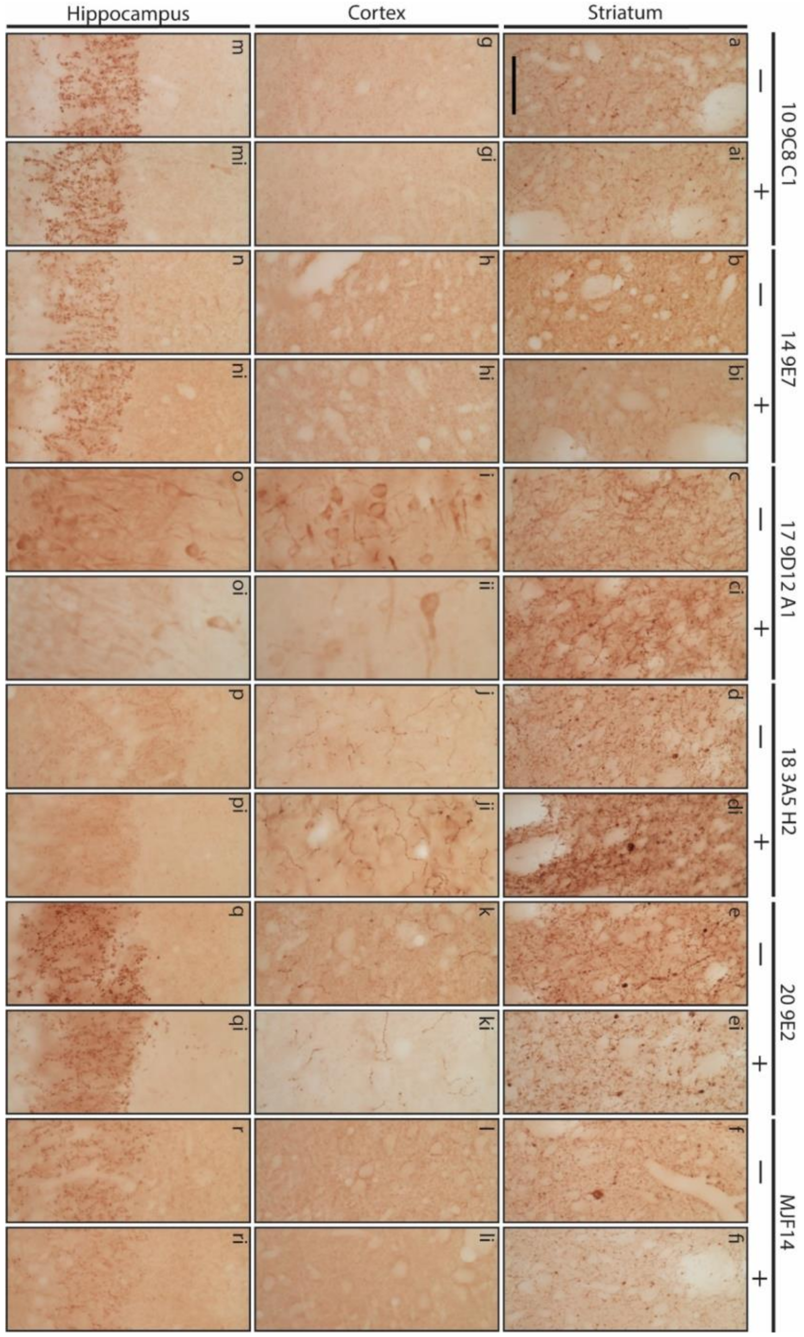

Figure S5. Pre-incubation of the mAbs with monomeric human α-syn. Staining of tissue using 5 mAbs generated in this study along with commercial antibody MJF4. Columns left to right show results for striatum, cortex, and hippocampus, respectively. For each antibody, the first row of figures are obtained without prestaining and the second row with preincubation, with monomeric α-syn. Scalebar=50µm.

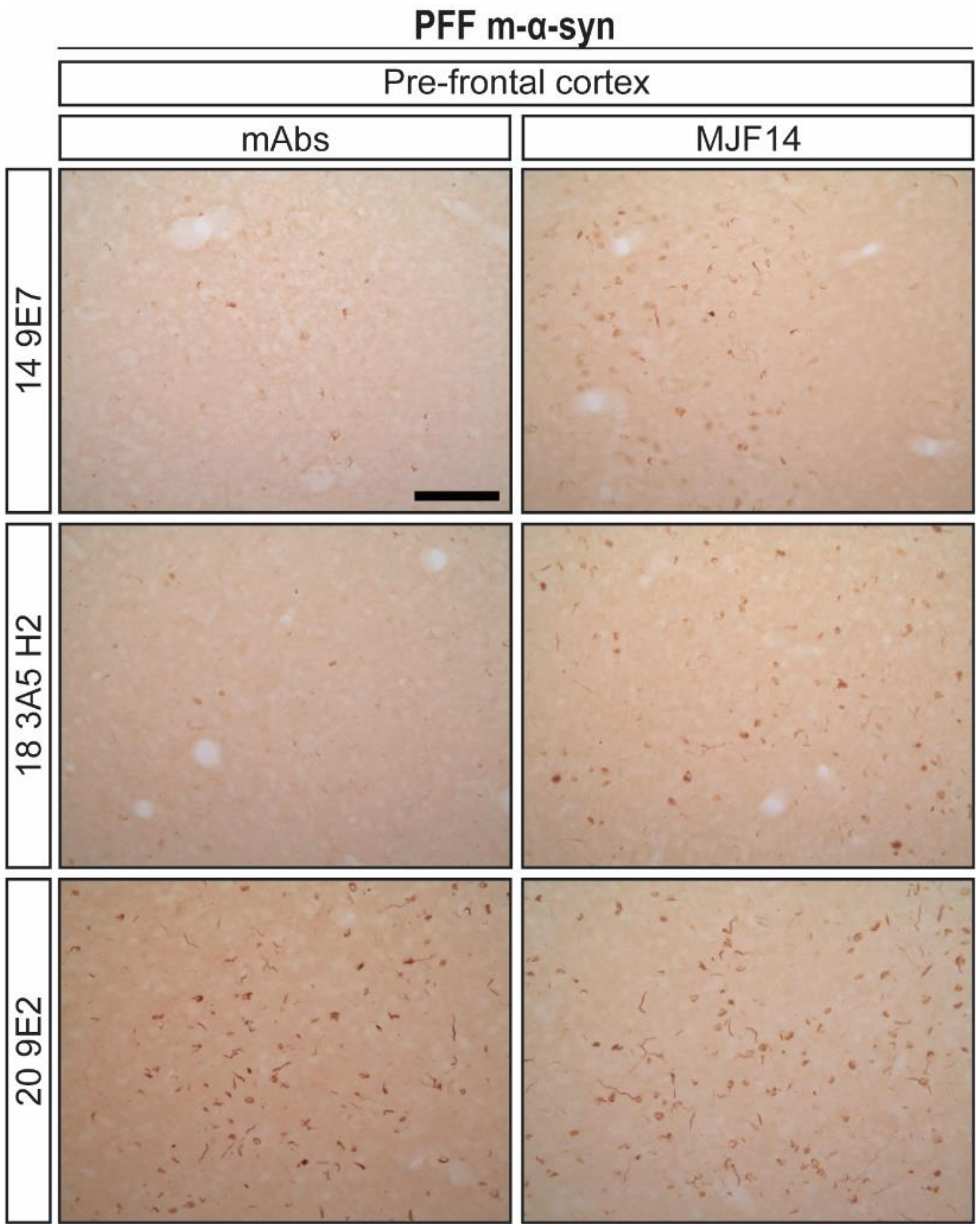

Figure S6

Figure S6. Prefrontal cortex of the PFF m-α-syn model. IHC done with mAbs (left column) or the commercial ab MJF14 (left column). Scalebar = 100µm.

Figure S7

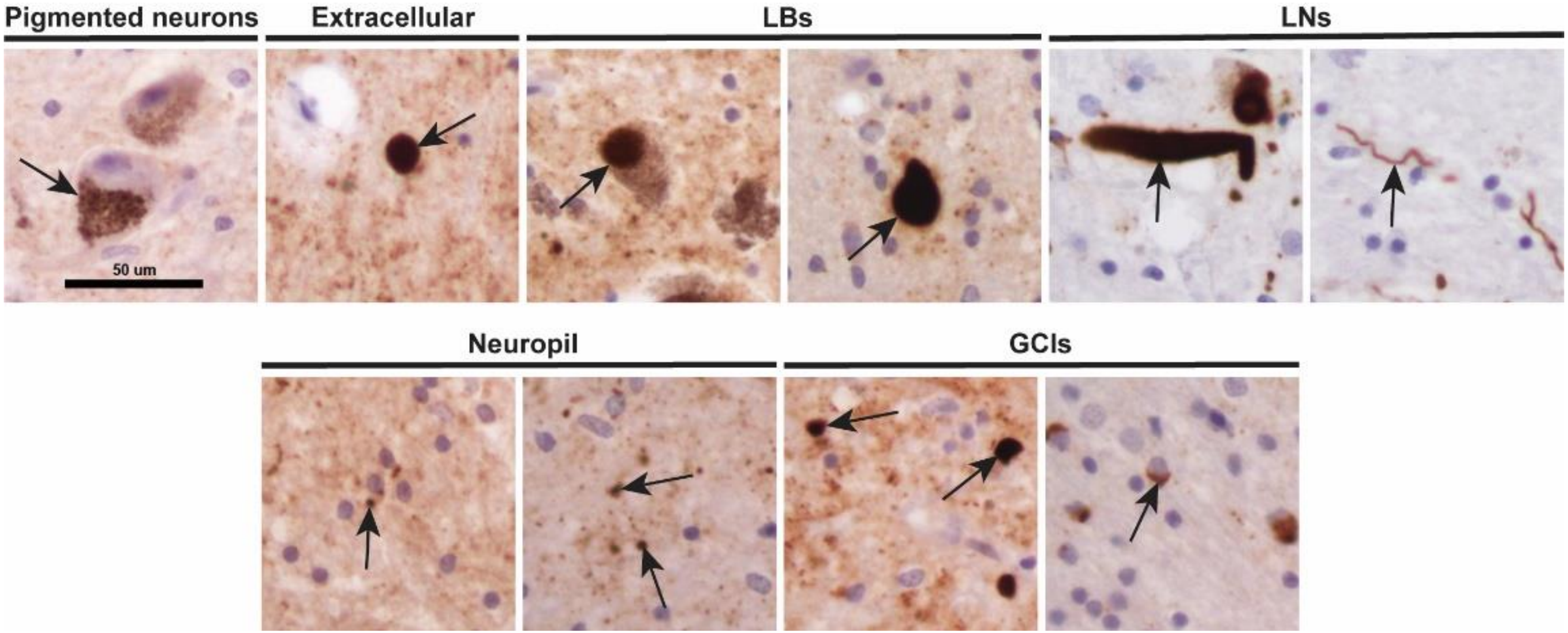

Figure S7. Prefrontal cortex of the PFF m-α-syn model. IHC done with mAbs (left column) or the commercial ab MJF14 (left column). Scalebar = 100µm.

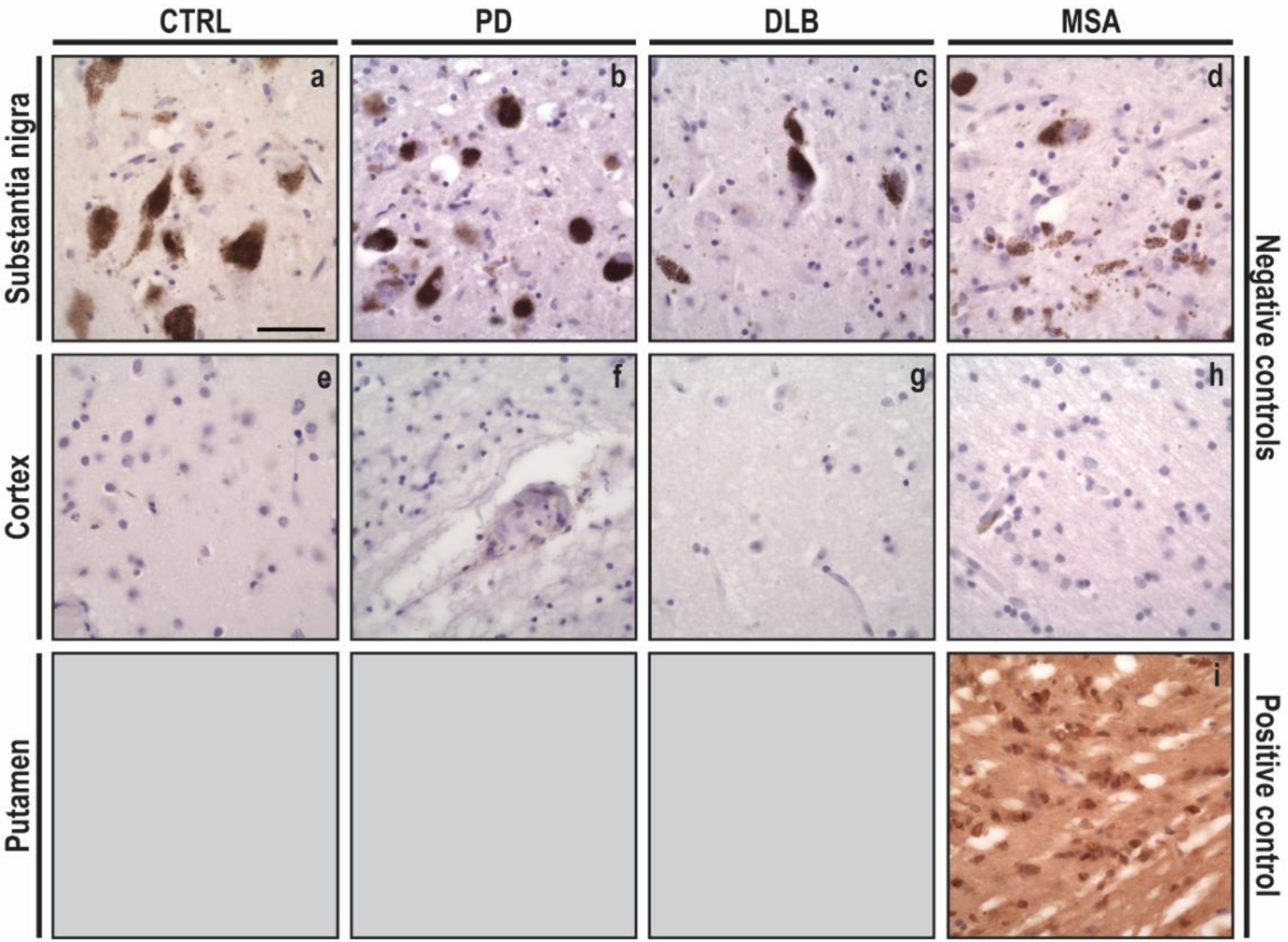

Figure S8

Figure S8. Positive and negative controls. a-d) Negative controls of substantia nigra for each group, omitting the primary mAb. **e-h)** Negative controls of the white matter cortex, omitting the primary mAb. **i)** Positive control of putamen from a MSA patient, stained with the commercial anti-α-syn antibody (Genetex). Scalebar = 50µm.
